# Green, facile synthesis and evaluation of unsymmetrical carbamide derivatives as antimicrobial and anticancer agents with mechanistic insights

**DOI:** 10.1038/s41598-024-65308-6

**Published:** 2024-07-04

**Authors:** Farid M. Sroor, Ahmed A. F. Soliman, Elham Mohamed Youssef, Mohamed Abdelraof, Ahmed F. El-Sayed

**Affiliations:** 1 Organometallic and Organometalloid Chemistry Department, National Research Centre, Cairo, 12622 Egypt; 2 Pharmacognosy Department, National Research Centre, Dokki, 12622 Egypt; 3 Biochemistry Department, National Research Centre, Giza, Egypt; 4 Microbial Chemistry Department, Biotechnology Research Institute, National Research Centre, Giza, Egypt; 5 Microbial Genetics Department, Biotechnology Research Institute, National Research Centre, Giza, Egypt; 6 Egypt Center for Research and Regenerative Medicine (ECRRM), Cairo, Egypt

**Keywords:** Unsymmetrical carbamide, Secondary amine, Isocyanate, Anti-cancer, Antimicrobial, Lipid peroxidation, Molecular docking, Biochemistry, Cancer, Cell biology, Chemical biology, Drug discovery, Chemistry

## Abstract

A very practical method for the synthesis of unsymmetrical carbamide derivatives in good to excellent yield was presented, without the need for any catalyst and at room temperature. Using a facile and robust protocol, fifteen unsymmetrical carbamide derivatives (**9–23**) bearing different aliphatic amine moieties were designed and synthesized by the reaction of secondary aliphatic amines with isocyanate derivatives in the presence of acetonitrile as an appropriate solvent in good to excellent yields. Trusted instruments like IR, mass spectrometry, NMR spectra, and elemental analyses were employed to validate the purity and chemical structures of the synthesized compounds. All the synthesized compounds were tested as antimicrobial agents against some clinically bacterial pathogens such as *Salmonella typhimurium*, *Bacillus subtilis, Pseudomonas aeruginosa*, *Staphylococcus aureus* and *Candida albicans*. Compounds **15**, **16**, **17**, **19** and **22** showed potent antimicrobial activity with promising MIC values compared to the positive controls. Moreover, compounds **15** and **22** provide a potent lipid peroxidation (LPO) of the bacterial cell wall. On the other hand, we investigated the anti-proliferative activity of compounds **9–23** against selected human cancerous cell lines of breast (MCF-7), colon (HCT-116), and lung (A549) relative to healthy noncancerous control skin fibroblast cells (BJ-1). The mechanism of their cytotoxic activity has been also examined by immunoassaying the levels of key anti- and pro-apoptotic protein markers. The results of MTT assay revealed that compounds **10**, **13**, **21**, **22** and **23** possessed highly cytotoxic effects. Out of these, three synthesized compounds **13**, **21** and **22** showed cytotoxicity with IC_50_ values (**13**, IC_50_ = 62.4 ± 0.128 and **22**, IC_50_ = 91.6 ± 0.112 µM, respectively, on MCF-7), (**13**, IC_50_ = 43.5 ± 0.15 and **21**, IC_50_ = 38.5 ± 0.17 µM, respectively, on HCT-116). Cell cycle and apoptosis/necrosis assays demonstrated that compounds **13** and **22** induced S and G2/M phase cell cycle arrest in MCF-7 cells, while only compound **13** had this effect on HCT-116 cells. Furthermore, compound **13** exhibited the greatest potency in inducing apoptosis in both cell lines compared to compounds **21** and **22**. Docking studies indicated that compounds **10**, **13**, **21** and **23** could potentially inhibit enzymes and exert promising antimicrobial effects, as evidenced by their lower binding energies and various types of interactions observed at the active sites of key enzymes such as Sterol 14-demethylase of *C. albicans*, Dihydropteroate synthase of *S. aureus*, LasR of *P. aeruginosa*, Glucosamine-6-phosphate synthase of *K*. *pneumenia* and Gyrase B of *B. subtilis*. Moreover, **13**, **21**, and **22** demonstrated minimal binding energy and favorable affinity towards the active pocket of anticancer receptor proteins, including CDK2, EGFR, Erα, Topoisomerase II and VEGFFR. Physicochemical properties, drug-likeness, and ADME (absorption, distribution, metabolism, excretion, and toxicity) parameters of the selected compounds were also computed.

## Introduction

**Figure 1:**
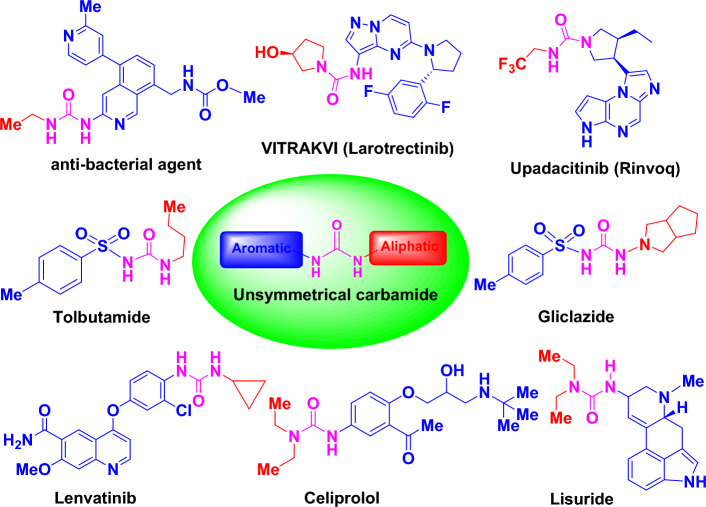
Structures of unsymmetrical carbamide containing FDA approved drugs.

Carbamide (commonly known as urea) and its derivatives are still inherent to explore pioneering bioactive compounds. Carbamide derivatives are safe, effective, and useful moieties with a significant biological activity profile^1–3^. Due to the ability of the carbamide subunit to form multiple stable hydrogen bonds with protein and receptor targets, carbamide (urea) and its derivatives (Fig. 1) have a pivotal role in drug candidates, agrochemicals, petrochemicals, pharmaceuticals, medicinal and bioorganic chemistry^4,5^. In the same line, unsymmetrical carbamide derivatives have exhibited significant biological activity in plenty of FDA-approved drugs (Fig. 1). The strategy of the combination of two fragments with proven biological properties in one skeleton is one of the most promising approaches in the design of novel organic compounds with targeted biological activities^6^. So the combination both of aliphatic (with hydrophilic properties) and aromatic (with hydrophobic properties) moieties in the presence of a carbamide functional core in one hybridized molecule could prove to be a promising compound with potent antimicrobial and anticancer properties (Fig. 1).

Development of the infectious diseases, particularly those caused by microbial pathogens still causes serious morbidity and mortality all over the world. The preparation of a new series of molecules based on active moieties exhibits one of the most significant ideas to supplement the classical antimicrobial agents. Carbamide derivatives were considered potent bioactive compounds when included in several medicinal applications such as anticancer, anti-diabetic, antiviral, and antibacterial agents including clinically approved therapeutics^5,7–9^. The resistance of the tested microbial pathogen toward the classical antibiotics was characterized previously since all bacterial pathogens were able to proliferate in the presence of Cephalosporin group such as Cephradine and Cephalexin, which revealed those having β-lactamase-encoding genes. Moreover, *Staphylococcus aureus* was considered as Methicillin Resistance *S. aureus* (MRSA), while *Klebsiella pneumonia,* and *Pseudomonas aeruginosa* were also characterized as Carbapenem resistance, which reflects their having Carbapenemase-encoding genes. Otherwise, the ability of the tested fungal strains (*Candida albicans* and *Aspergillus fumagitus*) to grow in the presence of Fluconazole was also noted, reflecting resistance to this group of antifungal agents. Thus, the virulence of these pathogens requires discovering new compounds that are triggered with a significant broad-spectrum antimicrobial activity.

Cancer is still the most urgent risk to human health for millions of people throughout the world, not only for low- and middle-income countries but for high-income countries as well^10^. Globally, breast cancer (BC) is the most often diagnosed cancer; over 2 million new cases were diagnosed with the disease in 2020, with over 680,000 deaths, it is the primary cause of cancer-related mortality among women^11,12^. In 2020, Over 2.3 million new cases and 685,000 people died deaths only from breast cancer, while it remains the most common type of female cancer in Egypt with an age-specific incidence rate of 48.8/105. Approximately 46,000 incident cases are forecasted in 2050^12,13^. Furthermore, it is currently the second leading cause of cancer mortality in Egypt, after hepatocellular carcinoma, with a mortality rate of 11% in 2020^14^.

On the other hand, colon cancer and rectal cancer are grouped under the term colorectal cancer which is a type of gastrointestinal malignancy^15–17^. This type of cancer occurs as a consequence of epigenetic and genetic mutations in the normal epithelium cells of the colon^18^. Moreover, colorectal cancer is one of the major cancer types causing cancer deaths globally^19^. Considering the most diagnosed cancers, colorectal cancer is the third in men and second in women^20^. In Egypt, Colon cancer represents the ninth most common cancer according to GLOBOCAN 2020, whereas rectal cancer ranks 18th^13,21^. The incidence of colon carcinoma is 2.27% while the incidence of rectal carcinoma is 2.08%^22–24^. Considering all these above-mentioned facts, we described in this study our efforts to use commercially available reagents in mild reaction and metal-free conditions to synthesize and characterize of unsymmetrical carbamate derivatives (**9–23**). Moreover, antimicrobial and anti-cancer activities as in vitro and in silico studies of all the prepared compounds were investigated and discussed.

## Materials and methods

All reactions were carried out at room temperature. The solvents acetonitrile (AC) or dichloromethane (DCM) were distilled before use. All glassware was oven-dried at 120 °C for at least 24 h before use. The aliphatic secondary amines (pyrrolidine (**1**), morpholine (**2**), *N*-methyl piperazine (**3**), piperidine (**4**) and the aliphatic primary amine, 1-Adamantylamine (**5**)) and the isocyanate derivatives (4-tolylysulfonyl isocyanate (**6**), 4-tolylisocyanate (**7**) and phenylisocyanate (**8**)) were purchased from Aldrich and used as received^1^. All melting points are uncorrected and measured using the Electro‐Thermal IA 9100 apparatus (Shimadzu, Japan). The Infrared spectra were recorded as potassium bromide pellets on a JASCO spectrophotometer between 4000 and 400 cm^−1^. ^1^H-NMR and ^13^C-NMR spectra were recorded in deuterated dimethylsulfoxide (DMSO-d_6_) on a Brucker spectrometer (400 MHz) or Joel (500 MHz) at 25 °C. The chemical shifts were expressed as part per million (*δ* values, ppm) using deuterated solvent signals as reference. Microanalyses were operated using the Mario Elmentar apparatus, Organic Microanalysis Unit, National Research Centre (NRC), Cairo, Egypt^1,25^.

### General procedure for the synthesis of unsymmetrical carbamide derivatives (9–23)

The isocyanates (**6**,** 7** or** 8**) (0.01 mol) were added to a solution of the amines (**1**–**5**) (0.01 mol) in acetonitrile (AC) (in case of compounds **13**, **18** and **23**, dichloromethane (DCM) was used as appropriate solvent) while stirring at room temperature. The reaction mixture was stirred to the desired time. The completion of reactions was monitored by TLC on silica gel-coated aluminum sheets^1^. The formed precipitate was filtered off, washed with cold acetonitrile and dried well, then recrystallized from ethyl acetate/acetonitrile (3:1) to give:

#### N-tosylpyrrolidine-1-carboxamide (9)

Compound **9** precipitated after 13 min as a white solid with a yield of 93% and mp 228–230 °C. IR (KBr, cm^−1^): 3267 (NH), 2976, 2935, 2879 (CH-aliph.), 1695 (C=O), 1367, 1090 (SO_2_). ^1^H NMR (DMSO-d_6_, 400 MHz): *δ* (ppm) 1.75–1.78 (m, 4H, 2CH_2_), 2.39 (s, 3H, CH_3_), 3.23–3.26 (m, 4H, 2CH_2_), 7.38 (d, 2H, *J* = 8.0 Hz, Ar–H), 7.82 (d, 2H, *J* = 8.0 Hz, Ar–H), 10.41 (br, 1H, NH). ^13^C NMR (DMSO-d_6_, 100 MHz): *δ* (ppm) 21.4, 25.2, 46.3, 128.0, 129.5, 138.6, 143.6, 150.9. Anal. For C_12_H_16_N_2_O_3_S (268.33): Calcd. C, 53.71; H, 6.01; N, 10.44. Found: C, 53.64; H, 5.98; N, 10.51.

#### N-tosylmorpholine-4-carboxamide (10)

Compound **10** precipitated after 18 min as a white solid with a yield of 94% and mp 210–211 °C. IR (KBr, cm^−1^): 3267 (NH), 2974, 2932, 2880 (CH-aliph.), 1690 (C=O), 1365, 1085 (SO_2_). ^1^H NMR (DMSO-d_6_, 500 MHz): *δ* (ppm) 2.33 (s, 3H, CH_3_), 2.80–2.83 (m, 4H, 2CH_2_), 3.56–3.60 (m, 4H, 2CH_2_), 7.21 (d, 2H, *J* = 5.0 Hz, Ar–H), 7.58 (d, 2H, *J* = 5.0 Hz, Ar–H). ^13^C NMR (DMSO-d_6_, 100 MHz): *δ* (ppm) 20.8, 44.6, 66.5, 126.8, 128.3, 140.7, 140.8, 154.5. Anal. For C_12_H_16_N_2_O_4_S (284.33): Calcd. C, 50.69; H, 5.67; N, 9.85. Found: C, 50.62; H, 5.81; N, 9.87.

#### 4-Methyl-N-tosylpiperazine-1-carboxamide (11)

Compound **11** precipitated after 25 min as a white solid with a yield of 82% and mp 190–192 °C. IR (KBr, cm^−1^): 3269 (NH), 2974, 2940, 2882 (CH-aliph.), 1696 (C=O), 1366, 1093 (SO_2_). ^1^H NMR (DMSO-d_6_, 500 MHz): *δ* (ppm) 2.16 (s, 3H, CH_3_), 2.37–2.40 (m, 7H, CH_3_ + 2CH_2_), 2.82–2.85 (m, 4H, 2CH_2_), 7.21 (d, 2H, *J* = 5.0 Hz, Ar–H), 7.58 (d, 2H, *J* = 5.0 Hz, Ar–H). ^13^C NMR (DMSO-d_6_, 100 MHz): *δ* (ppm) 20.9, 39.5, 44.2, 46.0, 53.6, 125.6, 126.9, 128.5, 140.8, 141.1, 154.6. Anal. For C_13_H_19_N_3_O_3_S (297.37): Calcd. C, 52.51; H, 6.44; N, 14.13. Found: C, 52.61; H, 6.57; N, 14.22.

#### N-tosylpiperidine-1-carboxamide (12)

Compound **12** precipitated after 20 min as a white solid with a yield of 78% and mp 232–235 °C. IR (KBr, cm^−1^): 3269 (NH), 2966, 2931, 2868 (CH-aliph.), 1691 (C=O), 1366, 1083 (SO_2_). ^1^H NMR (DMSO-d_6_, 500 MHz): *δ* (ppm) 1.40–1.43 (m, 8H, 4CH_2_), 2.38 (s, 3H, CH_3_), 3.23–3.27 (m, 2H, CH_2_), 7.35 (d, 2H, *J* = 5.0 Hz, Ar–H), 7.76 (d, 2H, *J* = 5.0 Hz, Ar–H), 10.75 (br, 1H, NH). ^13^C NMR (DMSO-d_6_, 100 MHz): *δ* (ppm) 20.9, 21.6, 22.1, 24.0, 25.5, 39.7, 43.8, 125.6, 127.0, 128.5, 129.2, 141.4, 141.8. Anal. For C_13_H_18_N_2_O_3_S (282.36): Calcd. C, 55.30; H, 6.43; N, 9.92. Found: C, 55.41; H, 6.49; N, 10.02.

#### N-(adamantan-1-ylcarbamoyl)-4-methylbenzenesulfonamide (13)

Compound **13** precipitated after 10 min (in DCM) as a white solid with a yield of 88% and mp 150–152 °C. IR (KBr, cm^−1^): 3425, 3343 (NH), 2911 (CH-aliph.), 1700 (C=O), 1371, 1132 (SO_2_). ^1^H NMR (DMSO-d_6_, 500 MHz): *δ* (ppm) 1.53– 1.58 (m, 6H, adamantyl), 1.75–1.77 (m, 6H, adamantyl), 1.96–2.05 (m, 3H, adamantyl), 2.38 (s, 3H, CH_3_), 7.27 (br., 1H, NH), 7.35–7.38 (m, 3H, Ar–H + NH), 7.72 (d, 2H, *J* = *8* Hz, Ar–H). ^13^C NMR (DMSO-d_6_, 100 MHz): *δ* 21.4, 28.8, 29.3, 36.4, 41.7, 126.1, 127.4, 128.9, 129.8, 129.5, 141.9, 142.3. Anal. For C_18_H_24_N_2_O_3_S: Calcd. C, 62.04; H, 6.94; N, 8.04. Found: C, 62.11; H, 6.91; N, 7.92.

#### N-(p-tolyl)pyrrolidine-1-carboxamide (14)

Compound **14** precipitated after 10 min as a white solid with a yield of 91% and mp 180–182 °C. IR (KBr, cm^−1^): 3265 (NH), 2972, 2930, 2882 (CH-aliph.), 1688 (C=O). ^1^H NMR (DMSO-d_6_, 500 MHz): *δ* (ppm) 1.80 (m, 4H, 2CH_2_), 2.28 (s, 3H, CH_3_), 3.31 (m, 4H, 2CH_2_), 6.99 (d, 2H, *J* = 5.0 Hz, Ar–H), 7.37 (d, 2H, *J* = 5.0 Hz, Ar–H), 7.97 (br, 1H, NH). ^13^C NMR (DMSO-d_6_, 100 MHz): *δ* (ppm) 20.8, 25.6, 46.2, 120.2, 129.1, 130.7, 138.6, 154.6. Anal. For C_12_H_16_N_2_O (204.27): Calcd. C, 70.56; H, 7.90; N, 13.71. Found: C, 70.67; H, 7.99; N, 13.85.

#### N-(p-tolyl)morpholine-4-carboxamide (15)

Compound **15** precipitated after 25 min as a white solid with a yield of 87% and mp 165–167 °C. IR (KBr, cm^−1^): 3262 (NH), 2971, 2928, 2866 (CH-aliph.), 1685 (C=O). ^1^H NMR (DMSO-d_6_, 400 MHz): *δ* (ppm) 2.23 (s, 3H, CH_3_), 3.41 (m, 4H, 2CH_2_), 3.60 (m, 4H, 2CH_2_), 7.05 (d, 2H, *J* = 12.0 Hz, Ar–H), 7.34 (d, 2H, *J* = 12.0 Hz, Ar–H), 8.42 (br, 1H, NH). ^13^C NMR (DMSO-d_6_, 100 MHz): *δ* (ppm) 20.3, 44.1, 66.0, 119.8, 128.7, 130.6, 137.7, 155.2. Anal. For C_12_H_16_N_2_O_2_ (220.27): Calcd. C, 65.43; H, 7.32; N, 12.72. Found: C, 65.43; H, 7.41; N, 12.79.

#### 4-Methyl-N-(p-tolyl)piperazine-1-carboxamide (16)

Compound **16** precipitated after 30 min as a white solid with a yield of 91% and mp 252–253 °C. IR (KBr, cm^−1^): 3265 (NH), 2971, 2932, 2875 (CH-aliph.), 1691 (C=O). ^1^H NMR (DMSO-d_6_, 500 MHz): *δ* (ppm) 2.15 (s, 3H, CH_3_), 2.18 (s, 3H, CH_3_), 2.24–2.27 (m, 4H, 2CH_2_), 3.34–3.37 (m, 4H, 2CH_2_), 7.00 (d, 2H, *J* = 5.0 Hz, Ar–H), 7.30 (d, 2H, *J* = 5.0 Hz, Ar–H), 8.37 (br, 1H, NH). ^13^C NMR (DMSO-d_6_, 100 MHz): *δ* (ppm) 20.9, 44.2, 46.3, 55.0, 120.3, 129.2, 131.0, 138.5, 155.7. Anal. For C_13_H_19_N_3_O (233.32): Calcd. C, 66.92; H, 8.21; N, 18.01. Found: C, 66.97; H, 8.33; N, 18.11.

#### N-(p-tolyl)piperidine-1-carboxamide (17)

Compound **17** precipitated after 25 min as a white solid with a yield of 82% and mp 230–232 °C. IR (KBr, cm^−1^): 3265 (NH), 2977, 2934, 2871 (CH-aliph.), 1684 (C=O). ^1^H NMR (DMSO-d_6_, 500 MHz): *δ* (ppm) 1.52 (m, 6H, 2CH_2_), 2.23 (s, 3H, CH_3_), 3.39 (m, 4H, 2CH_2_), 7.02 (d, 2H, *J* = 5.0 Hz, Ar–H), 7.32 (d, 2H, *J* = 5.0 Hz, Ar–H), 8.32 (br, 1H, NH). ^13^C NMR (DMSO-d_6_, 100 MHz): *δ* (ppm) 20.9, 24.7, 26.0, 45.2, 120.3, 129.2, 130.8, 138.7, 155.5. Anal. For C_13_H_18_N_2_O (218.30): Calcd. C, 71.53; H, 8.31; N, 12.83. Found: C, 71.66; H, 8.39; N, 12.89.

#### 1-(Adamantan-1-yl)-3-(p-tolyl)urea (18)

Compound **18** precipitated after 50 min as a white solid (in DCM) with a yield of 91% and mp 180–182 °C. IR (KBr, cm^−1^): 3265 (NH), 2981, 2930, 2868 (CH-aliph.), 1682 (C=O). ^1^H NMR (DMSO-d_6_, 400 MHz): *δ* (ppm) 1.63 (m, 6H, CH_2_), 1.92 (m, 6H, CH_2_), 2.03 (m, 3H, CH), 2.21 (s, 3H, CH_3_), 5.81 (s, 1H, NH), 6.99 (m, 2H, Ar–H), 7.21 (m, 2H, Ar–H), 8.12 (s, 1H, NH). ^13^C NMR (DMSO-d_6_, 100 MHz): *δ* (ppm) 20.2, 28.9, 36.0, 38.8, 41.7, 49.7, 117.4, 128.9, 129.3, 138.1, 154.0. Anal. For C_18_H_24_N_2_O (284.40): Calcd. C, 76.02; H, 8.51; N, 9.85. Found: C, 76.12; H, 8.60; N, 9.91.

#### N-phenylpyrrolidine-1-carboxamide (19)

Compound **19** precipitated after 10 min as a white solid with a yield of 89% and mp 233–235 °C. IR (KBr, cm^−1^): 3261 (NH), 2970, 2938, 2873 (CH-aliph.), 1685 (C=O). ^1^H NMR (DMSO-d_6_, 400 MHz): *δ* (ppm) 1.88–1.91 (m, 4H, 2CH_2_), 3.58–3.62 (m, 4H, 2CH_2_), 7.08 (br, 1H, Ar–H), 7.30 7.33 (m, 4H, Ar–H), 8.83 (s, 1H, NH). ^13^C NMR (DMSO-d_6_, 100 MHz): *δ* (ppm) 25.6, 124.9, 126.3, 128.3, 141.2, 178.1. Anal. For C_11_H_14_N_2_O (190.25): Calcd. C, 69.45; H, 7.42; N, 14.73. Found: C, 69.37; H, 7.48; N, 14.82.

#### N-phenylmorpholine-4-carboxamide (20)

Compound **20** precipitated after 45 min as a white solid with a yield of 82% and mp 200–202 °C. IR (KBr, cm^−1^): 3262 (NH), 2970, 2930, 2871 (CH-aliph.), 1689 (C=O). ^1^H NMR (DMSO-d_6_, 500 MHz): *δ* (ppm) 3.60–3.65 (m, 4H, 2CH_2_), 3.83–3.86 (m, 4H, 2CH_2_), 7.08 (br, 1H, Ar–H), 7.22–7.27 (m, 4H, Ar–H), 9.33 (s, 1H, NH). ^13^C NMR (DMSO-d_6_, 100 MHz): *δ* (ppm) 49.0, 66.3, 124.9, 125.8, 128.6, 141.5, 182.4. Anal. For C_11_H_14_N_2_O_2_ (206.25): Calcd. 64.06; H, 6.84; N, 13.58. Found: C, 64.12; H, 6.92; N, 13.51.

#### 4-Methyl-N-phenylpiperazine-1-carboxamide (21)

Compound **21** precipitated after 25 min as a white solid with a yield of 89% and mp 128–130 °C. IR (KBr, cm^−1^): 3266 (NH), 2968, 2934, 2882 (CH-aliph.), 1685 (C=O). ^1^H NMR (DMSO-d_6_, 500 MHz): *δ* (ppm) 2.18 (s, 3H, CH_3_), 2.33–2.35 (m, 4H, 2CH_2_), 3.85–3.88 (m, 4H, 2CH_2_), 7.08 (br, 1H, Ar–H), 7.24–7.28 (m, 4H, Ar–H), 9.28 (s, 1H, NH). ^13^C NMR (DMSO-d_6_, 100 MHz): *δ* (ppm) 46.0, 48.5, 54.8, 124.8, 125.7, 128.5, 141.6, 182.1. Anal. For C_12_H_17_N_3_O (219.29): Calcd. C, 65.73; H, 7.81; N, 19.16. Found: C, 65.78; H, 7.89; N, 19.28.

#### N-phenylpiperidine-1-carboxamide (22)

Compound **22** precipitated after 20 min as a white solid with a yield of 94% and mp 190–192 °C. IR (KBr, cm^−1^): 3262 (NH), 2968, 2935, 2884 (CH-aliph.), 1684 (C=O). ^1^H NMR (DMSO-d_6_, 500 MHz): *δ* (ppm) 1.50–1.58 (m, 6H, 3CH_2_), 3.81–3.85 (m, 4H, 2CH_2_), 7.06 (br, 1H, Ar–H), 7.22–7.25 (m, 4H, Ar–H), 9.16 (s, 1H, NH). ^13^C NMR (DMSO-d_6_, 100 MHz): *δ* (ppm) 24.46, 26.01, 49.76, 124.53, 125.56, 128.47, 141.83, 181.40, Anal. For C_12_H_16_N_2_O (204.27): Calcd. C, 70.56; H, 7.90; N, 13.71. Found: C, 70.62; H, 7.95; N, 13.82.

#### 1-(Adamantan-1-yl)-3-phenylurea (23)

Compound **23** precipitated after 20 min as a white solid (in DCM) with a yield of 93% and mp 160–162 °C. IR (KBr, cm^−1^): 3265 (NH), 2975, 2933, 2877 (CH-aliph.), 1684 (C=O). ^1^H NMR (DMSO-d_6_, 500 MHz): *δ* (ppm) 1.60–1.67 (m, 6H, 3CH_2_), 2.03–2.08 (m, 3H, CH), 2.21–2.26 (m, 6H, 3CH_2_), 7.07 (s, 1H, Ar–H), 7.21 (br, 1H, NH), 7.28–7.32 (m, 2H, Ar–H), 7.42–7.44 (m, 2H, Ar–H), 9.29 (s, 1H, NH). ^13^C NMR (DMSO-d_6_, 100 MHz): *δ* (ppm) 29.0, 35.9, 40.8, 53.2, 123.0, 123.7, 128.3, 139.5, 178.6. Anal. For C_17_H_22_N_2_O (270.38): Calcd. C, 75.52; H, 8.20; N, 10.36. Found: C, 75.66; H, 8.26; N, 10.45.

### Antimicrobial activity

The antimicrobial efficiency of the synthesized unsymmetrical carbamide derivatives was screened using standard clinical pathogens derived from Department of Microbiology and Immunology, Faculty of Medicine (Boys), Al-Azhar University. The tested microbial pathogens used in this study were as follows, two Gram-positive bacteria (*Bacillus subtilis*, *Staphylococcus aureus)*, two Gram-negative bacteria (*Pseudomonas aerginousea*, *klebsiella pneumoniae*) and two fungi (*Candida albicans, Aspergillus fumgitus).* Before each antimicrobial examination, pre-activation of each microbial pathogen was implemented using specific growth conditions. For bacterial ones, preinoculum was prepared using Nuitrent broth medium (Codalab, Spain) at 37 °C for 24 h. while the fungal pathogens were activated using Potato Dextrose broth (Codalab, Spain) at 28 °C for 48 h. Consequently, each tested pathogen justified its inoculum size based on Colony Forming Units (CFU) to be closed to 10^–6^ during all antimicrobial tests. The preparation of agar well diffusion test was then conducted based on^2,26^, since the targeted compounds were screened at a fixed concentration (20 µg/mL) in comparison to the reference antibacterial and antifungal agents such as Cephradine, Ciprofloxacin, Flucanazole, and Amphotrecine B. The most potent compounds were evaluated according to the inhibition zone diameter (mm) obtained around each microbial pathogen^1,27^.

### Determination of minimum inhibition concentration of the most active compounds

The most active compounds that observed a considered antimicrobial activity were then subjected to determine the Minimum Inhibition Concentration (MIC) using the microdilution method according to CLSI Protocol^28^. For this purpose, serial dilution of the potent compounds was carried out to obtain the desired concentrations (i.e. 10–400 µg/mL). In brief, a defined weight for each selected compound was dissolved in dimethylsulphoxide (DMSO) and used as a stock solution^29^. The determination of the MIC value for each targeted compound was known as the lowest concentration of each sample that yielded a minimum number of colony-forming units (CFU) compared to the untreated samples^28^.

### Effect of lipid peroxidation (LPO)

Lipid peroxidation of bacterial cell membranes was a remarkable tool indicating the oxidative stress that was caused by the tested compounds. The lipid peroxidation colorimetric assay kit was used for this purpose. Since, the detection of the lipid peroxidation byproduct, malondialdehyde (MDA) was performed by a specific reagent thiobarbituric acid (TBA) to yield a pink color complex, MDA-TBA^30^. In this regard, the efficient concentration of the most potent compounds was utilized to investigate the oxidation of the fatty acid contents in the bacterial cell membrane. Briefly, each tested compound was incubated with each bacterial pathogen overnight under shaking and then 1 mL of the treated bacterial cell was vigorously mixed with 300 μL MDA lysis buffer at 4 °C. The addition of 3 μL of Butylated hydroxytoluene (BHT) was then performed to avoid the pigment interference that resulted from the decomposition of lipophilic peroxides. Thereafter, samples were separated by centrifugation at 8000 rpm for 10 min, and the insoluble substances were discarded. Finally, 200 μL of the clear supernatant was mixed with 600 μL of the TBA solution at 95 °C for 60 min. Samples were then cooled at room temperature, and the developed pink color was determined at 532 nm using a Spectrophotometer (Agilent Cary 100, Germany). The positive control was also prepared using 5% of Hydrogen peroxide and treated each bacterial pathogen for 20 min. The noticed increase in the lipid peroxidation efficiency was expressed from the following equation:

$$\left( {{\text{Lipid}}\;{\text{peroxidation}}\;{\text{efficiency}}\;\left( \% \right) = \left[ {\left( {{\text{N}}_{{\text{C}}} - {\text{N}}_{{\text{S}}} } \right)/{\text{N}}_{{\text{C}}} } \right] \times {1}00} \right).$$

where N_C_ is the absorbance of lipid peroxidation in the untreated bacterial cells and N_S_ is the absorbance of lipid peroxidation in the treated bacterial cells.

### Anti-Cancer activity

#### Cell culture

The cell lines HCT-116 (human colon cancer), MCF-7 (human breast cancer), and A549 (human non-small cell lung cancer) were obtained from the Karolinska Institute in Stockholm, Sweden. The cells were all grown in RPMI 1640 medium. 10% heat-inactivated fetal bovine serum was added to the media, along with a 1% antibiotic-antimitotic mixture (10,000 U/mL potassium penicillin, 10,000 g/mL streptomycin sulfate, 25 g/mL amphotericin B, and 1% L glutamine (Biowest, USA). ATCC® CRL-2522TM provided BJ-1, a human skin fibroblast derived from the normal foreskin, in a frozen ampoule containing approximately 1 × 10^6^ cells per 1 mL volume. BJ-1 was grown in MEM with 2 mM L-glutamine and Earle's salts medium.

#### MTT cytotoxicity assay

The MTT 3-(4,5-dimethylthiazol-2-yl)-2,5-diphenyl-tetrazolium bromide (Bio Basic Canada Inc. Toronto, Canada) assay was used to assess cell viability^27^. The procedures were carried out in a biosafety class II sterile laminar air flow cabinet (Baker, SG403INT; Sanford, ME, USA). All incubations were performed at 37 °C in a 5% CO_2_ incubator with a 95% humidified environment (Sheldon, TC2323; Cornelius, OR, USA). Cells were seeded at a density of (10^4^ cells/well) into 96-well micro titer polypropylene plates and left to adhere for 24 h. The media was aspirated and the test chemicals were given to the cells in a single dose of 100 µM in DMSO. After 48 h of incubation, 40 L of MTT salt (2.5 g/mL) was applied to each well. 200µL of 10% sodium dodecyl sulfate (SDS) was added to each well and incubated for 2 h at 37 °C to terminate the reaction and dissolve any generated formazan crystals. A microplate reader (Bio-Rad Laboratories, model 3350, California, USA) was used to measure the amount of formazan product at 595 nm with a reference wavelength of 690 nm as a background. Instead of the tested substances, the medium was applied to the untreated cells (negative control). A known cytotoxic natural substance, adrinamycin® (doxorubicin, Mr = 579.9) (Pharmacia India Pvt Ltd. Gurgaon, Haryana 122001, India), was employed as a positive control. The testing substance was dissolved in dimethylsulfoxide (DMSO), and its final concentration in the cells was less than 0.2%. The solvent concentration was the same for all medications and between the control and drug treatments at the same dilution. By applying different concentrations of 0, 6.25, 12.2, 25, and 50 µM (three replicates), and the concentration required for 50% inhibition of cell viability (IC_50_) was estimated for the powerful compounds that demonstrated preliminary cytotoxic effects at 100 µM.

### Cell cycle analysis and necrosis cell apoptosis assay of treated MCF7 & HCT116 cells

#### Cell cycle assessment

The cell cycle distributions in MCF-7 treated by compounds (**13** and **22**) and HCT116 treated by compounds (**13** and **21**) were analyzed by flow cytometry analysis using PI staining assay. The cells were treated with IC_50_ concentrations of tested compounds for 48 h, then were trypsinized, washed in 0.5 mL 1 × DPBS and fixed with 70% ethanol on ice for 2 h. The ethanol-suspended cells were centrifuged and re-suspended in 5 mL 1 × DPBS, then centrifuged again before being re-suspended in 1 mL of PI staining solution. The stained cells were kept in the dark at room temperature for 30 min before being transferred to the CytoFLEX Flow Cytometer to measure their fluorescence. The percentage of cells in different phases of the cell cycle (G0/G1, S, and G2/M) was determined using CytExpert Software. BECKMAN COULTER Inc., Cairo, Egypt, Cat. No. 4238055-CB was used in this assay^25^.

#### Necrosis cell apoptosis assay

The levels of necrosis and apoptosis in cells were determined by CytoFLEX Flow Cytometer. A total of 1 × 106 cells were washed in 1 × DPBS, and then were centrifuged at 500 × ϲ for 5 min. 1 μL of annexin V-FITC solution and 5 μL of dissolved PI were added to 100 μL of the cell suspensions that kept on ice and were incubated for 15 min in the dark. Cells were then analyzed by the CytoFLEX Flow Cytometer (Beckman Coulter Life Sciences, USA) and the percentage of apoptotic cells was determined using CytExpert Software.

## Computational methods

### Molecular docking simulation

#### Preparation of protein receptors

To inspect the antifungal activity of the isolated compounds, the sterol 14-demethylase of *C.albicans* (PDB: 5TZ1), Dihydropteroate synthase of *S. aureus* (PDB: 1AD4), Crystal Structure of Gyrase B of *Bacillus subtilis* (PDB: 4URM), LasR an activator of exotoxin in *P. aeruginosa* (PDB: 2UV0), and Glucosamine-6-phosphate synthase of *Salmonella typhimurium* (PDB: 2VF5) were downloaded from RCSB Protein Data Bank (Table 1). All crystal structures of the target were processed by removing water molecules, ions, and existing ligands that were removed using PyMOL software (After that, the addition of hydrogen atoms to the receptor molecule was carried out by using MG Tools of Autodock Vina^31^. This process was repeated for each protein and was subsequently saved into a dockable *pdbqt* format for molecular docking. Complexed inhibitors were separated from the crystal structures to be used as control ligands.

**Table 1:** List of targets of bacterial proteins, PDB IDs, active site coordinates, Native Ligands, and Reference.

Type	Organism		Protein Targets	PDB ID	Active site coordinates			Reference Ligands	Native Ligand ID
					**X**	**Y**	**Z**		
Fungi	*C. albicans*	–	Sterol 14-demethylase	**5TZ1**	70.7	64.28	4.68	**Miconazole**	**VT1**
	*A. fumagitus*	–	FDC1 gene	**4ZA5**	–	–	–	**Miconazole**	–
Bacteria	*B. subtilis*	**G + ve**	Gyrase B	**4URM**	40.3	1.52	8.17	**Ciprofloxacin**	**XAM**
	*K. pneumonia*	**G-ve**	Glucosamine-6-phosphate synthase	**2VF5**	4.36	33.47	− 14.16	**Ciprofloxacin**	**GLP**
	*S. aureus*	**G + ve**	Dihydropteroate synthase	**1AD4**	33.4	5.95	37.9	**Ciprofloxacin**	**HH2**
	*P. aeruginosa*	**G-ve**	LasR an activator of exotoxin	**2UV0**	24.37	13.79	81.52	**Ciprofloxacin**	**OHN**

### Preparation of ligands

The compounds’ Structure Data Format (SDF) was retrieved from the PubChem database. Using an Open Babel^32^, each compound was transformed into a mol2. Additionally, the polar hydrogen charges were assigned using the Gasteiger method, and the internal degrees of freedom and torsions were optimized to be at their minimum values. Molecules were converted to the *pdbqt* format using Autodock tools. Chemical structures were subjected to MM2 energy minimization.

### Docking studies

Before docking, polar-H atoms were added to the target followed by Gasteiger charges calculation using Autodock tools. The macromolecule file was saved in *pdbqt* format to be used for docking. Ligand-centered maps were generated by the AutoGrid program and grid dimensions of 90A° × 90A° × 90A°. Default settings were used for all other parameters. Using Autodock Vina^31^, all the docking experiments were done using the active site coordinates Table 1 (using a grid box large enough to cover the whole protein structure to encounter any possible protein–ligand interactions), calculation of the grid maps was performed using Auto Grid to save a lot of time during docking.

### Molecular interaction and visualization

Analysis of the 2-D hydrogen-bond interactions of the target-ligand structure was performed by the Discovery Studio 4.5 program, it depicts hydrophobic bonds, hydrogen bonds, and their bond lengths in each docking pose graphically.

### Physicochemical Parameters, in silico pharmacokinetics, metabolism, and toxicity

The physicochemical parameters of the synthesized compounds were calculated using the BIOVIA Discovery Studio software. Also, the ADME (absorption, distribution, metabolism, excretion, and toxicity) of the compound were predicted based on Lipinskis Rule of Five^33^.

## Results and discussion

### Chemistry

**Figure 2:**
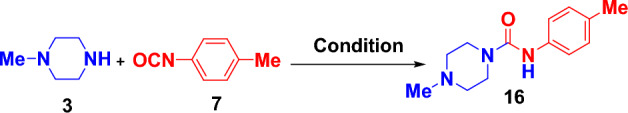
Optimization of reaction conditions for the synthesis of **16**.

The current work reports our effort to synthesize and structural details of unsymmetrical carbamate derivatives **9**–**23** under mild reaction conditions. As preliminary experimentation, we investigated the best reaction condition optimization via the synthesis of 4-methyl-*N*-(*p*-tolyl)piperazine-1-carboxamide (**16**) (Fig. 2) by the reaction of *N*-methyl piperazine (**3**) (1 mmol) and 4-tolylisoncyanate (**7**) (1 mmol) under different conditions as a model reaction and the experimental results for exploring reaction conditions are summarized in Table 2. In this optimized condition reaction, we used the available catalysts in our lab (i.e. CuI, CuCl_2_, SSA, TEA and DMAP). As compiled in Table 2, the obtained results for the synthesis of **16** confirmed that the optimized yield (91%) was observed by using acetonitrile (AC) as a solvent and without any catalyst at room temperature in 30 min (entry 8), while using dichloromethane (DCM) came in the second position with yield 79% in 30 min (entry 9). On the other hand, catalyst screening using different catalyst types and toluene as solvent was performed (Table 2, entries 1–5). The best among all the screened catalysts was TEA with a yield of 64% in 60 min (entry 4), while the CuI came in the second position with 61% in 60 min (entry 1). Likewise, solvent screening without using any catalyst was performed (entries 6–11). It is important the note that, Buchwald and co-workers reported the preparation of **16** using palladium-catalyzed cross-coupling of aryl chlorides and triflates with sodium cyanate with a yield of 78%^34^. We purposed to employ both polar and non-polar solvents in this study to investigate the effect of each type of these solvents. In summary, when we used both acetonitrile (AC) and dichloromethane (DCM) as solvents to achieve the target compound, the yield was improved significantly in a short time. However, pyridine and THF gave the lowest yield of the target compound with 34% (entry 7) and 31% (entry 11).

**Table 2:** Effect of catalyst, solvent and temperature on the model reaction of synthesis of 4-methyl-*N*-(*p*-tolyl)piperazine-1-carboxamide (**16**).

Entry	Catalyst (mol)	Solvent	Temperature (°C)	Time (min)	Yield (%)^a^
1	CuI (0.10)	Toluene	100	60	61
2	CuCl_2_ (0.10)	Toluene	100	60	52
3	SSA (0.10)	Toluene	100	60	54
4	TEA (0.10)	Toluene	100	60	64
5	DMAP (0.10)	Toluene	100	60	45
6	–	Toluene	100	60	57
7	–	Pyridine	100	60	34
8	–	**AC**	**r.t**	**30**	**91**
9	–	DCM	r.t	30	79
10	–	DMF	r.t	30	42
11	–	THF	r.t	60	31

^a^Isolated yield. The bold values were selected as optimum conditions for the next study.

AC, Acetonitrile; SSA, Silica sulfuric acid; TEA, Triethylamine; DMAP, 4-Dimethylaminopyridine and r.t, room temperature.

Thus, *N*-methyl piperazine (**3**) and 4-tolylyisoncyanate (**7**) in acetonitrile (AC) at room temperature in 30 min was concluded the optimized reaction protocol for the synthesis of target carbamide **16** without any catalyst and this was used throughout the envisaged protocol to synthesize all of the targeted unsymmetrical carbamide derivatives **9–23**.

The chemical structure of compound **16** was characterized by ^1^H NMR, ^13^C NMR, and FT-IR spectral techniques as well as elemental analyses. The IR confirmed the presence of the carbonyl group with a peak at 1691 cm^−1^, while the peak at 3265 cm^−1^ could be attributed to the NH group. In the ^1^H NMR spectrum, the methyl groups were assigned as singlet signals at *δ* 2.15 and 2.18 (ppm), while the methylene groups were observed as multiplets at *δ* 2.24–2.27 and 3.34–3.37 (ppm). All the carbons of **16** were observed in ^13^C NMR spectrum, carbons of the methyl groups were observed at *δ* 20.9 and 44.2 (N-CH_3_) (ppm), while carbon of the carbonyl group was reported at *δ* 155.7 (ppm).

**Figure 3:**
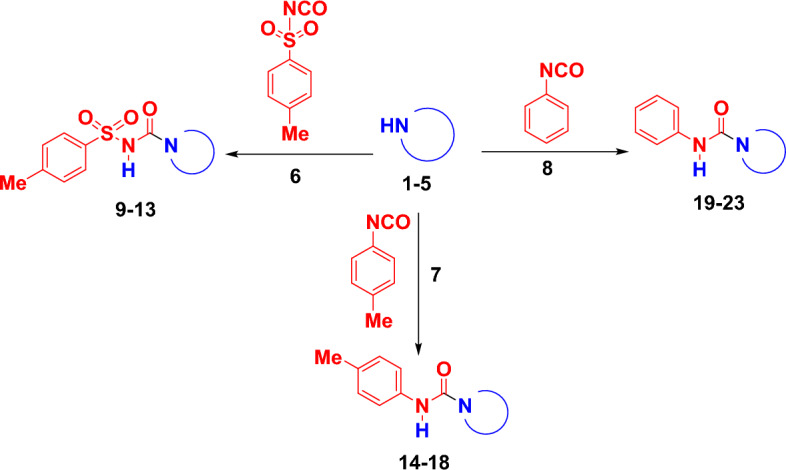
Synthesis of unsymmetrical carbamide derivatives (**9–23**). All the reactions were carried out in acetonitrile (AC) or dichloromethane (DCM) at room temperature.

**Figure 4:**
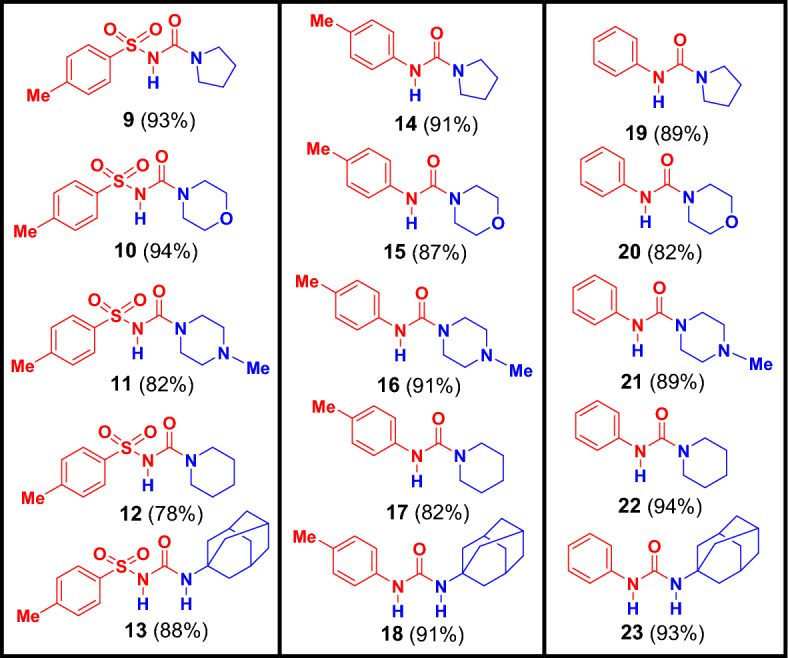
The targeted unsymmetrical carbamide derivatives (**9**–**23**).

After establishing the standard optimized reaction conditions for the synthesis of unsymmetrical carbamide **16**, we next demonstrated the general validity of the protocol through this envisaged methodology across access to a range of unsymmetrical carbamide derivatives **9–23** as shown in Figs. 3 and 4.

We used the available aliphatic secondary amines; pyrrolidine (**1**), morpholine (**2**), *N*-methyl piperazine (**3**), piperidine (**4**) and the aliphatic primary amine, 1-Adamantylamine (**5**) to react with three different isocyanates, 4-tolylsulfonyl isocyanate (**6**), 4-tolylisocyanate (**7**), and phenyl isocyanate (**8**). All the reactions proceeded smoothly in acetonitrile as solvent except the reaction of 1-Adamantylamine (**5**) with all the isocyanates which was carried out in dichloromethane due to the poor solubility of **5** in acetonitrile. The amines (**1–5**) were reacted with 4-toluenesulfonyl isocyanate (**6**) to afford the corresponding unsymmetrical carbamide derivatives (**9–13**) in good to excellent yield (78–94%) (Fig. 4). Compound **9** was investigated by our research group as potent antidiabetic agent^3^. These compounds were confirmed by studying their spectral data such as NMR, IR and elemental analyses. As a representative example, the spectral data of compound **11** will discussed in detail. The ^1^H-NMR spectrum showed a singlet signal at *δ* 2.16 (ppm) for the methyl group, while the methylene and methyl groups of the N-methylpiperazine ring were assigned at *δ* 2.37–2.40 and 2.82–2.85 (ppm) as multiplet signals. In the ^13^C-NMR, the carbon of the carbonyl group was detected at *δ* 154.6 (ppm), while the carbons of the methyl and the methylene groups were assigned at* δ* 20.9, 39.5, 44.2 and 46.0 (ppm), respectively. The IR showed the presence of the C = O group at band 1696 (cm^−1^) and the NH group at 3269 (cm^−1^), while the SO_2_ group was observed at 1366, 1093 (cm^−1^). It is worth to noting that, compound **10** was prepared by Zhang and co-workers through the reaction of 4-toluenesulfonyl chloride and morpholine in the presence of 1 mol % Pd(OAc)_2_ under a carbon monoxide atmosphere (1 atm) with a yield of 93%^35^.

Likewise, the reaction of the amines (**1–5**) with 4-tolylisocyanate (**7**) afforded the corresponding unsymmetrical carbamide derivatives (**14–18**) in very good to excellent yield (82–91%) (Fig. 4). The spectral data of compound **16** was discussed in more detail in the optimization reaction conditions as an example for this series of unsymmetrical carbamide compounds (**14–18**). It is important to report that, compounds **14, 15, 16** and **17** were prepared in previous literature using different catalysts and vigorous reaction conditions such as titanium(IV) complex^36^, binuclear aluminum complex^37^, lanthanum(III) trifluoromethane sulfonate^38^, bromodimethylsulfonium bromide^39^, phenyl 4,5-dichloro-6-oxopyridazine-1(6H)-carboxylate as a carbonyl source^40^, TEA and reflux for 36 h^41^, while, Patil et al.were prepared compound **18** using toluene as a solvent without catalyst at 40–45 °C with yield 79%^5^.

The last series of unsymmetrical carbamide derivatives (**19–23**) was obtained by the reaction of amines (**1–5**) with phenyl isocyanate (**8**) to give the corresponding unsymmetrical carbamide derivatives (**19–23**) in very good to excellent yield (82–94%) (Fig. 4). The chemical structure of unsymmetrical carbamide compounds (**19–23**) was elucidated by their spectral data. As a representative example, the spectral data of compound **23** will discussed in detail. In the IR spectrum, the NH group was assigned at band 3265 (cm^−1^) and the C=O group at band 1684 (cm^−1^), while the CH of the adamantyl ring was detected at bands 2975, 2933 and 2877 (cm^−1^). The ^1^H-NMR showed the 15 protons of the adamantyl ring at range of *δ* 160–2.26 (ppm), while the aromatic protons appeared in the aromatic zone at range of *δ* 7.07–7.44 (ppm). In the ^13^C NMR, the aliphatic carbons of the adamantyl ring reported at *δ* 29.0, 35.9, 40.8, 53.2 (ppm) and the carbon of the carbonyl group appeared at *δ* 178.6 (ppm). Some of these compounds were prepared in the previous literature using different catalysts under vigorous reaction conditions with dissimilar yields^36,38,40–44^. In 2021, Zhu et al.reported catalyst-free and mild conditions for preparation compounds **19, 22** and **23**^45^.

Finally, as a comparison with relevant literature that employs similar reactions of secondary aliphatic amines with isocyanate derivatives to prepare unsymmetrical carbamide derivatives, our current synthetic strategy is characterized by using no catalyst, excellent yield, limited time and all the reactions were carried out at room temperature. For example, Bano et al.^37^ reported the preparation of some urea derivatives via the reaction of isocyanates with secondary amines using a binuclear aluminium complex as a catalyst at room temperature for one hour, while our current study confirmed that these compounds could be prepared in AC or DCM without catalyst with maximum time 50 min. On the other hand, Mistry et al.^42^ prepared compounds **19**, **20** and **22** by the reaction of phenyl isocyanate with the corresponding amines in the presence of Cyrene as green solvent at 0 °C to room temperature for one hour to give these compounds in yield 80%, 64% and 61%, respectively, while we prepared these compounds in yield 89% (in 20 min), 82% (in 45 min) and 94% (in 10 min), respectively.

### Antimicrobial activity

#### Antimicrobial efficiency of unsymmetrical carbamide derivatives (9–23)

The antimicrobial activity evaluation of the synthesized unsymmetrical carbamide derivatives (**9–23**) was carried out according to the agar well diffusion procedure. Noticeably, variation of the microbial response toward the targeted compounds was quite obtained as shown in Figure S1, see supplementary information. In which, it was a remarkable number of the tested compounds had a potent antimicrobial susceptibility. In this way, compounds **15**, **16**, **17**, **19** and **22** were proved to be characterized as potent antimicrobial agents (Table 3) showing a satisfied inhibition zone around each pathogen nearing the potent antibacterial (Ciprofloxacin) and antifungal (Amphotericin B) agents (Table 2). Otherwise, our findings demonstrated the ability of some derivatives to represent a strong eradication against the Gram-Positive bacterium like, **9**, **14** and **23**. Meanwhile, **20** only was found to be providing a significant inhibition activity against Gram-negative bacteria, *Klebsiella pneumonia* (4 mm) and *Pseudomonas aeruginosa* (3 mm). Likewise, the highest sensitivity of *Candida albicans* towards the targeted compounds was proved to be more than that observed against *Aspergillus fumagitus.* On the other hand, little compounds are characterized as weak antimicrobial agents such as **11**, **18** and **21**. In comparison, compounds **15**, **16**, **17**, **19** and **22** provided considerable antimicrobial activity compared to the reference antibiotic agents. Therefore, the minimum inhibition concentration (MIC) for the most promising compounds **15**, **16**, **17**, **19**, and **22** against each pathogen was subsequently determined using different concentrations from 5 to 400 µg/mL.

**Table 3:** Antimicrobial activity of the targeted compounds using agar-well diffusion.

Compound	Inhibition Zone (mm)					
	*Candida albicans* (Unicellular fungi)	*Aspergillus fumagitus* (Multicellular fungi)	*Bacillus subtilis* (Gram-positive bacteria)	*Staphylococcus aureus* (Gram-positive bacteria)	*Klebsiella pneumonia* (Gram-negative bacteria)	*Pseudomonas aeruginosa* (Gram-negative bacteria)
**9**	ND^c^	2	6	4	2	2
**10**	ND	2	2	4	2	ND
**11**	ND	ND	ND	2	2	ND
**12**	2	ND	ND	3	3	ND
**13**	3	ND	6	2	2	3
**14**	8	3	4	3	2	ND
**15**	4	2	2	2	4	4
**16**	5	3	2	2	5	3
**17**	2	3	5	2	3	3
**18**	2	3	2	ND	ND	ND
**19**	4	3	2	2	4	2
**20**	3	2	2	ND	4	3
**21**	3	2	4	ND	3	2
**22**	5	4	5	2	3	3
**23**	3	ND	3	2	2	ND
Fluconazole^a^	ND	ND	–			
Amphotericin B^a^	4	5	–			
Ciprofloxacin^b^		–	5	3	5	3
Cephradine^b^		–	ND	ND	ND	ND

^a^Fluconazole and Amphotericin B were used as standard antifungal agents at 20 µg/mL.

^b^Ciprofloxacin and Cephradine were used as standard antibacterial agents at 20 µg/mL.

^c^ND, not determined.

### Determination of MIC value of the most promising compounds

In order to determine the greatest concentration that can trigger the lowest growth of each pathogen, the MIC value was investigated for each pathogen. As can be seen in (Table 4), all compounds showed MIC values against *Candida albicans* not exceeding 20 µg/mL. Therefore, some compounds become more active than the potent antifungal agent (Amphotericin B). Surprisingly, the promising eradication of *Candida albicans* and *Aspergillus fumagitus* at lower concentrations of all compounds elucidated their potent candidates as antifungals.

**Table 4:** Minimum inhibitory concentration (MIC) of the most promising compounds.

Compound	Minimum inhibitory concentration (MIC, µg/mL)					
	*Candida albicans* (Unicellular fungi)	*Aspergillus fumagitus* (Multicellular fungi)	*Bacillus subtilis* (Gram-positive bacteria)	*Staphylococcus aureus* (Gram-positive bacteria)	*Klebsiella pneumonia* (Gram-negative bacteria)	*Pseudomonas aeruginosa* (Gram-negative bacteria)
**15**	10	20	40	40	20	20
**16**	10	80	160	320	10	160
**17**	20	20	10	80	80	40
**19**	10	40	80	160	40	80
**22**	5	10	10	40	40	80
Fluconazole^a^	> 400	> 400	–			
Amphotericin B^a^	40	20	–			
Ciprofloxacin^b^	–	–	40	80	40	40
Cephradine^b^			320	> 400	> 400	> 400

^a^Fluconazole and Amphotericin B were used as standard antifungal agents at 20 µg/mL.

^b^Ciprofloxacin and Cephradine were used as standard antibacterial agents at 20 µg/mL.

In addition, compounds **15** and **17** also possessed MIC values close to that demonstrated for the same reference against *Aspergillus fumagitus* which reflected their antifungal potency. On the other hand, the greatest MIC value against Gram-negative bacteria was indicated for compound **15**, which provides the lowest proliferation of both tested strains at 20 µg/mL. Furthermore, the lower MIC value of compound **15** was found to be carried out against Gram-positive bacteria. From the MIC experiment, compounds, **15** and **22** represent a maximum activity at a lower concentration, in some times to be more efficient the standard drugs. Compound **15** was proved to be more active than the standard antibacterial agent with 2–3 times higher towards bacterial pathogen. In addition, compounds **16** and **19** showed higher MIC values against all microbial pathogens except for *Candida albicans* (10 µg/mL) and *Klebsiella pneumonia* (40 and 10 µg/mL, respectively). The significant response of the tested compounds, particularly **15** and **22** with lower MIC values against the multi-drug resistant microorganisms reflected the importance of these compounds to incorporate in the medicinal industries. It is important to note that, Zhu and co-workers reported the antibacterial and antifungal activities of compounds **15** and **20**^41^.

The potential application of urea derivatives as an antimicrobial agent was implemented by many authors. Screening of the unsymmetrical carbamide derivatives in the antimicrobial investigation was applied using standard clinical multi-drug resistance pathogens, which extensively observed new potential compounds against bacterial (*S. aureus* MRSA, *P. aeruginosa*), fungal (*C. albicans*) and tubercular (*M. tuberculosis*) pathogens^7^. Furthermore, a plausible activity of the aryl urea compounds was obtained against *Staphylococcus aureus*, *Escherichia coli*, and *Klebsiella pneumonia* with MIC values 32, 64, and 32 µg/mL, respectively^46^. By our findings, the antimicrobial activity of a novel series of piperazinyl-urea derivatives doped with pyrazole-4 carboxylic acid showed significant inhibition ability towards *E. coli*, *Y. enterocolitica*, *B. cereus*, *S. aureus* and *C. albicans*^47–50^. In addition, the synthesis of new urea derivatives based on aryl moieties and screening of their antimicrobial susceptibility demonstrated excellent activity against *Escherichia coli, Klebsiella pneumoniae, Acinetobacter baumannii, Pseudomonas aeruginosa*, *Staphylococcus aureus*, *Candida albicans* and *Cryptococcus neoformans*^5^. Novel urea derivative-loaded PLGA nanoparticles were prepared by Zhang et al.^51^ and investigated its inhibitory activity towards the cariogenic bacterial strain *Streptococcus mutans* UA159, which observed a potent anti-caries agent. Moreover, a new class of benzazolyl azolyl urea derivatives displayed a potential antimicrobial activity against *Bacillus subtilis* and *Aspergillus niger*^52^. Likewise, the antimicrobial activity of pyrimidinyl benzazolyl urea derivatives was indicated against *Bacillus subtilis* and *Aspergillus niger*^53,54^.

### Evaluation of lipid peroxidation (LPO)

**Figure 5:**
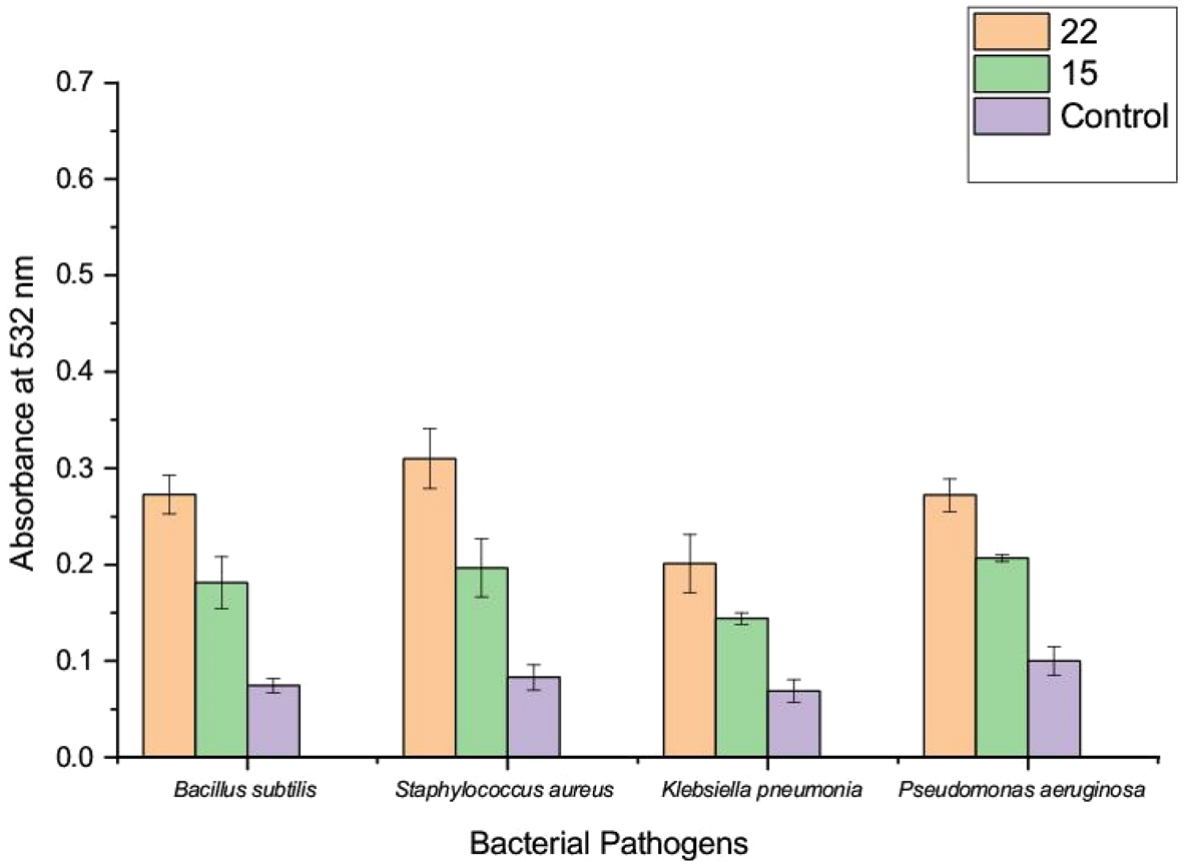
Assessed the lipid peroxide for the treated bacterial cell membrane.

Subsequently, the most potent compounds that make the highest inhibition activity with lower MIC values (i.e. **15** and **22**) were subjected to investigate their oxidative stress activity against the bacterial cells. Oxidation of Fatty acid content in the bacterial cell membrane after being treated by the tested derivatives was indicated by TBA that combined with MDA and formed the colored MDA-TBA complex, a remarked tool for lipid peroxidation^55^. In this regard, the LPO assay was prepared after the treatment of the bacterial pathogens with the potent compounds, and the LPO results were plotted. A remarkable increase in the LPO was indicated in the case of **22** towards all tested bacterial pathogens as shown in (Fig. 5). The maximum formation of the MDA-TBA complex was noticed in the presence of compound **22** with the greatest value against *Staphylococcus aureus, Bacillus subtilis, Klebsiella pneumonia,* and *Pseudomonas aeruginosa* respectively. The maximum lipid peroxidation was obtained when incubating the potent compound **22** with *Staphylococcus aureus* (273.4%). Followed by, the increase of lipid peroxidation for *Bacillus subtilis* (264.8%), *Klebsiella pneumonia* (191.3%)*,* and *Pseudomonas aeruginosa* (170%) was also detected. The differences between the lipid peroxidation responses for each bacterial pathogen reflected the variations of the cell membrane contents between Gram-positive and Gram-negative bacteria and provided the efficiency of the tested compound toward each bacterial pathogen. Thus, the highest MDA–TBA complex was formed in the case of *S. aureus*, which emphasized the ability of **22** to cause significant oxidative stress against clinically important pathogens like *S. aureus* MRSA. Furthermore, the resulting MDA-TBA complex after treatment by compound **15** ensured a weak lipid peroxidation activity toward all tested pathogens except for *B. subtilis*, which increased lipid peroxidation to 157.1%. However, there was a lower LP activity against *S. aureus* (128.9%), *Klebsiella pneumonia* (108.6%) and *Pseudomonas aeruginosa* (107%) observed, which indicated the low probability of the antibacterial pathway by this mechanism was carried out.

Accordingly, our findings confirmed the potentiality of the mechanism of action of **22** as an LPO inducer in the bacterial cell membrane. The oxidative stress against each bacterial pathogen was usually accompanied by LPO activity, which plays a significant role in the peroxidation of fatty acid content in the bacterial cell membrane, leading to cell disruption. On the other hand, induced ROS by the treatment of the potent compound could be one of the mechanisms related to the oxidative stress by LPO. In addition, the hydrophobic interaction between bacterial cells and targeted compounds was also considered one of the reasons that strongly caused the lipid peroxidation of the bacterial cell membrane^56^.

### Anti-cancer activity

#### Primary screen

**Figure 6:**
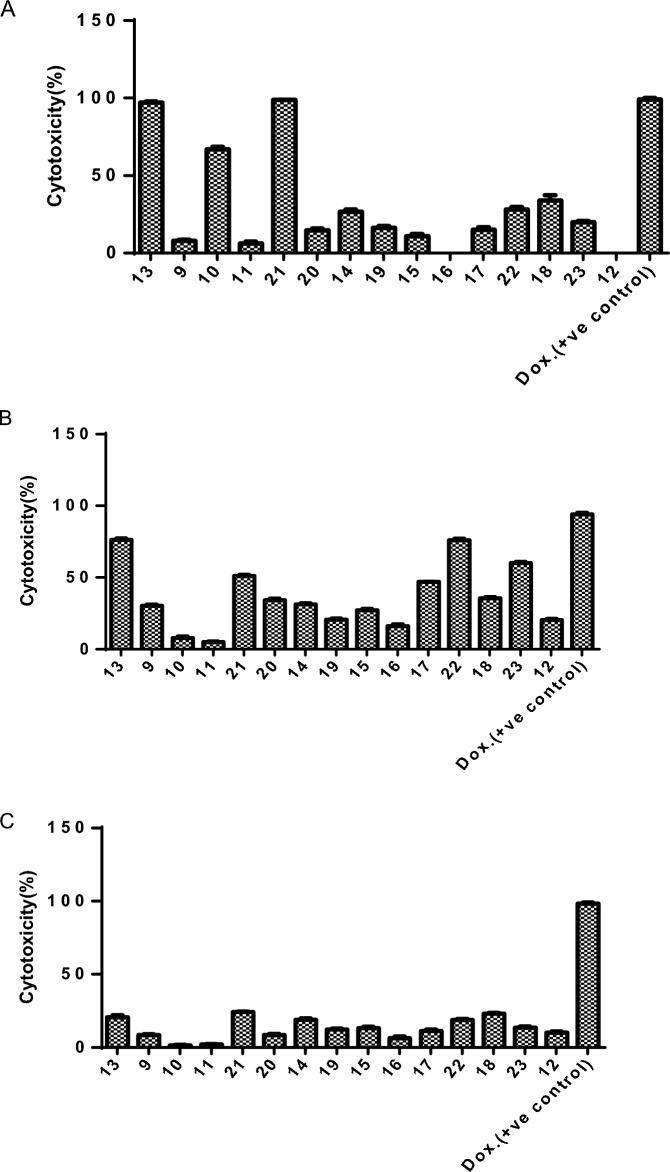
Cytotoxicity (%) of deferent concentrations of carbamide compounds **9–23** against (**A**) colon cancer cell line (HCT-116), (**B**) breast cancer cell line (MCF-7), (**C**) lung cancer cell line (A549).

**Figure 7:**
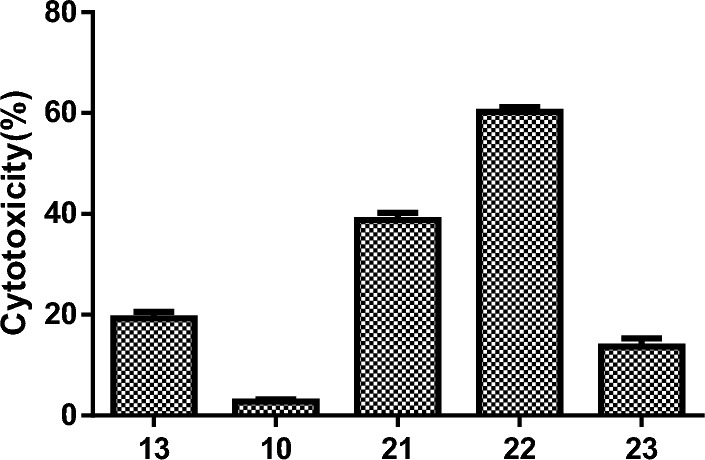
Cytotoxicity (%) of the most active compounds **10, 13, 21, 22** and **23** against human normal skin fibroblast cells (BJ-1).

The cytotoxic activity of the synthesized compounds **9–23** have been evaluated on different cancer cell lines namely human breast cancer (MCF-7), human colon cancer (HCT116), and human non-small cell lung cancer (A549), as well as the normal skin fibroblast cell (BJ-1) through in vitro MTT assay. The initial screening assay was at a single dose concentration of (100 µM) for 48 h. The cytotoxicity of the treated cells was determined as cell death % compared to untreated cells (Fig. 6A–C). The results refereed that, three compounds (**10**, **13**, and **21**) showed a potent cytotoxic effect (≥ 65%) against colon cancer (HCT-116) with IC_50_ values (IC_50_ = 72.6, 43.5, 38.5 and 43.6 µM, respectively) (Fig. 6A, Table 5). As shown in Fig. 6B, four compounds (**13**, **21**, **22** and **23**) showed a potent cytotoxic effect (≥ 51%) against breast cancer (MCF-7) with IC_50_ values (62.4, 91.6, 57.9 and 68.6 µM, respectively) (Table 5). According to the above results, compound **13** is the most active compound with a highly cytotoxic effect against both cancer cells. As illustrated in Fig. 6C, all the tested compounds showed low cytotoxic effects against lung cancer cells ranging from 1.6 to 24.6 cytotoxicity (%). Additionally, the most active compounds that showed cytotoxic effects against at least cancer cells were subjected to investigate the cytotoxicity on normal cells (Bj-1). As shown in Fig. 7, these compounds (**10**, **13**, **21**, **22** and **23**) were shown to be safe for normal cells up to a concentration of 100 µM (cytotoxicity % = 19.7%, 2.7%, 38.9% and 13.7%, respectively). On the other hand, compounds **21** and **22** possessed moderate cytotoxic effects on the normal cells at the same concentration (cytotoxicity (%) = 47.2% and 60.2%, respectively). Based on these findings, we further studied the cellular mechanisms underlying the potent cytotoxic effect of the most active compounds against colon and breast cancer cells.

**Table 5:** IC_50_ (µM) of the most active compounds **10**, **13** and **21–23** on HCT-116 and MCF-7.

Compound	IC_50_	
	HCT-116	MCF-7
**10**	72.6 ± 0.13	**–**
**13**	43.5 ± 0.15	62.4 ± 0.128
**21**	38.5 ± 0.17	91.6 ± 0.112
**22**	**–**	57.9 ± 0.122
**23**	**–**	68.6 ± 0.116
Doxorubicin	65.1 ± 0.141	45.0 ± 0.123

#### Cell cycle arrest

**Figure 8:**
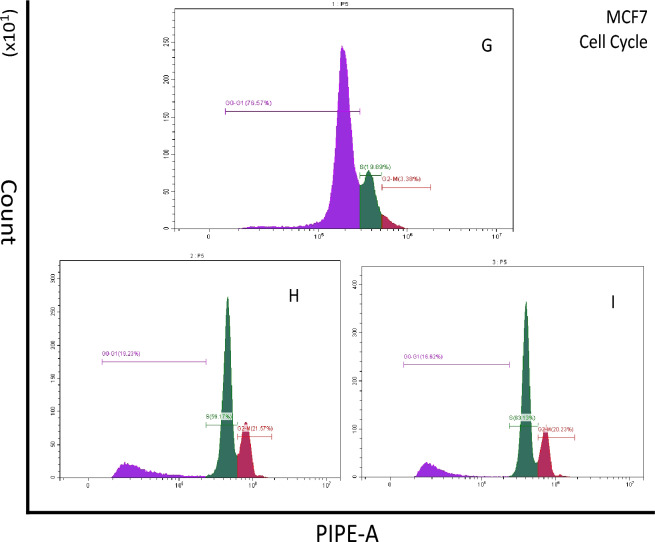
MCF-7 cell-cycle analysis by flow cytometry: (**G**) control group, (**H**) **13** treated cells, (**I**) **22** treated cells.

**Figure 9:**
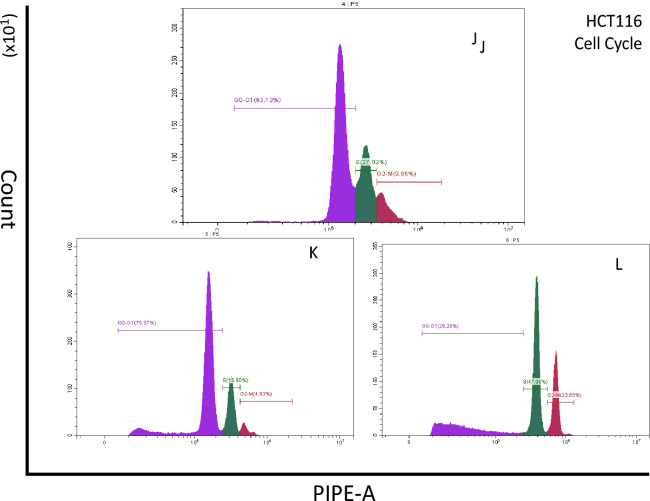
HCT-116 cell-cycle analysis by flow cytometry: (**J**) control group, (K) **21** treated cells group, (**L**) **13** treated cells group.

In this study, cancer cells (MCF-7 and HCT-116) were treated with different compounds (**13** and **22** for MCF-7, and **13** and **21** for HCT-116). The treated cells were then stained with PI and analyzed using flow cytometry to visualize the proportion of DNA content. Additionally, cells that were not exposed to any treatment were used as controls. The findings revealed that compounds **13** and **22** led to cell cycle arrest at the S & G2/M phases in MCF-7 cells, while compound **13** induced S & G2/M cell cycle arrest in HCT-116 cells. However, the effects of compound **21** on HCT-116 cells were weaker than those of compound **13**. All the results were summarized in Figs. 8 and 9.

#### Necrosis cell apoptosis assay

**Figure 10:**
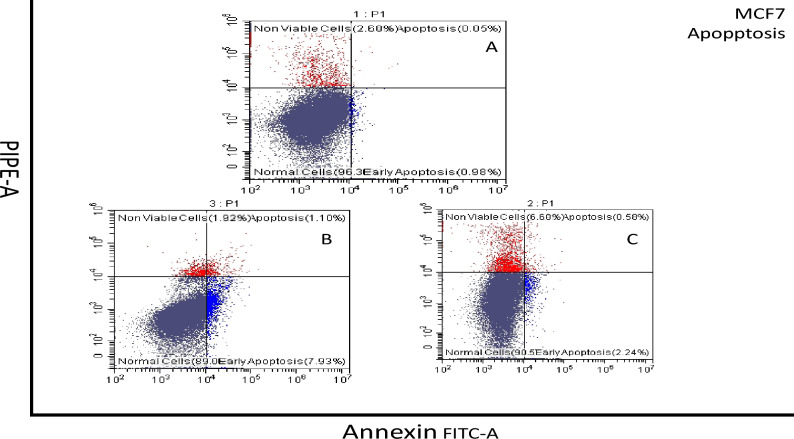
Flow cytometry profiles represent Annexin V-FITC staining on the X-axis and PI on the Y-axis. Annexin V-FITC Apoptosis assay results showing the percentages of MCF-7 cell death modes: necrosis, late apoptosis, early apoptosis, and live cell: (**A**) control group, (**B**) **22**-treated cells group, and (**C**) **13-**treated cells group.

**Figure 11:**
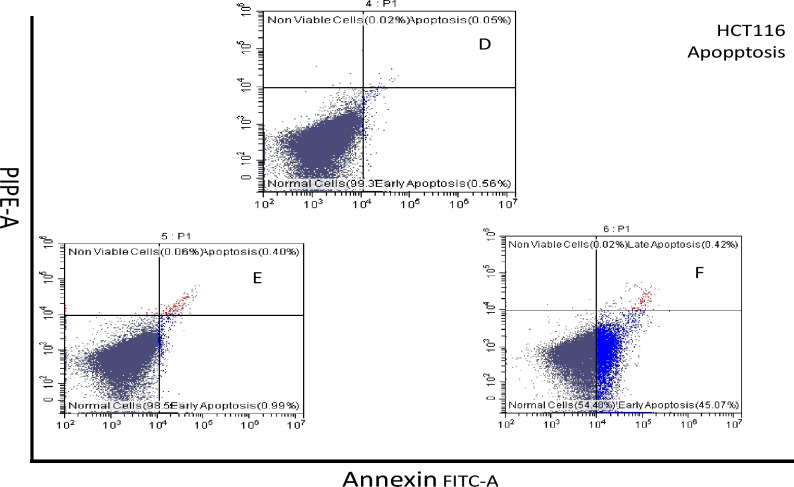
Flow cytometry profiles represent Annexin-V-FITC staining on the X-axis and PI on the Y-axis. Annexin V-FITC Apoptosis assay results showing the percentages of HCT-116 cell death modes: necrosis, late apoptosis, early apoptosis, and live cell: (**D**) control, (**E**) **21**-treated cells group, and (**F**) **13**-treated cells group.

The impact of compound **13** on MCF-7 cell samples resulted in 2.24% of the population being in early apoptosis, 0.58% in late apoptosis, and 6.6% in the necrotic population. In contrast, compound **22** caused 7.93% of the population to be in early apoptosis, 1.1% in late apoptosis, and 1.9% necrotic population. Similarly, compound **13** on HCT-116 cell samples led to 45.07% of the population being in early apoptosis, 0.42% in late apoptosis, and 0.02% necrotic population. However, compound **21** resulted in only 0.99% in early apoptosis, 0.44% in late apoptosis, and 0.06% necrotic population. These findings are depicted in Figs. 10 and 11.

Cancer is the second leading cause of death in developed nations, responsible for approximately one out of every five deaths^11^. Disrupting the dysregulated cell cycle progression in cancer cells is closely associated with the suppression of cell proliferation and apoptosis^57^. Therefore, targeting the cell cycle has emerged as a promising therapeutic strategy for cancer treatment. The design and synthesis of carbamide derivatives bearing different aliphatic amine moieties have been a subject of consistent interest as this promises to have anti-proliferative and apoptotic-inducing activity^58–60^. In the present study, compounds **13** and **22** showed antiproliferative effects on cancer cells, which are consistent with previous reports. Pirol 2014 and Cao 2013 both report the synthesis of novel derivatives of 4-methyl-*N*-(4-tolyl)piperazine-1-carboxamide and their antiproliferative activities against various human cancer cell lines^58,59^.

When the chemical structure of the compounds is taken into consideration, the prepared 4-methyl-N-(p-tolyl)piperazine-1-carboxamide in our study has enhanced the ability to induce cell cycle arrest in cancerous cell lines with the best results against MCF7 and HCT116. Additionally, the incorporation of amine groups into carbamide may offer a way to modify the biological activity of carbamide derivatives leading to a significant enhancement in their ability to induce apoptosis^61^. This enhancement was evident in our study of compound **13** on HCT 116 as 45.07% of the cell population exhibited early apoptosis, 0.42% displayed late apoptosis, and 0.02% showed signs of necrosis. These effects may be attributed to different mechanisms according to molecular docking analysis. Some of the possible mechanisms include degrading CDK, inhibiting EGFR activity, degrading ERα, decreasing ERα signaling, and decreasing VEGFR signaling.

## Molecular docking study

### Molecular docking of selected molecules with antimicrobial receptor proteins

**Figure 12:**
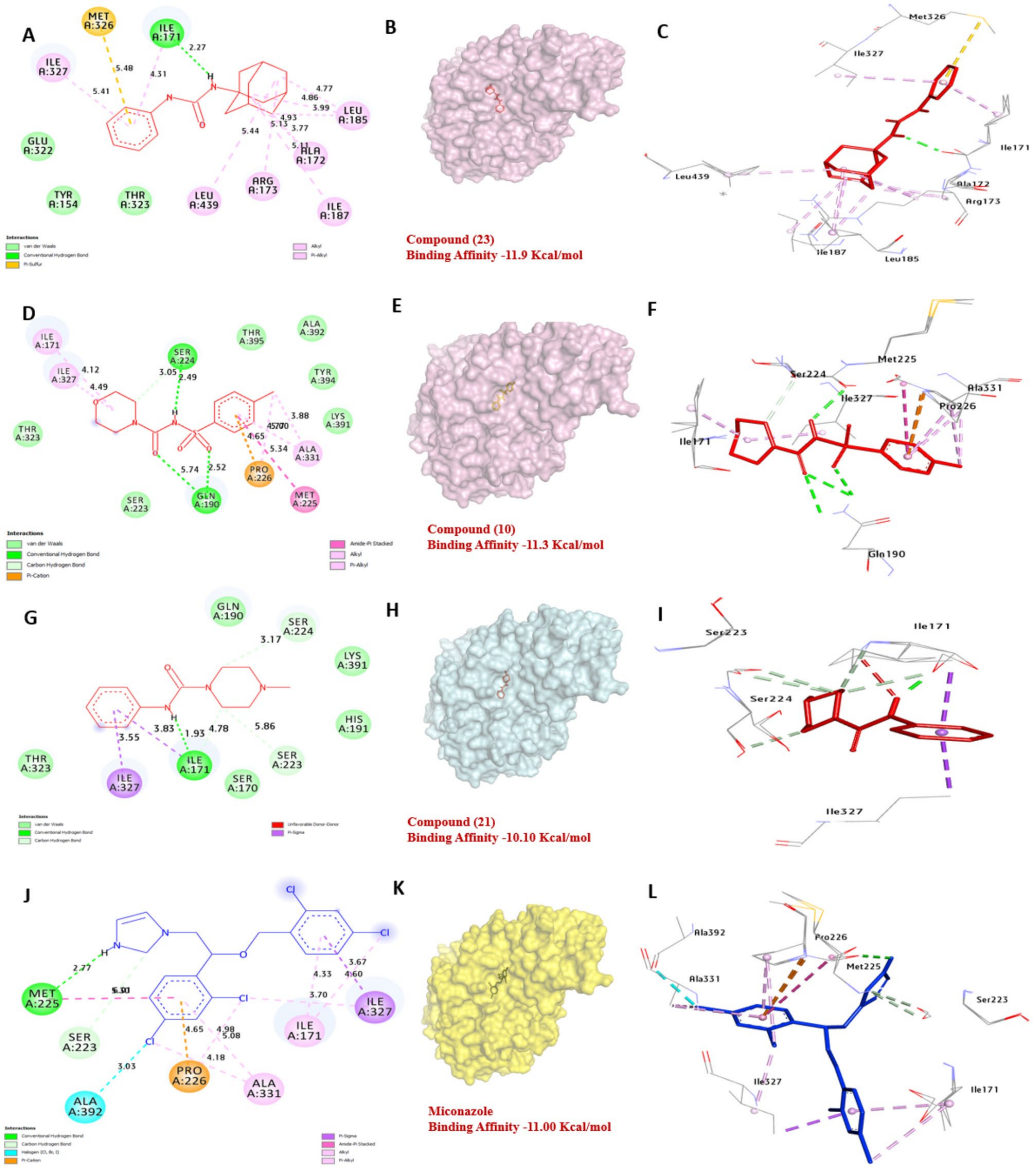
3D representations of compounds **10**, **21** and **23** conformation at the binding pocket of FDC1 protein of *A. fumagitus* (PDB: ID 4ZA5). (**A**, **B** and **C**) **23**, (**D**, **E** and **F**) **10**, (**G**, **H**, and **I**) **21**, (**J**, **K**, and **L**) **Miconazole**.

Feruloyl-CoA synthetase (FDC1) in *A. fumigates* is involved in the degradation of lignocellulose. Our docking, of FDC1 protein, has a strong affinity for compounds **10**, **21** and **23** with binding energies of − 11.30, − 10.10, and − 11.90, kcal/mol compared with (Miconazole − 11.00 kcal/mol). Compound **10** formed three hydrogen bonds with Ser224 and Gln190 also, hydrophobic interactions with Ala331 (Pi-alkyl), Ile171 (Pi-alkyl), Ile327 (Pi-alkyl), Met225 (Pi-T-shaped), Pro226 (Pi-cation), and Ser224 (carbon-Bond). Also, both compounds **21** and **23** formed one hydrogen bond with Ile171 and hydrophobic interactions with Ile327 (Pi-sigma), Ile171 (Pi-sigma), Ser223 (carbon-bond), and Ser224 (carbon-bond. The residues Ile171, Met225, and Ser224 in the catalytic site were found to have a positive effect on the binding affinity of the compounds. Likewise, Compound **23** formed hydrophobic interactions with Ala172 (Pi-alkyl), Arg173 (Pi-alkyl), Leu185 (Pi-alkyl), Leu439 (Pi-alkyl), Met326 (Pi-Sulfur), Ile327 (Pi-alkyl. Overall, the results suggest that compounds **10**, **21** and **23** are the most promising compounds as potential inhibitors of FDC1 protein (Fig. 12) and (Table S1, see supplementary information).

**Figure 13:**
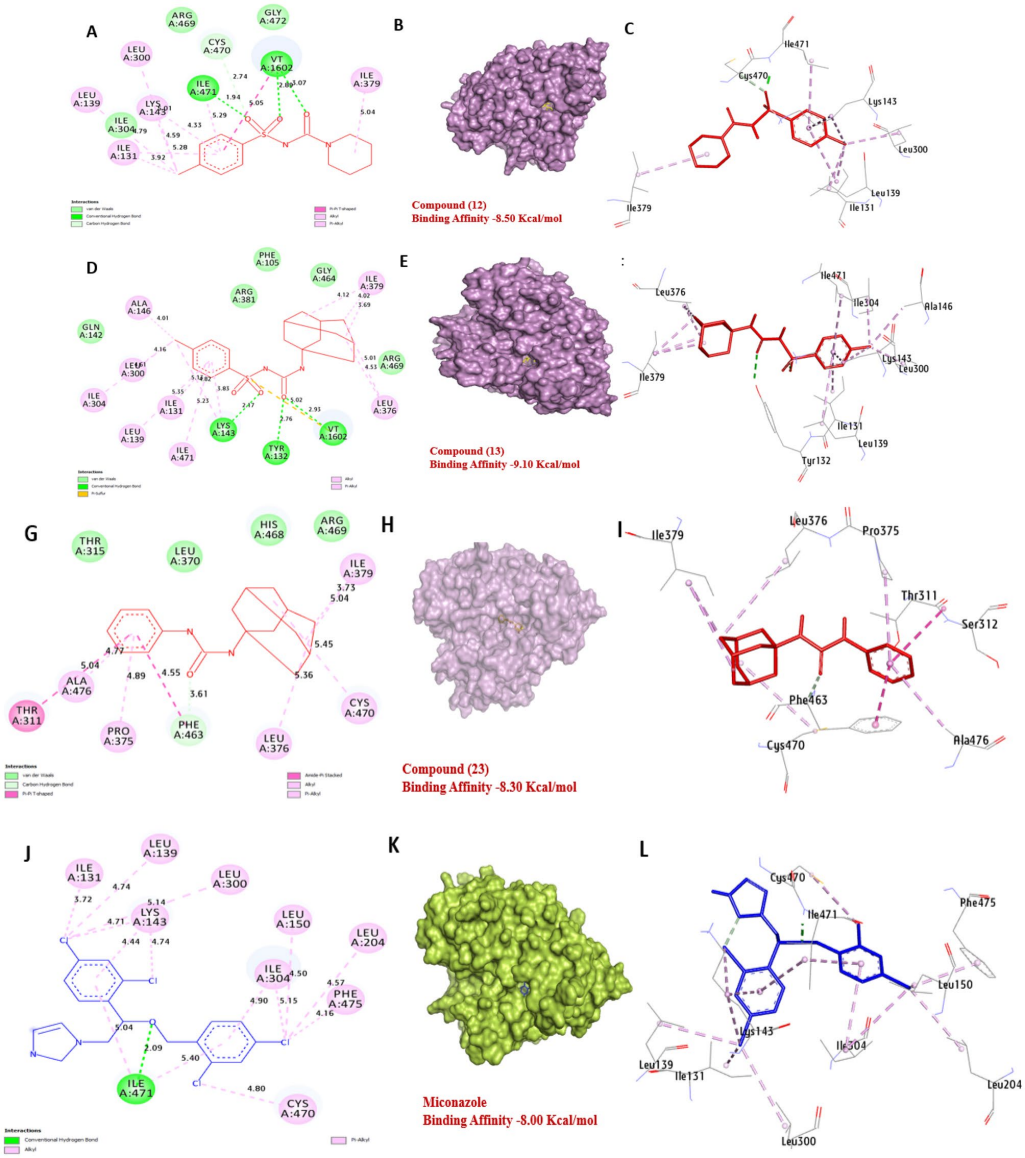
3D representations of compounds **12**, **13** and **23** conformation at the binding pocket of the Sterol 14-demethylase of *C. albicans* (PDB: ID 5TZ1). (**A**, **B** and **C**) **12**, (**D**, **E** and **F**) **13**, (**G**, **H**, and **I**) **23**, (**J**, **K**, and **L**) Miconazole.

Sterol 14-demethylase of *C. albicans* is an enzyme that plays a critical role in the biosynthesis of the cell membrane. It has a strong affinity for compounds **12**, **13** and **23** with binding energies of − 8.50, − 9.10 and − 8.30 kcal/mol compared with (Miconazole − 8.00 kcal/mol). Compound **12** formed one hydrogen bonds with Ile471 and hydrophobic interactions with Ile131 (Pi-alkyl), Leu139 (Pi-alkyl), Lys143 (Pi-alkyl), Ile300 (Pi-alkyl), Cys470 (C-Hydrogen bond), Ile379 (Pi-alkyl). Also, compound **13**, formed two hydrogen bonds with Lys143 and Tyr132, also, hydrophobic interactions with Ala146 (Pi-alkyl), Ile379 (Pi-alkyl), Leu139 (Pi-alkyl), Ile131 (Pi-alkyl), Ile471 (Pi-alkyl), Ile304 (Pi-alkyl), Leu376 (Pi-alkyl), Leu300 (Pi-alkyl). Compound **23** formed hydrophobic interactions with Cys470 (Pi-sulfur), Ile379 (Pi-alkyl), Thr311 (Pi-sigma), Ala476 (Pi-alkyl), Pro375 (Pi-alkyl), Leu376 (Pi-alkyl), and Phe463 (C-Hydrogen). The residues Ile471, Lys143, and Tyr132 were found to have a positive effect on the binding affinity of the compounds. Overall, the results suggest that compounds **12**, **13** and **23** are the most promising as potential inhibitors of Sterol 14-demethylase in *C. albicans* (Fig. 13) and (Table S2, see supplementary information).

**Figure 14:**
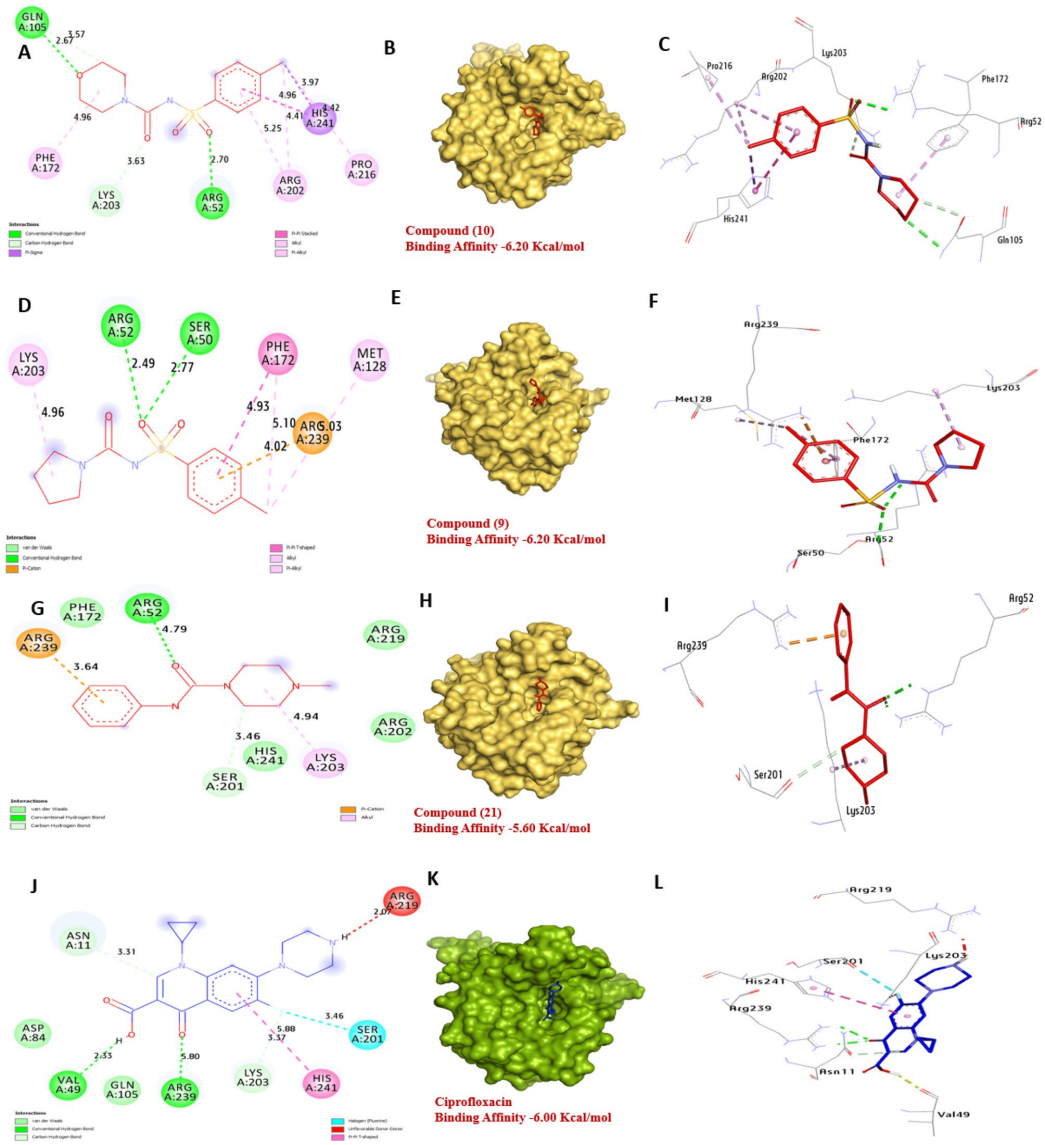
3D representations of compounds **10** and **21** at the binding pocket of Dihydropteroate synthase of *S.aureus* (PDB: ID 1AD4). (**A**, **B** and **C**) **10**, (**D**, **E** and **F**) **9**, (**G**, **H**, and **I**) **21**, (**J**, **K**, and **L**) Ciprofloxacin.

Dihydropteroate synthase in *S. aureus* is an enzyme involved in folate biosynthesis. Inhibition of this enzyme can lead to DNA damage and cell death in bacteria. Dihydropteroate synthase has a strong affinity for the compounds **9**, **10** and **21** with binding energies of − 6.20, − 6.20, and − 5.60 kcal/mol, respectively, compared with Ciprofloxacin (− 6.00 kcal/mol). Compounds **10** formed two hydrogen bonds with Arg52 and Gln105, also, hydrophobic interactions with Arg202 (Pi- alkyl), His241 (Pi-sigma), His241 (Pi-Pi stacked), Pro216 (Pi-alkyl), Phe172 (Pi-alkyl), Lys203 (Carbon H- Bond). Additionally, compound **9** formed two hydrogen bonds with Arg52 and Ser50, also, hydrophobic interactions with Phe172 (Pi-alkyl), Phe172 (Pi-Pi stacked), Lys203 (Pi-alkyl), Met128 (Pi-alkyl), and Arg239 (Pi-cation). Finally, compound **21** formed one hydrogen bond with Arg52, also, hydrophobic interactions with Arg239 (Pi-cation), Lys203 (Pi-alkyl), and Ser201 (Carbon H-Bond). The residues Arg52, Gln105, Arg239, and Ser50 in the catalytic site were found to positively influence the binding affinity (Fig. 14) and (Table S3, see supplementary information).

**Figure 15:**
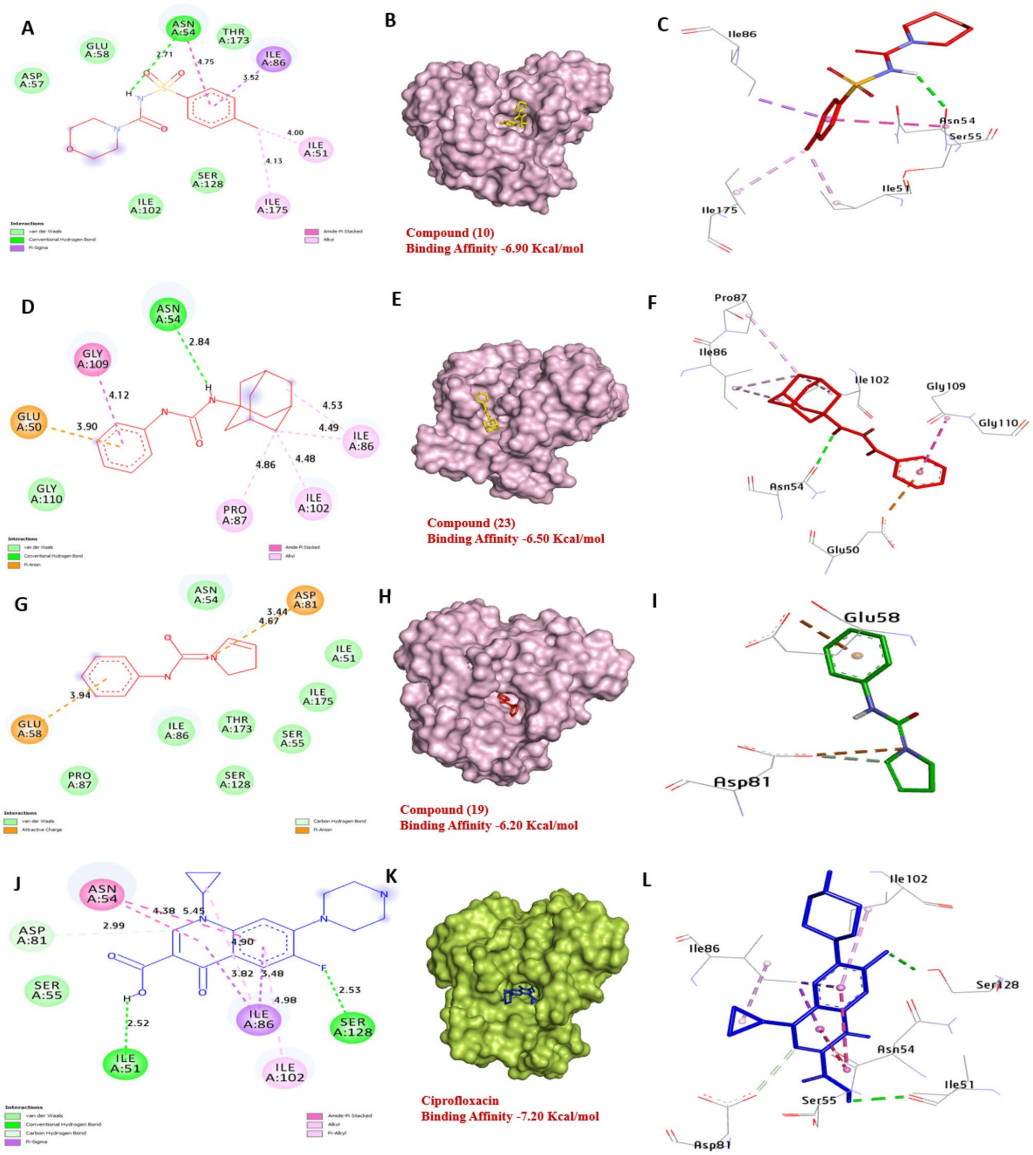
3D representations of compounds **10**, **19** and **23** conformations at the binding pocket of Gyrase B proteins of *B. subtilis* (PDB: ID 4URM) (**A**, **B** and **C**) **10**, (**D**, **E** and **F**) **23**, (**G**, **H**, and **I**) **19**, (**J**, **K**, and **L**) Ciprofloxacin.

Gyrase B in *B. subtilis* is a type II topoisomerase enzyme, it plays a critical role in DNA replication, recombination, and repair. Therefore, according to docking results, it appears that compounds **10** and **23** have the greatest affinity interaction − 6.90, and − 6.50 kcal/mol, respectively compared with Ciprofloxacin (− 7.20 kcal/mol) with Gyrase B in *B. subtilis*, forming hydrogen bonds with Asn54, along with hydrophobic interactions with Ile51 (Pi-alkyl), Ile175 (Pi-alkyl), Asn54 (Amid-pi-shaped), Ile86 (Pi-sigma), Gly109 ( Pi- cation), Pro87 (Pi-alkyl), Glu50 (Amid-pi-shaped), Ile102 (Pi- alkyl), and Ile86 (Pi- alkyl. the amino acids Asn54, Ser128, and Ile51 in the catalytic site appear to enhance the binding affinity. These findings suggest that compounds **10**, **19** and **23** have potential as inhibitors of Gyrase B activity (Fig. 15) and (Table S4, see supplementary information).

**Figure 16:**
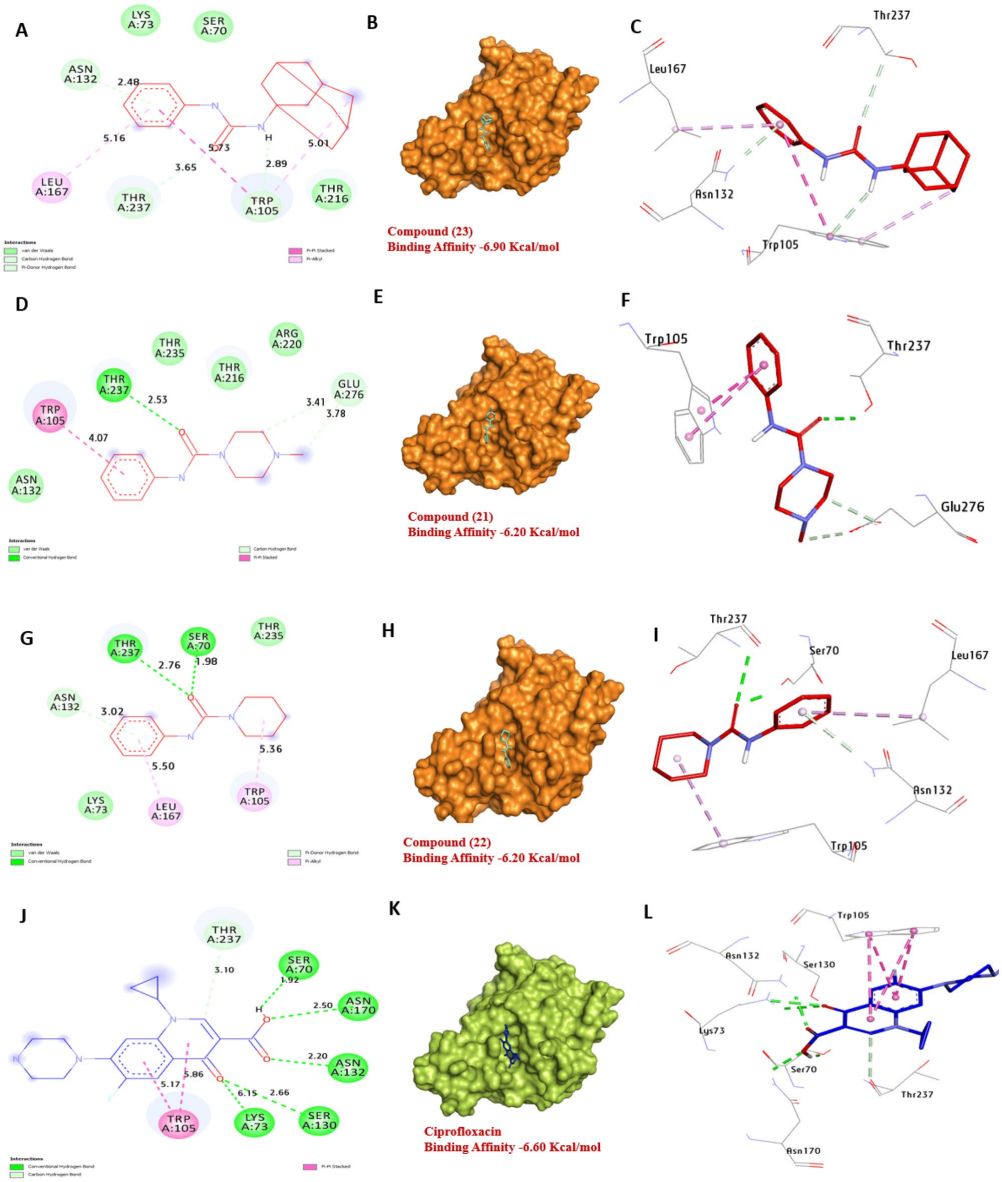
3D representations of compounds **21**, **22** and **23** conformations at the binding pocket of Glucosamine-6-phosphate synthase in *K. pneumenia* (PDB: ID 2VF5). (**A**, **B** and **C**) **23**, (**D**, **E** and **F**) **21**, (**G**, **H**, and **I**) **22**, (**J**, **K**, and **L**) Ciprofloxacin.

Glucosamine-6-phosphate synthase is an essential enzyme in *k. pneumonia*, making it a potential target for the development of antibiotics. Based on the docking results, compounds **21**, **22** and **23** have a strong affinity with a binding energy of − 6.20, − 6.20 and − 6.90, kcal/mol, compared with Ciprofloxacin (− 6.60 kcal/mol). Compounds **21** and **22** formed four hydrogen bonds with Thr237 and Ser70, and hydrophobic interactions formed with three compounds **21**, **22** and **23** with several other residues, such as (Pi-alkyl) with Leu167, and Trp105, (Carbon-H) with Thr237, Trp105, Asn132, Glu276 and Asn132, (Pi- stacked) with Trp105. The amino acids Thr237, Ser70, and Asn132 in the catalytic site were found to enhance the binding affinity of the compound. Overall, these findings suggest that compounds **21**, **22** and **23** have potential as an inhibitor of Glucosamine-6-phosphate synthase in *k. pneumonia* (Fig. 16) and (Table S5, see supplementary information).

**Figure 17:**
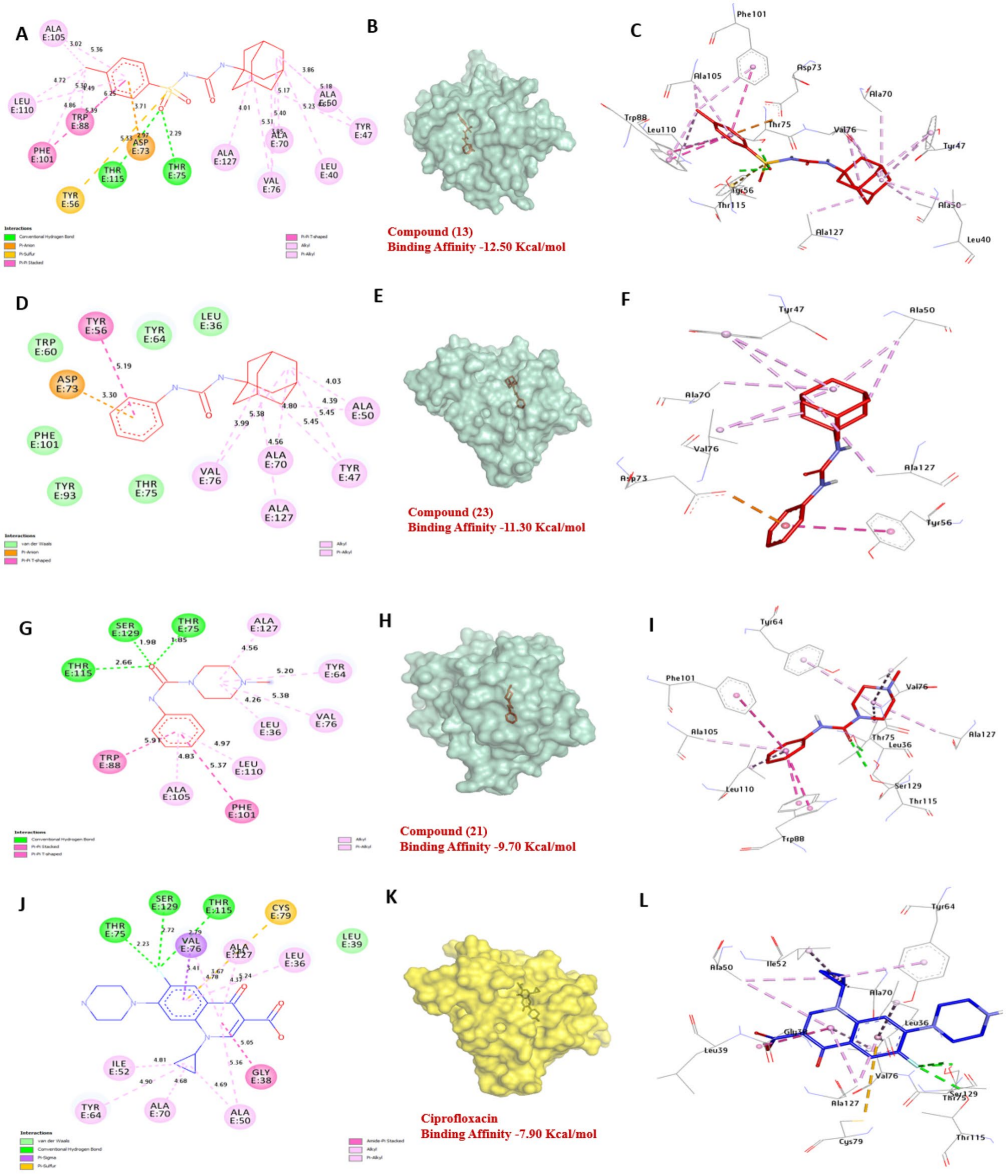
3D representations of compounds **13**, **21** and **23** conformations at the binding pocket of Sortase A protein of *E. coli* (PDB: ID 2UV0). (**A**, **B** and **C**) **13**, (**D**, **E** and **F**) **23**, (**G**, **H**, and **I**) **21**, (**J**, **K**, and **L**) Ciprofloxacin.

According to the docking findings for LasR an activator of exotoxin of *P. aeruginosa*, Compounds **13**, **21** and **23** show a greater affinity of − 12.50, − 9.70 and 11.30 kcal/mol, respectively compared to Ciprofloxacin (− 7.90 kcal/mol). Compounds **13** and **21** formed four hydrogen bonds with Thr75, Thr115, and Ser129, and hydrophobic interactions formed with three compounds **13**, **21** and **23** such as (Pi-alkyl) with Ala105, Ala127, Val76, Leu110, Leu40, Tyr47, Leu36, Ala50, Ala70, and Val76, (Pi-Pi-T-shaped) with Phe101, Tyr56, and Trp88, (Pi-Pi-Stacked) with Trp88 and Phe101, (Pi-Anion) with Asp73, (Pi-sulfur) with Tyr56. The amino acids Thr75, Thr115, and Ser129 in the catalytic site were found to enhance the binding affinity of the compound. Overall, these findings suggest that compounds **13**, **21**, and **23** have potential as an inhibitor of LasR. The in-vitro antibacterial activity results support the findings, indicating that compounds **13**, **21** and **23** may be effective as bacterial inhibitors (Fig. 17) and (Table S6, see supplementary information).

### Molecular docking of selected compounds with anticancer receptor proteins

**Figure 18:**
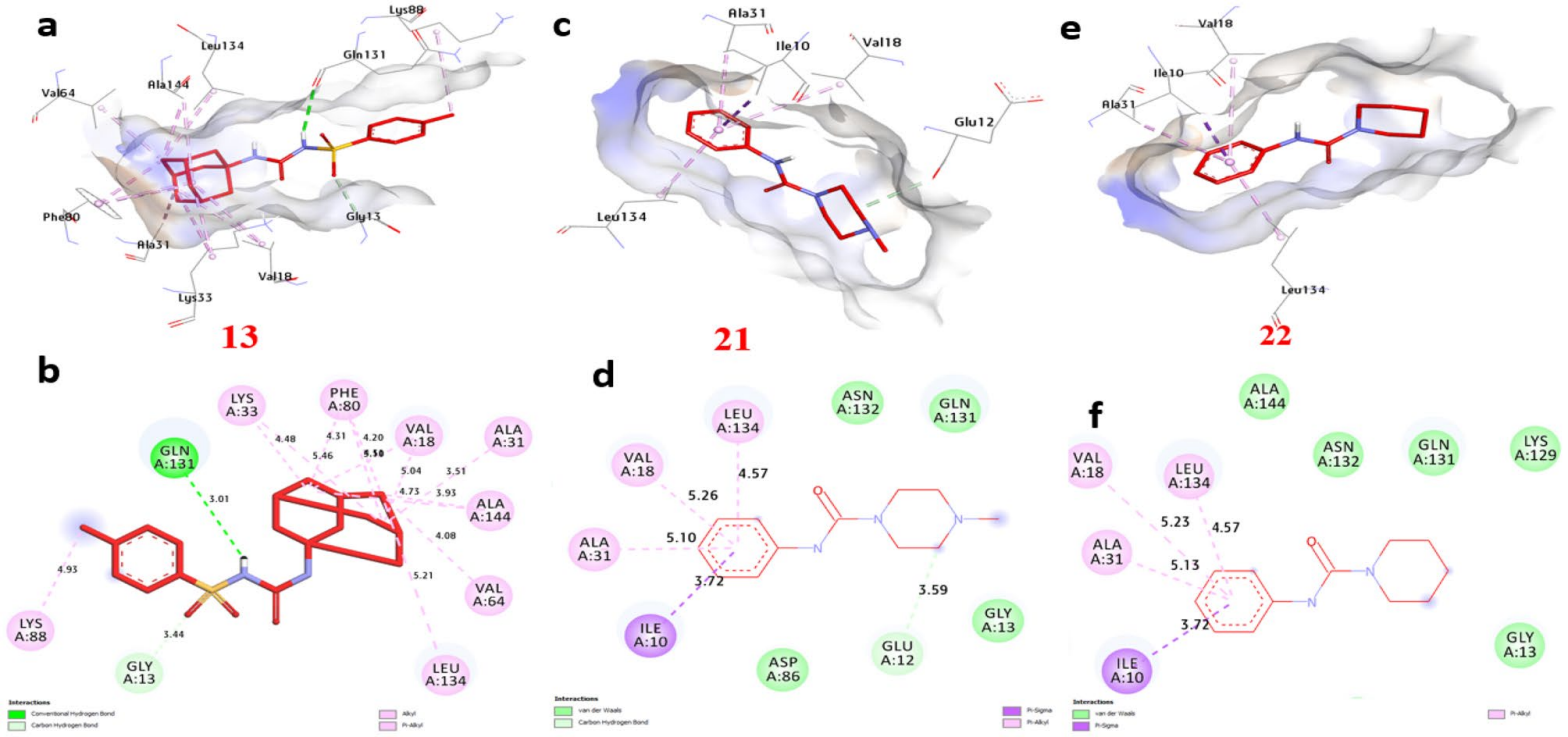
3D representations of compound conformations at the binding pocket of CDK2 (PDB: ID 2A4L) (**a** and **b**) **13**, (**c** and **d**) **21**, (**e **and **f**) **22**.

**Figure 19:**
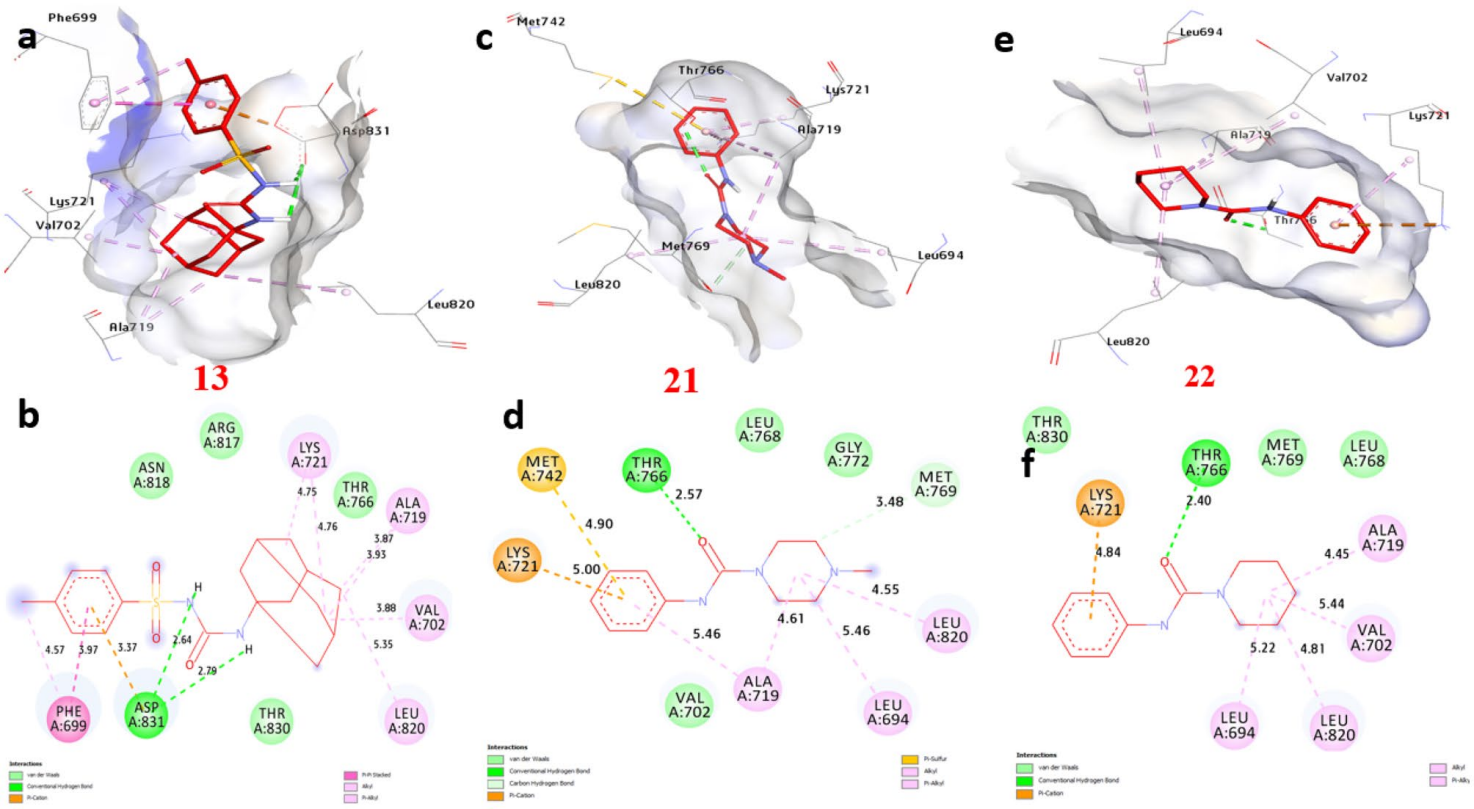
3D representations of compound conformations at the binding pocket of the EGFR (PDB: ID 1M17) (**a** and **b**) **13**, (**c** and **d**) **21**, (**e **and **f**) **22**.

**Figure 20:**
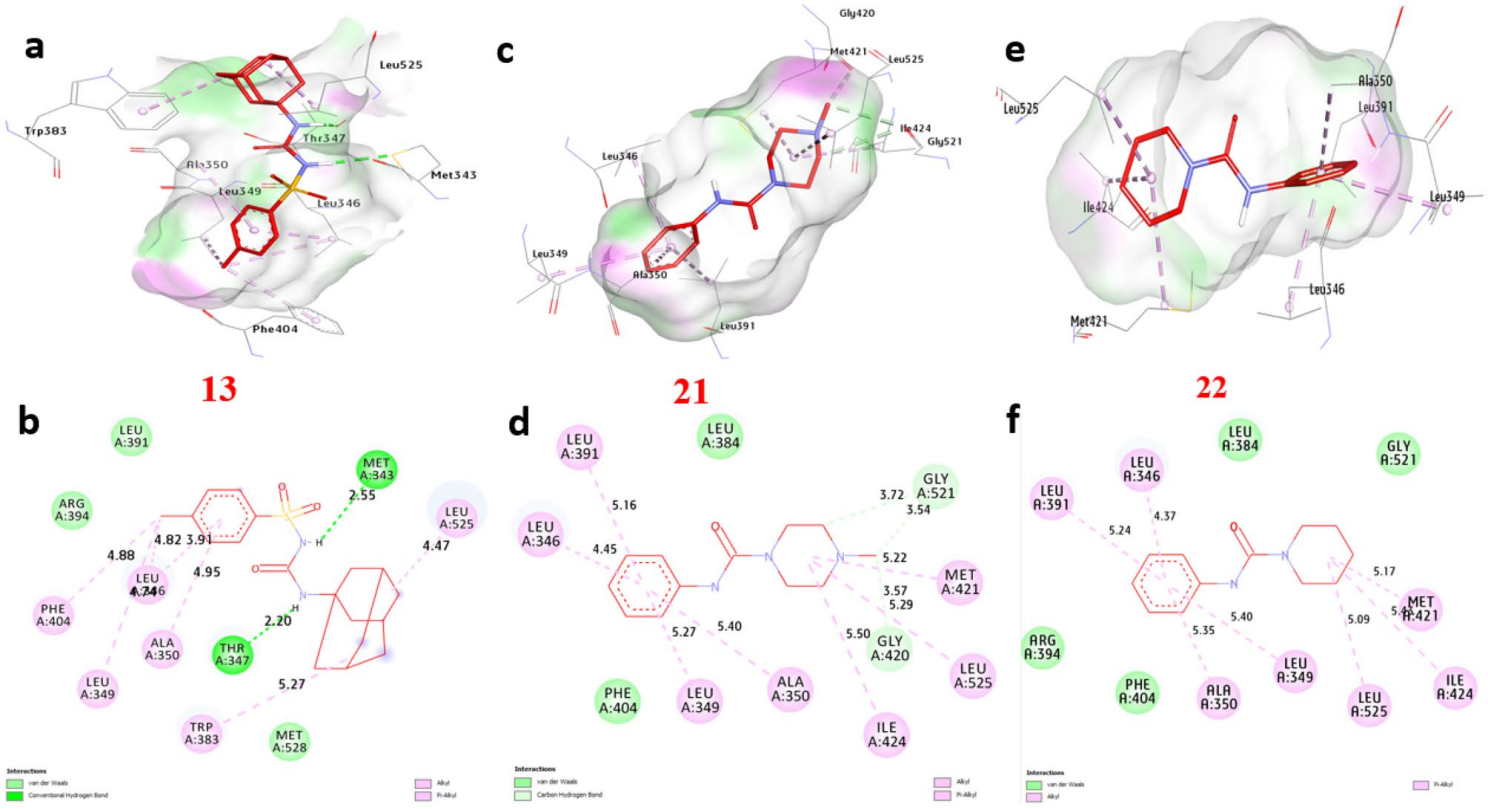
3D representations of compound conformations at the binding pocket of ERalfa (PDB: ID 3ERT) (**a** and **b**) **13**, (c and d) **21**, (**e** and **f**) **22**.

**Figure 21:**
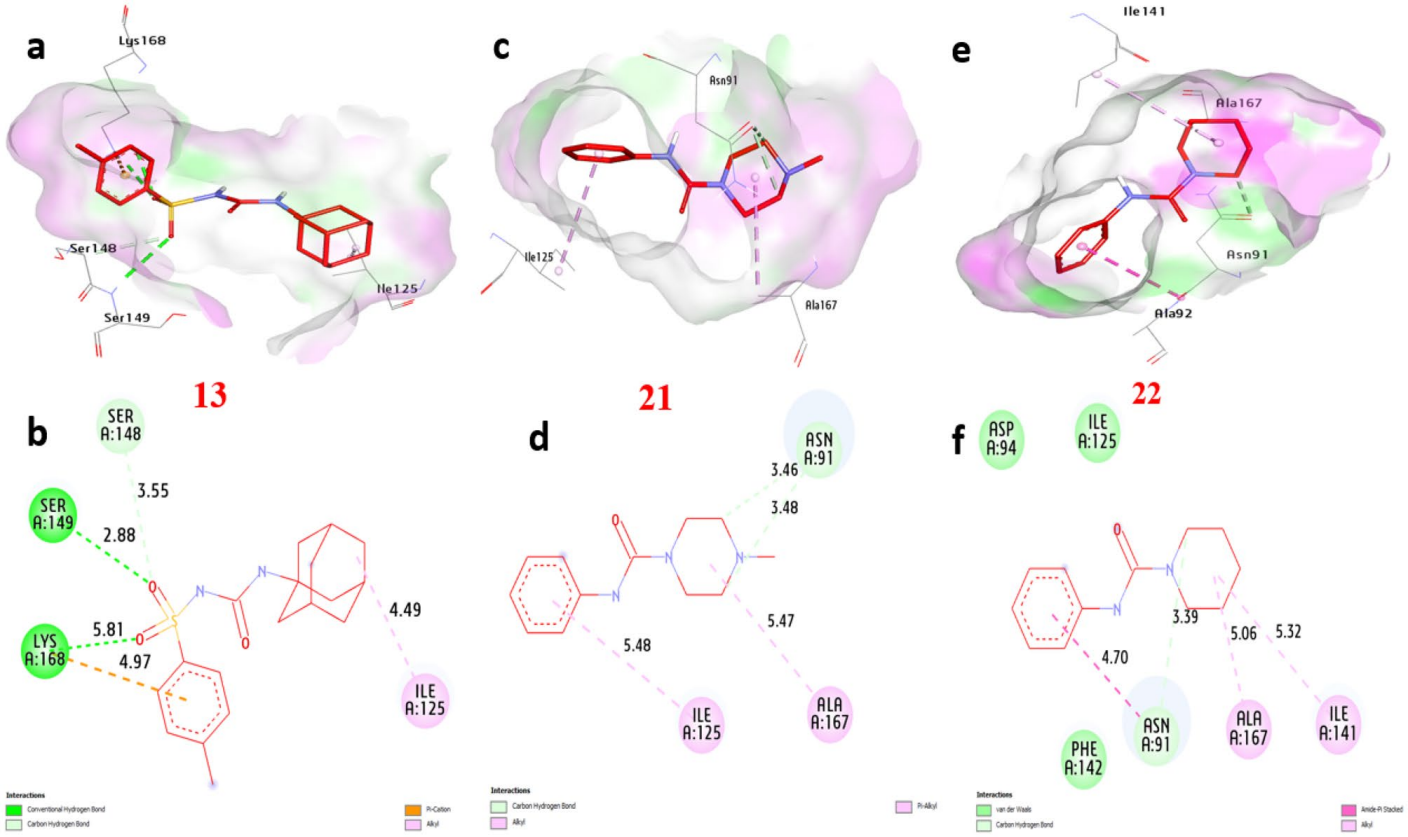
3D representations of compound conformations at the binding pocket of Topoisomerase11 (PDB: ID 1ZXM) (**a** and **b**) **13**, (**c** and **d**) **21**, (**e **and **f**) **22**.

**Figure 22:**
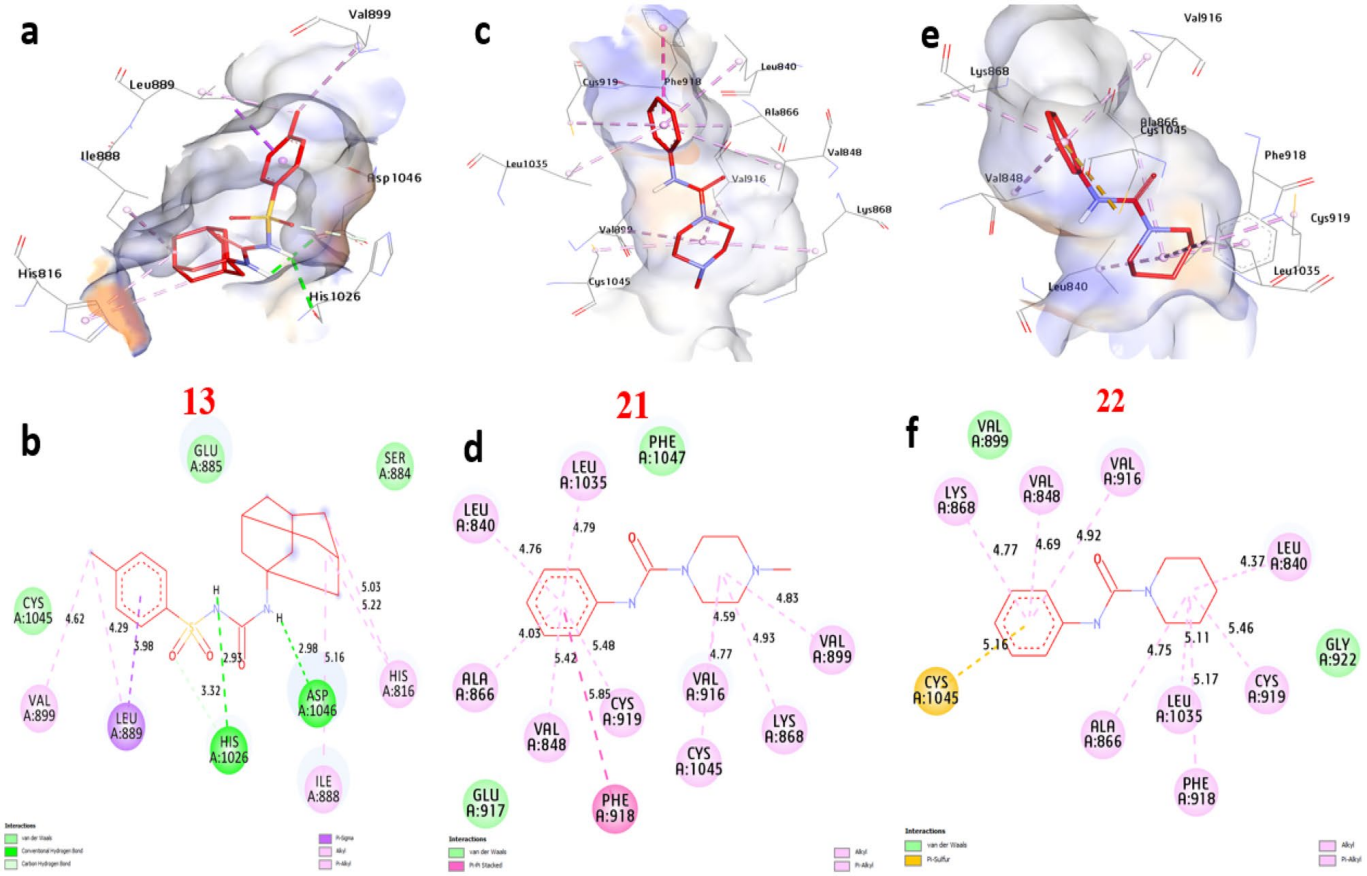
3D representations of compound conformations at the binding pocket of VEGFFR (PDB: ID 3WZE). (**a** and **b**) **13**, (**c** and **d**) **21**, (**e** and **f**) **22**.

Firstly, CDK2 is an enzyme that plays a crucial role in cell cycle regulation and progression. Docking of CDK2 revealed that **13**, **21** and **22** showed the strongest affinity interaction with binding energies of − 8.60, − 8.60 and − 8.00 kcal/mol, respectively compared with doxorubicin (− 7.90 kcal/mol) (Figure S2, see supplementary information). Compound **13** formed one hydrogen bond with Gln131. Also, non-hydrophilic bonds were observed with compounds **13**, **21** and **22** including (alkyl bond) with Val18, Phe80, Lys33, Leu134, and Ala31, (Carbon-Hydrogen Bond) with Gly13 and Glu12, (Pi-sigma) with Ile10 (Fig. 18) and (Table S7, see supplementary information). Secondly, EGFR (Epidermal Growth Factor Receptor) is a receptor protein involved in cell signaling and plays a significant role in cell growth, proliferation, and survival. Our docking analysis of compounds **13**, **21** and **22** showed the strongest affinity interaction with binding energies of − 8.70, − 8.10 and − 8.30 kcal/mol, respectively compared with doxorubicin (− 8.50 kcal/mol). Compounds **13**, **21** and **22** formed hydrogen bonds with Asp831 and Thr766. Also, non-hydrophilic bonds were observed with compounds **13**, **21** and **22** including (alkyl bond) with Phe699, Leu820, Ala719, Lys721, Leu694, and Val702, (Carbon-Hydrogen Bond) with Met769, (Pi-sulfur) with Met742, (Pi-cation) with Lys721 and Asp831, (Pi-Pi-stacked) with Phe699. The residues Gln196, Arg231, and Gly280 in the catalytic site were found to enhance the binding affinity (Fig. 19) and (Table S7, see supplementary information). Thirdly, ERα (Estrogen Receptor alpha) plays a crucial role in the development and differentiation of breast tissue and bone metabolism. Docking results of compounds **13**, **21** and **22** showed the strongest affinity with binding energies of − 8.80, − 7.50 and − 9.20 kcal/mol, respectively compared with doxorubicin (− 8.10 kcal/mol). Compound **13** formed two hydrogen bonds with Thr347 and Met343. Also, non-hydrophilic bonds were performed with compounds **13**, **21** and **22** including (alkyl bonds) with, Trp383, Leu746, Leu525, Phe404, Leu349, Ala350, Met421, Ile424, Leu391 and Leu346 (Carbon-Hydrogen Bond) with Gly521, and Gly420 (Fig. 20) and (Table S7, see supplementary information). Fourthly, Topoisomerase II is essential for maintaining the integrity and proper functioning of the genome. Docking revealed that compounds **13**, **21** and **22** showed the strongest affinity with binding energies of − 8.90, − 7.20 and − 7.90 kcal/mol, respectively compared with doxorubicin (− 7.00 kcal/mol). Compound **13** formed two hydrogen bonds with Ser149, and Lys168. Also, non-hydrophilic bonds were performed with compounds **13**, **21** and **22** including (alkyl bonds) with, Ile125, Ala167, and Ile141, (Carbon-Hydrogen bonds) with Ser148, and Asn91. (Pi-cation) with Lys168, (Pi-Pi- stacked) with Asn91. The residues Ile125, Ala167, and Lys168 were found to enhance the binding affinity (Fig. 21) and (Table S7, see supplementary information). Finally, VEGFFRs are a family of receptors that play a significant role in angiogenesis and are targets for therapeutic interventions. Docking of VEGFFR revealed that compounds **13**, **21** and **22** showed the strongest affinity interaction with VEGFFR, with calculated binding energies of − 8.90, − 8.50 and − 8.80 kcal/mol, respectively compared with doxorubicin (− 7.90 kcal/mol). Compound **13** formed hydrogen bonds with His1026 and Asp1046. Also, non-hydrophilic bonds were performed with compounds **13**, **21** and **22** including (alkyl bonds) with, His816, Ile888, Leu889, Val899, Lys868, Cys1045, Val916, Leu1035, Ala866, Val848, Cys919, Leu840, and Phe918, (Carbon-Hydrogen Bond) with His1026, (Pi-sulfur) with Cys1045, (Pi-sigma) with Leu840 and Leu889, (Pi-Pi-stacked) with Phe918. The residues His1026, Lys868, and Val848 in the binding site were found to enhance the binding affinity (Fig. 22) and (Table S7, see supplementary information).

### In silico pharmacokinetics ADME prediction of the synthesized compounds

**Figure 23:**
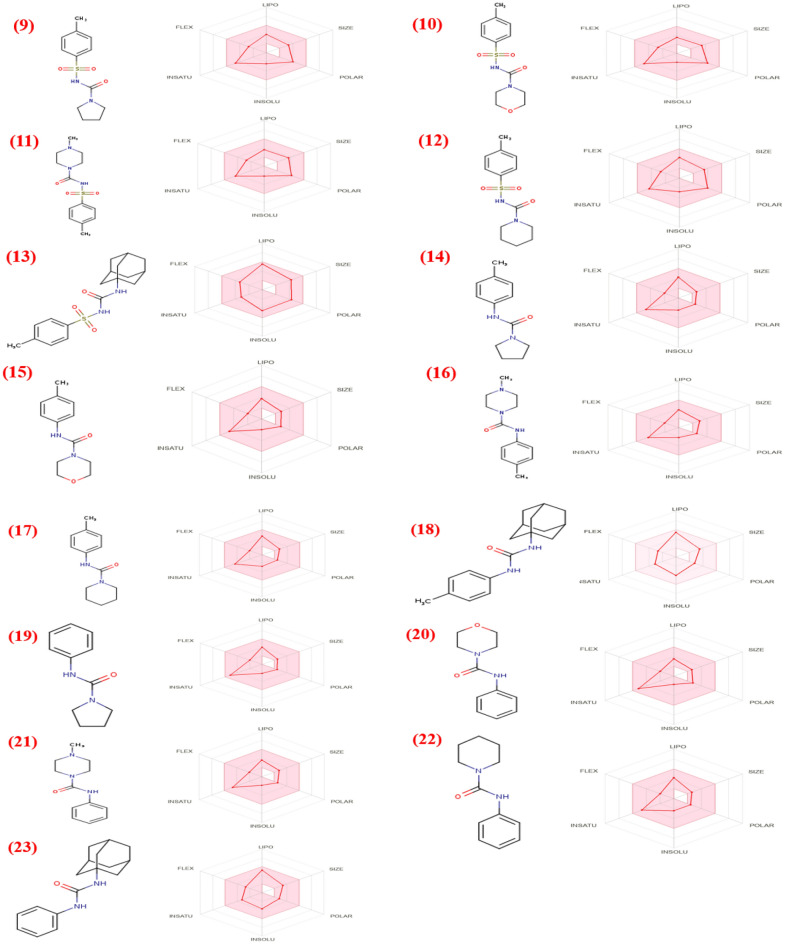
Oral bio-availability graph for compounds with the help of the Swiss ADME tool. Here, LIPO = lipophilicity as XLOGP3; SIZE = molecular weight; POLAR = polarity as topological polar surface area; INSOLU = insolubility in water by log S scale; INSATU = instauration as per fraction of carbons in the sp^3^ hybridization and FLEX = flexibility as per rotatable bonds.

**Figure 24:**
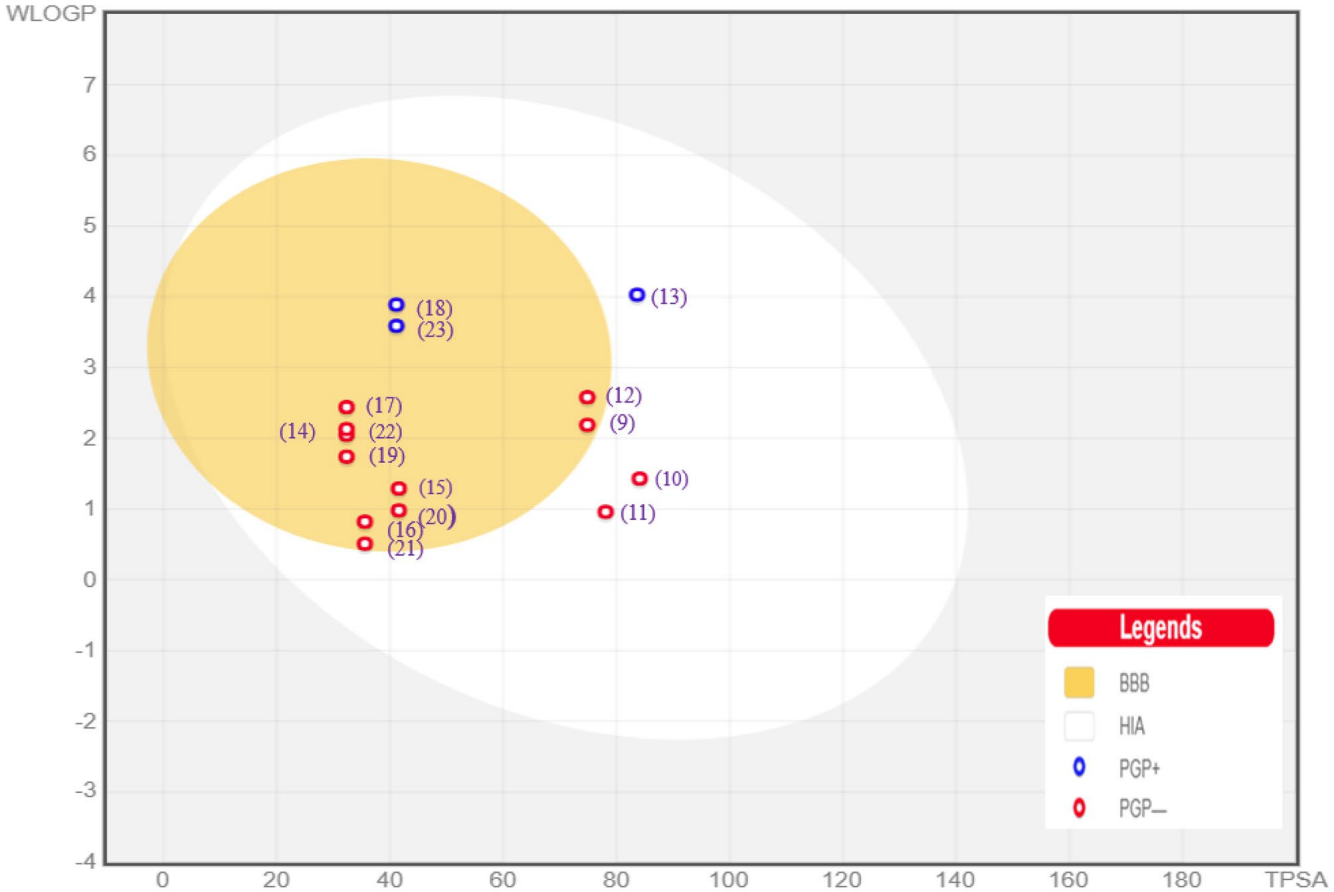
The boiled egg model for selected compounds. In terms of the medications being designed, white color stands for gastrointestinal absorption and yellow color for blood–brain barrier (BBB). The proposed drugs are depicted in red circles.

Based on the molecular docking results of all the synthesized compounds, we have selected the most promising ones with the highest affinity for protein receptors relevant to ADME and toxicity risks. Firstly, the physiochemical properties and ADME prediction of synthesized compounds are shown in Fig. 23 (Table S8, see supplementary information) the selected compounds underwent Lipinski rule testing. All the compounds were found to be Lipinski-compatible with MW less than 500, indicating their small size, easy transferability, and efficient absorption. Moreover, all the compounds possessed a sufficient number of rotatable bonds (RBs 1–6), which is crucial for high structural flexibility. This is important because compounds with less than ten RBs are more likely to be bioavailable. The hydrogen bond acceptors (HBA) and donors (HBD) were also calculated for all synthesized compounds, and it was found that all compounds had less than 10 HBA and less than 5 HBD, indicating a favorable balance of HBA and HBD and a higher likelihood of oral bioavailability. Secondly, we have assessed the lipophilicity and water solubility of all the compounds we selected. Our findings indicate that all active compounds are highly soluble in water, except **12**, **15**, **17** and **19** which have moderate solubility. The Log S values of these compounds range from − 3.17 to − 4.61, indicating high water solubility. Additionally, the lipophilicity parameter XLOGP3 of all compounds appeared to fall within the allowed range of XLOGP3 between (− 0.7 and + 5.0). Thirdly, we have conducted tests on the pharmacokinetics of the compounds. Our results suggest that all synthesized compounds can cross the blood–brain barrier except compounds **16**, **17** and **19**. As well as all synthesized compounds exhibit high intestinal absorption. Additionally, most of the synthesized compounds have the potential to interact with other drugs as they can suppress the CYP1A2, CYP2D6, and CYP3A4 enzymes (Fig. 24). Fourthly, the study appears to have evaluated the drug-likeness and lead-likeness of certain compounds using various methods, including the Lipinski, Ghose, Veber, Muegge, and Egan rules. It is encouraging that all compounds met the requirements of all the drug-likeness methods that were applied and that most of them also met the requirements of lead-likeness, indicating that they have desirable physicochemical properties for drug development. The calculated bioavailability of all molecules being 0.55 is also a positive sign, suggesting that these compounds have the potential to be absorbed into the bloodstream and reach their intended target. Overall, these findings suggest that the compounds meeting all the drug-likeness criteria may be good starting points for drug discovery and further development as potential drugs. Finally, it appears that all the synthesized compounds were found to be non-tumorigenic, non-mutagenic, and non-irritant, and did not exhibit any reproductive toxicity. These are principal factors to consider when developing new drugs or therapeutic agents. Furthermore, Topological Polar Surface Area (TPSA) values of the compounds were found to be relatively low, with most falling below the optimal range of 90–140 for good absorption in the gut and oral bioavailability as depicted in Table 6.

**Table 6:** Prediction of toxicity risks and physicochemical properties of compounds.

Ligand	Toxicity risks				Physicochemical properties					
	Mutagenic	Tumorigenic	Irritant	Reproductive	cLogP	Solubility	Molecular Weight	TPSA	Drug likeness	Drug score
**9**	(−)	(−)	(−)	(−)	1.97	− 2.30	268.33	74.86	0.94	0.47
**10**	(−)	(−)	(−)	(−)	1.15	− 1.68	284.33	84.09	0.38	0.45
**11**	(−)	(−)	(−)	(−)	1.26	− 1.18	297.37	78.1	0.45	0.56
**12**	(−)	(−)	(−)	(−)	2.31	− 2.57	282.36	74.86	− 0.60	0.36
**13**	(−)	(−)	(−)	(−)	2.99	− 4.31	348.46	83.65	0.90	0.37
**14**	(−)	(−)	(−)	(−)	2.49	− 2.81	204.27	32.34	2.02	0.85
**15**	(−)	(−)	(−)	(−)	1.67	− 2.19	220.27	41.57	1.53	0.86
**16**	(−)	(−)	(−)	(−)	1.78	− 1.69	233.31	35.58	6.82	0.94
**17**	(−)	(−)	(−)	(−)	2.84	− 3.08	218.29	32.34	0.54	0.71
**18**	(−)	(−)	(−)	(−)	3.51	− 4.82	284.4	41.13	0.53	0.55
**19**	(−)	(−)	(−)	(−)	2.15	− 2.46	190.24	32.34	1.78	0.86
**20**	(−)	(−)	(−)	(−)	1.33	− 1.84	206.24	41.57	1.27	0.85
**21**	(−)	(−)	(−)	(−)	1.44	− 1.34	219.28	35.58	6.69	0.96
**22**	(−)	(−)	(−)	(−)	2.49	− 2.73	204.27	32.34	0.32	0.71
**23**	(−)	(−)	(−)	(−)	3.17	− 4.47	270.37	41.13	0.17	0.57

*(+) The molecule is known to be mutagenic, tumorigenic, irritant, and reproductive effects.

*(−**)** No indication for Mutagenicity, Tumorgenicity, irritation, and reproductive effect.

*Drug-likeness score: Predicts an overall drug-likeness score using chemical fingerprints.

*TPSA is defined as the sum of the topological surface area of oxygens, nitrogen, and attached hydrogens.

*logP value is the logarithm of its partition coefficient between n-octanol and water.

## Conclusion

Herein, we report facile access to some new series of unsymmetrical carbamide derivatives bearing different aliphatic amine moieties. This convenient method does not need any catalyst by using AC or DCM as solvent at room temperature. All the synthesized unsymmetrical carbamide derivatives were tested as antimicrobial agents against some clinically bacterial pathogens such as *Salmonella typhimurium*, *Bacillus subtilis, Pseudomonas aeruginosa*, *Staphylococcus aureus* and *Candida albicans*. A significant inhibitory effect was obtained by compounds **15** and **22**, which provide a potent lipid peroxidation of the bacterial cell wall. Moreover, all the synthesized compounds were investigated as anti-proliferative agents against selected human cancerous cell lines of breast (MCF-7), colon (HCT-116), and lung (A549) relative to healthy noncancerous control skin fibroblast cells (BJ-1). Cell cycle arrest, and necrosis cell apoptosis were discussed for the most active compounds. Furthermore, computational studies such as molecular docking, physicochemical and in silico pharmacokinetics were reported for discussing the binding affinity of these compounds regarding the targeted proteins.

### Supplementary Information

Supplementary Information.

## Data Availability

The data that support the findings of this study are available from the corresponding author upon reasonable request.
