# Supplementary material for: Green, facile synthesis and evaluation of unsymmetrical carbamide derivatives as antimicrobial and anticancer agents with mechanistic insights
Source: Sci Rep. 2024 Jul 4;14:15441. doi: 10.1038/s41598-024-65308-6 (PMC11224357; doi:10.1038/s41598-024-65308-6)
Supplement: Supplementary file 1 — Supplementary Information. [file 41598_2024_65308_MOESM1_ESM.docx]

***Supporting information of***

**Green, Facile Synthesis and Evaluation of Unsymmetrical Carbamide Derivatives as Antimicrobial and Anticancer agents with Mechanistic Insights**

Farid M. Sroor^1,^ *, Ahmed A. F. Soliman^2^, Elham Mohamed Youssef^3^, Mohamed Abdelraof^4^, Ahmed F. El-Sayed^5,6^

1. Organometallic and Organometalloid Chemistry Department, National Research Centre, 12622 Cairo, Egypt
2. Pharmacognosy Department, National Research Centre, 12622-Dokki, Egypt
3. Biochemistry Department, National Research Centre, Cairo, Egypt
4. Microbial Chemistry Department, Biotechnology Research Institute, National Research Centre, Giza, Egypt
5. Microbial Genetics Department, Biotechnology Research Institute, National Research Centre, Giza, Egypt
6. Egypt Center for Research and Regenerative Medicine (ECRRM), Cairo, Egypt

Correspondence author

Farid M Sroor, Email: [faridsroor@gmx.de](mailto:faridsroor@gmx.de), [fm.sroor@nrc.sci.eg](mailto:fm.sroor@nrc.sci.eg)


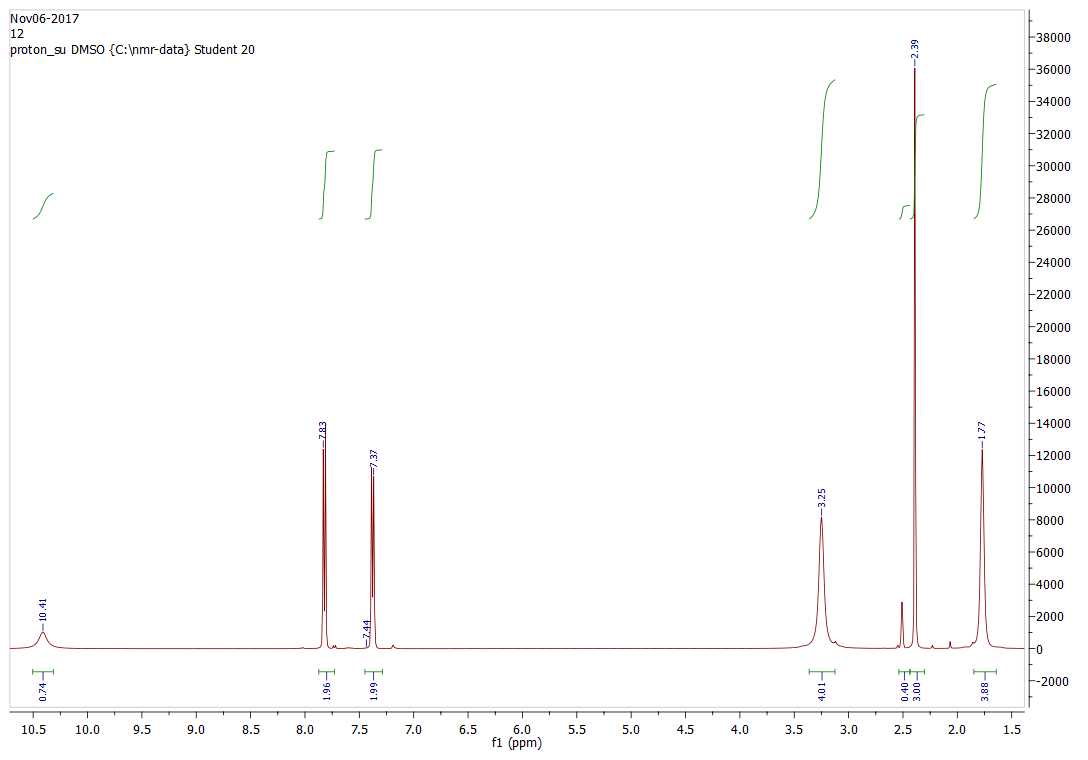


^1^H-NMR spectrum of **9**.


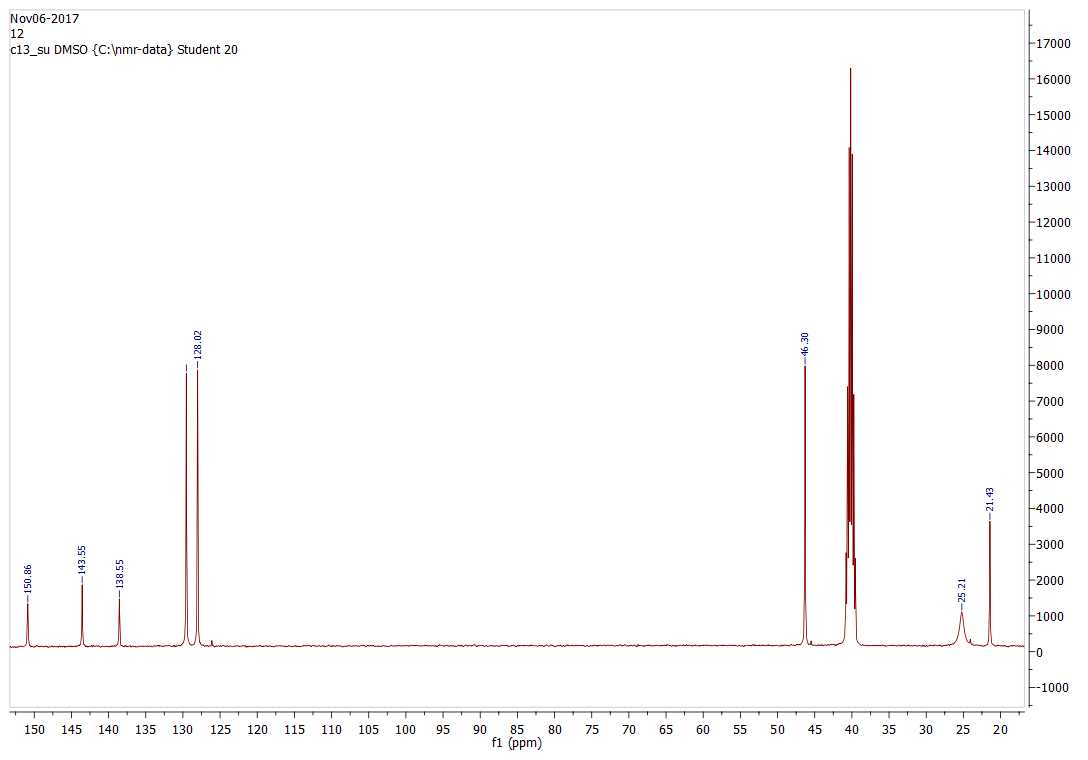


^13^C-NMR spectrum of **9**.


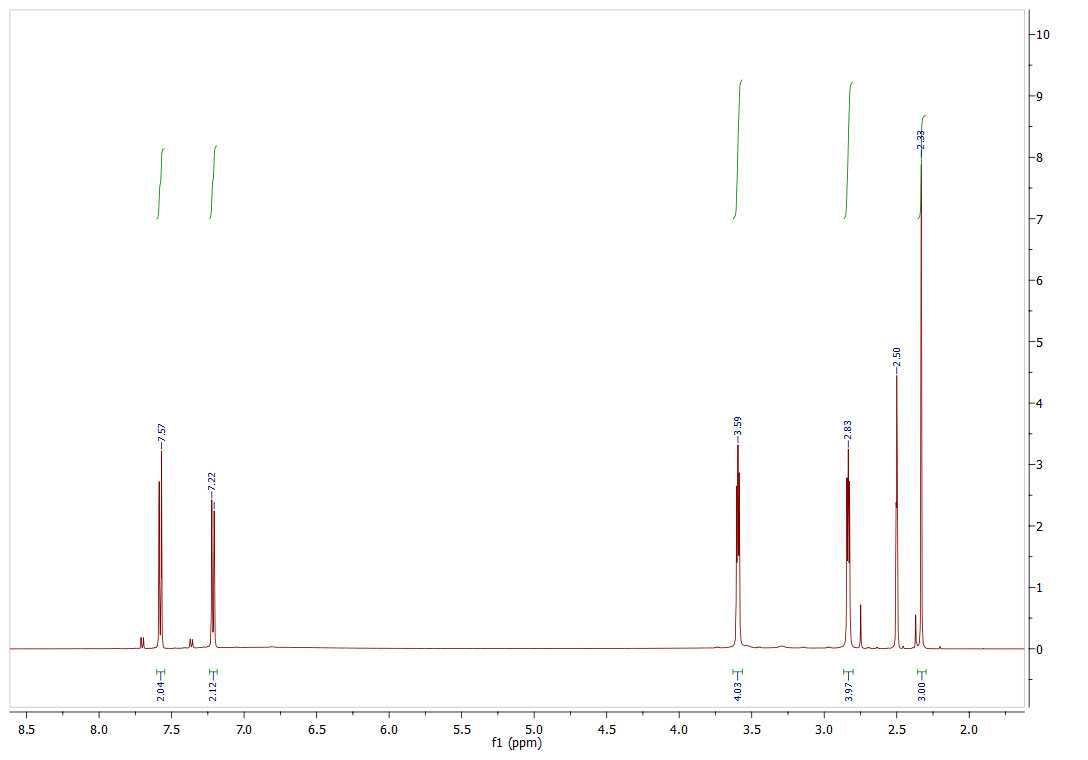


^1^H-NMR spectrum of **10**.


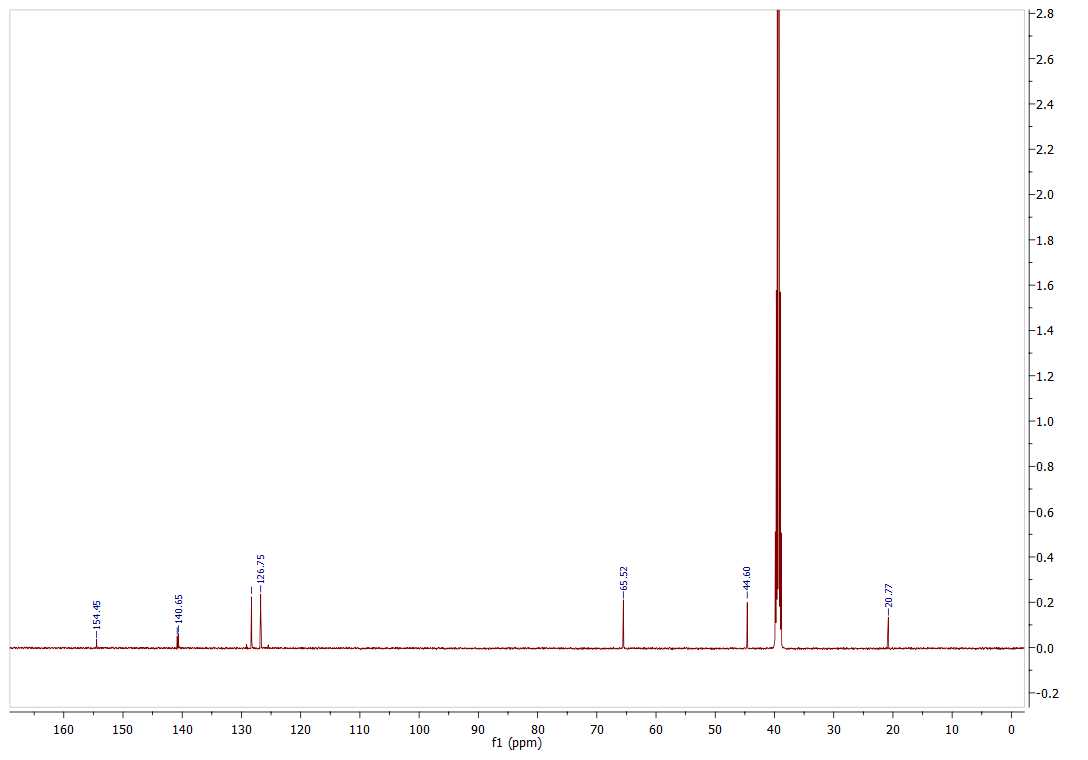


^13^C-NMR spectrum of **10**.


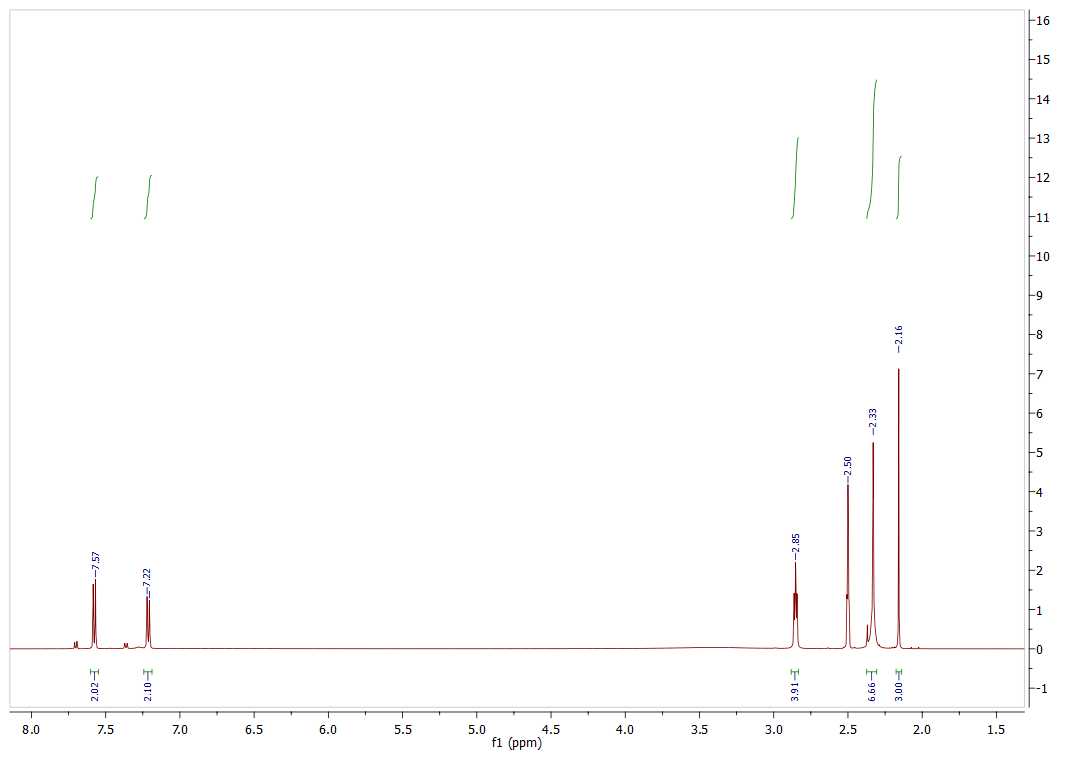


^1^H-NMR spectrum of **11**.


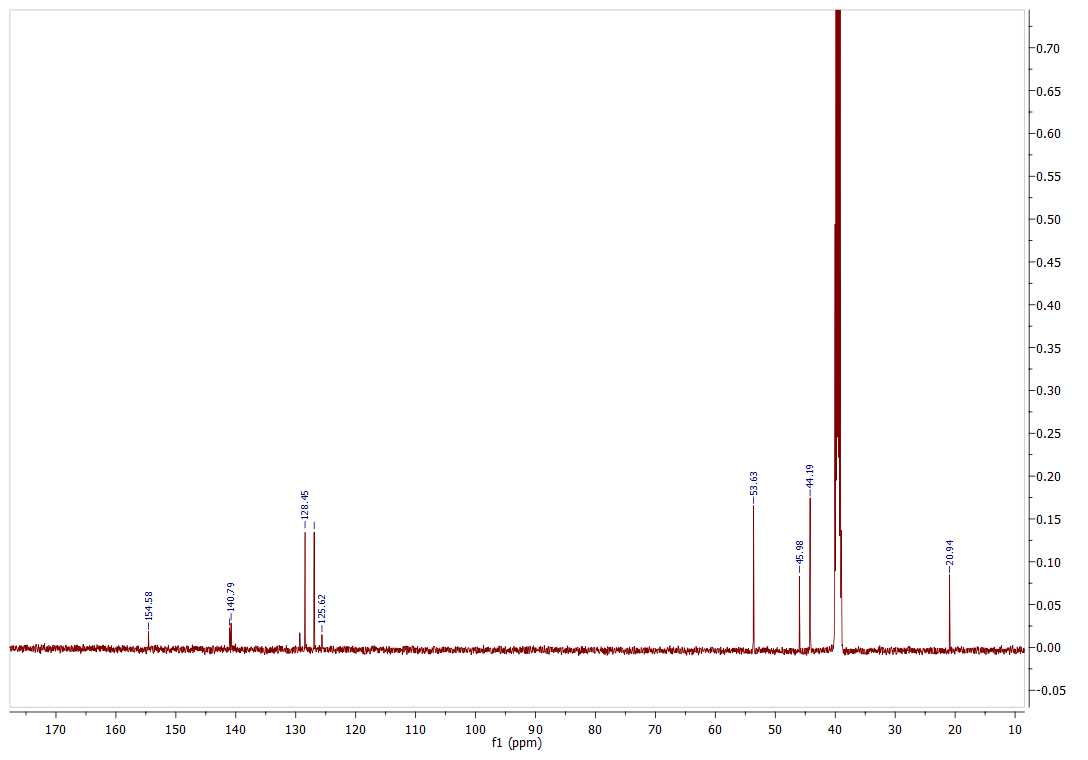


^13^C-NMR spectrum of **11**.


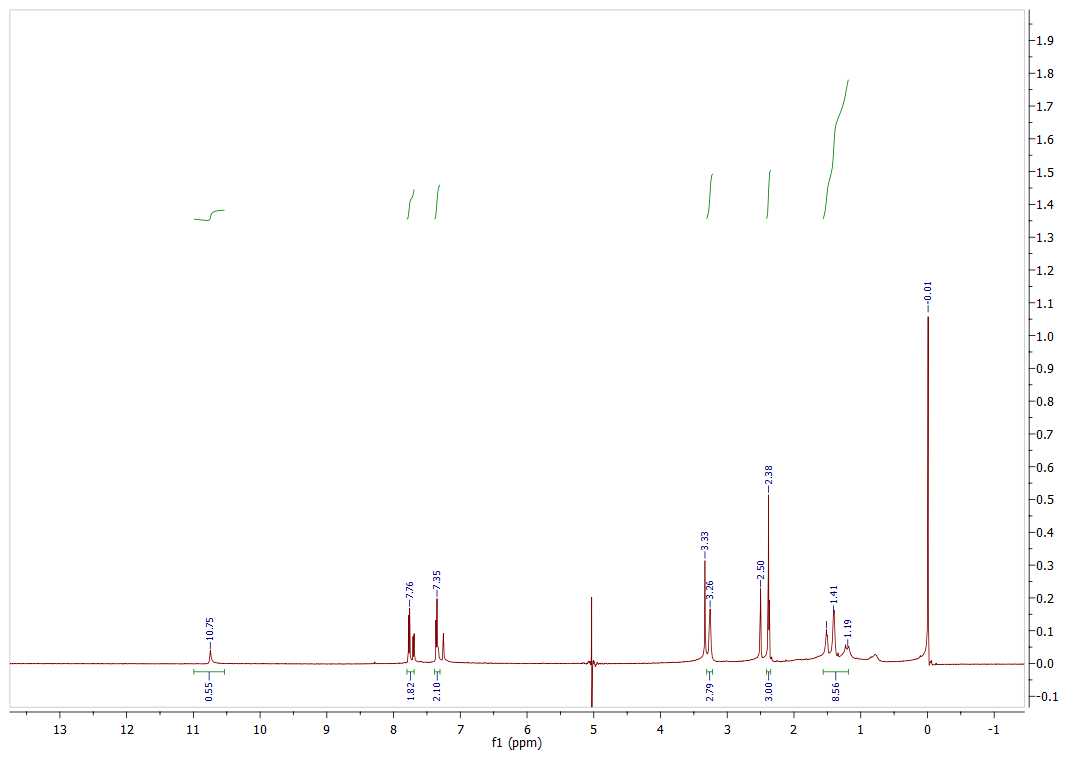


^1^H-NMR spectrum of **12**.


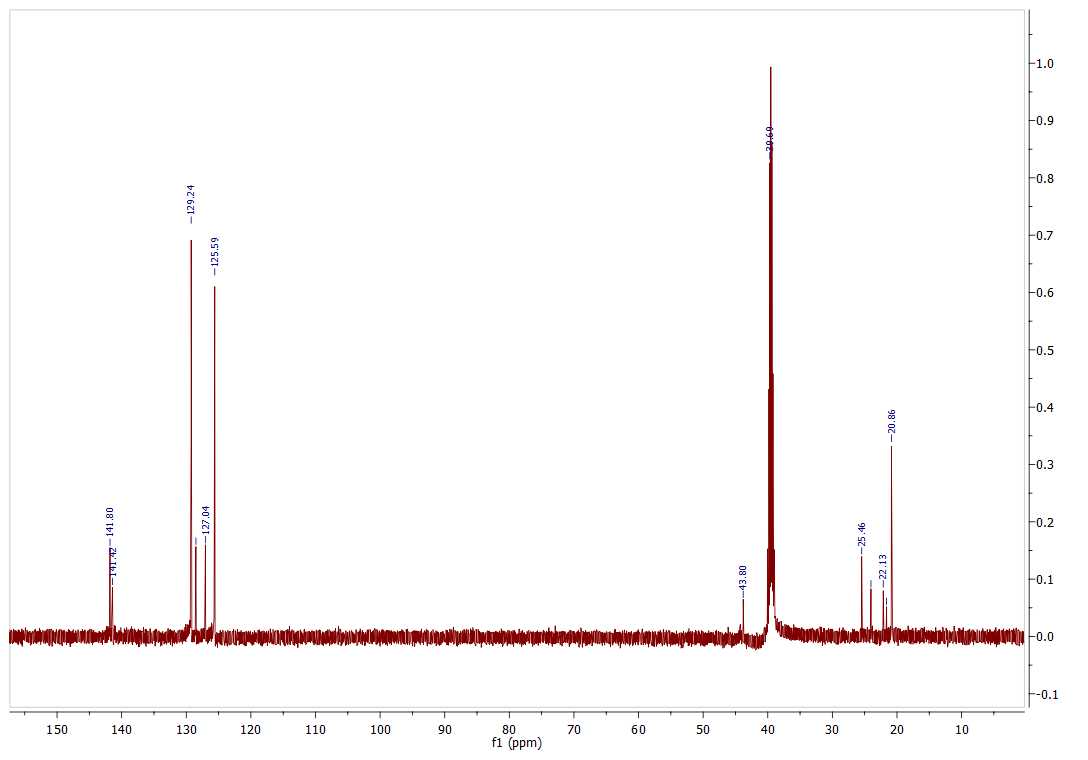


^13^C-NMR spectrum of **12**.


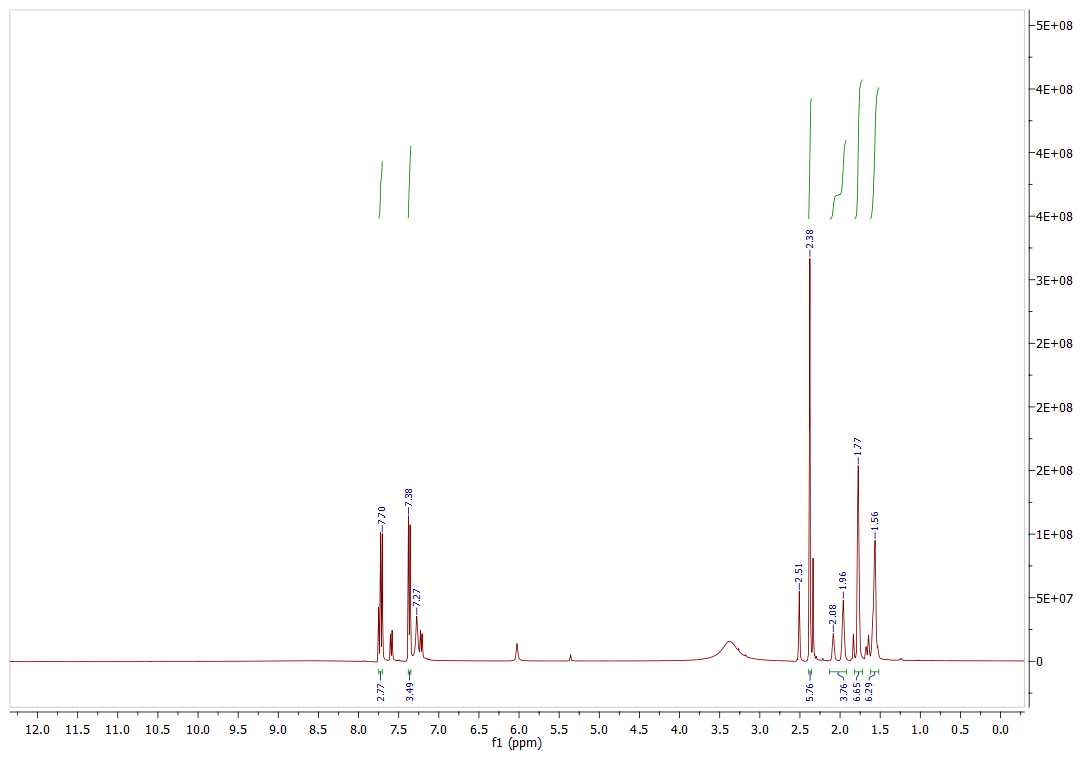


^1^H-NMR spectrum of **13**.


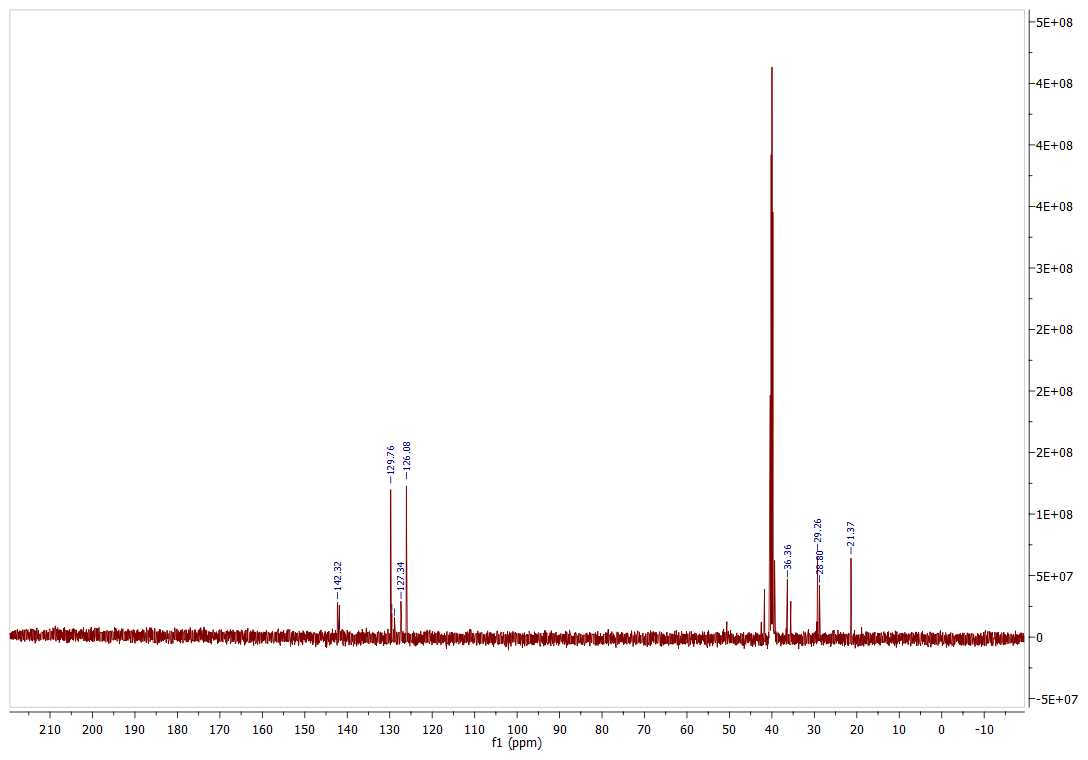


^13^C-NMR spectrum of **13**.


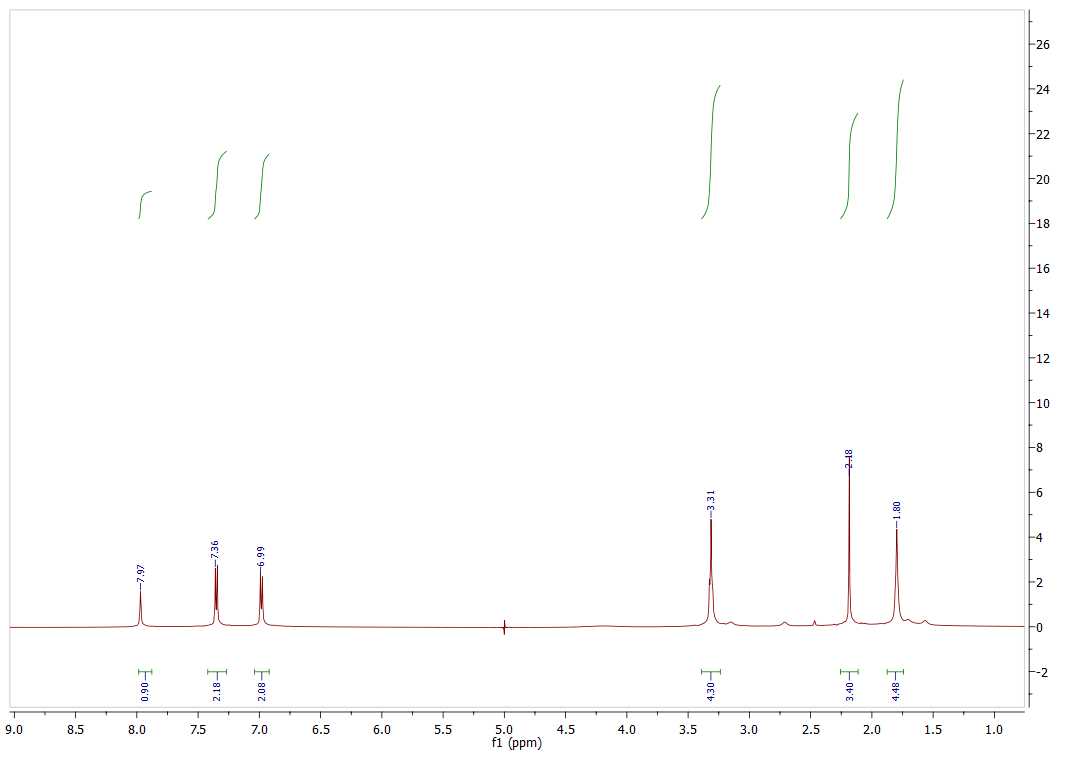


^1^H-NMR spectrum of **14**.


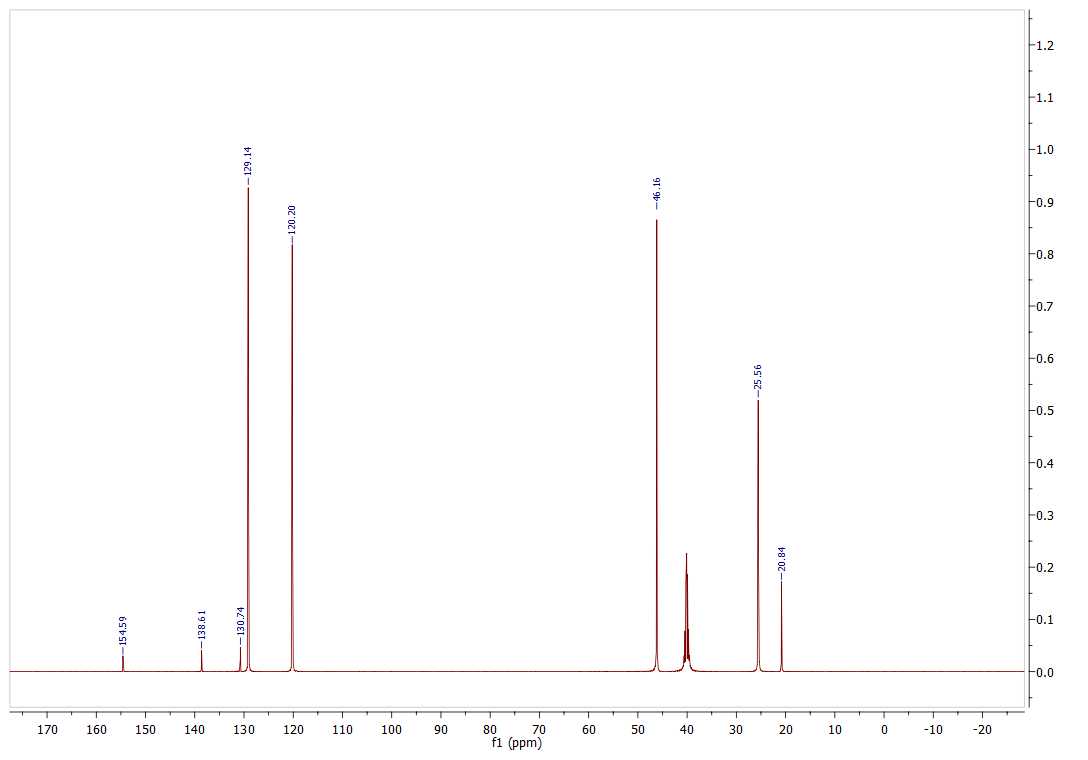


^13^C-NMR spectrum of **14**.


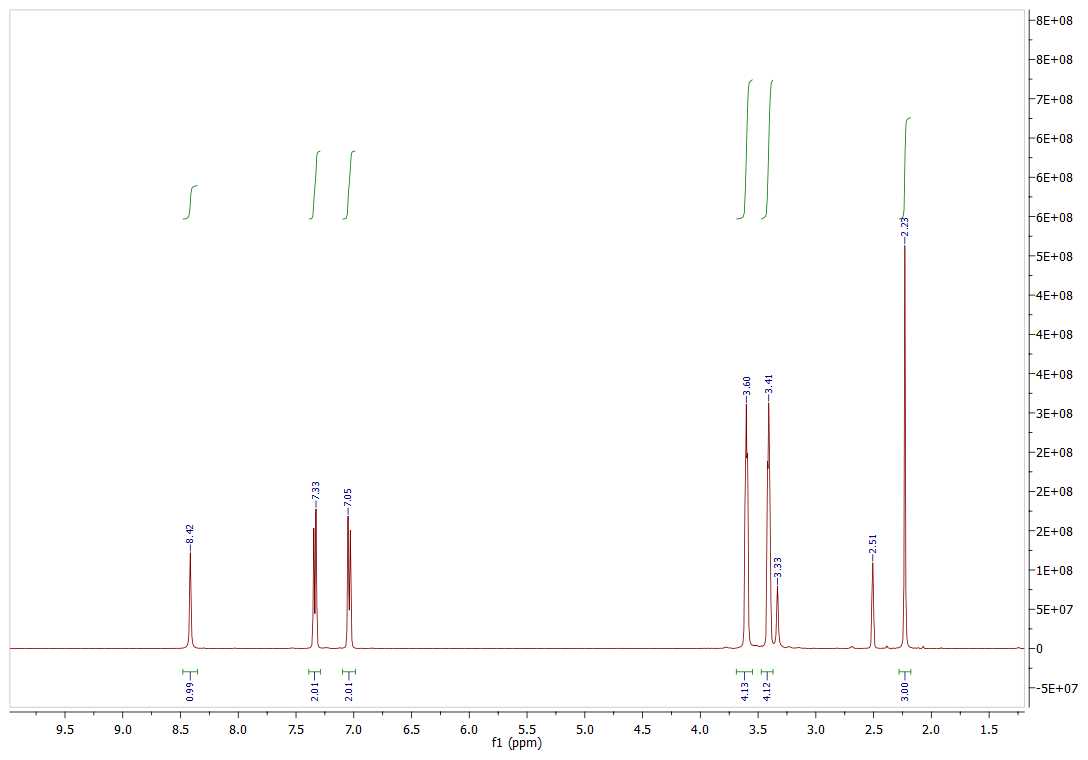


^1^H-NMR spectrum of **15**.


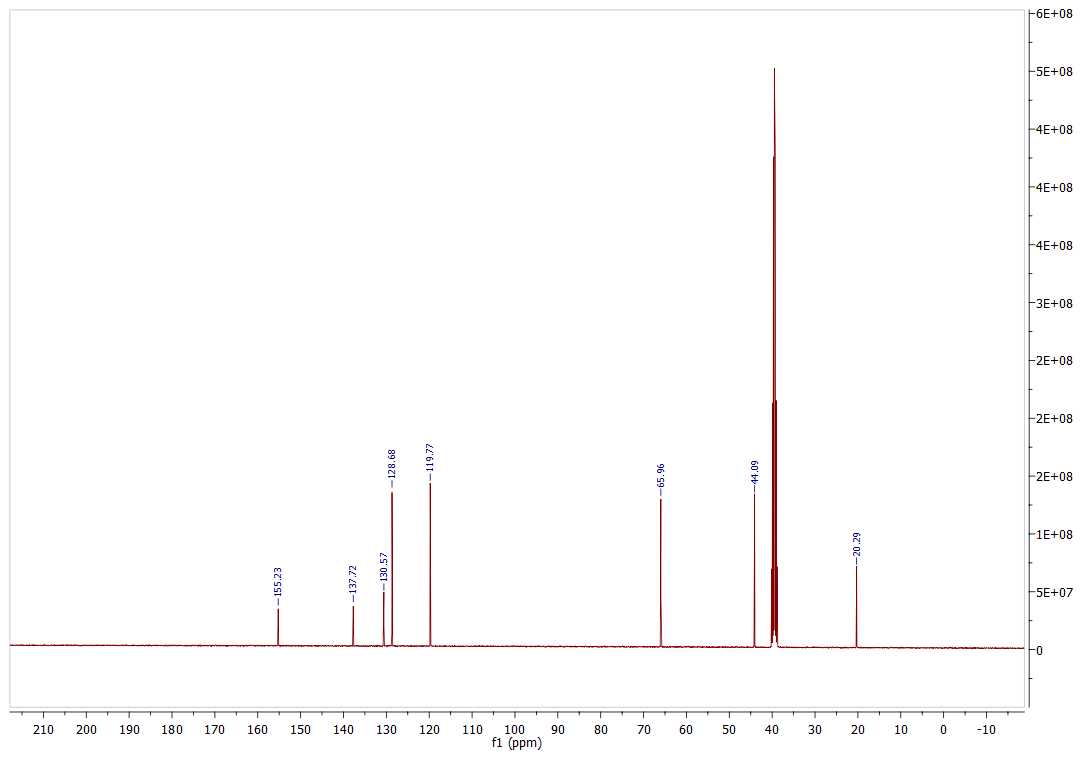


^13^C-NMR spectrum of **15**.


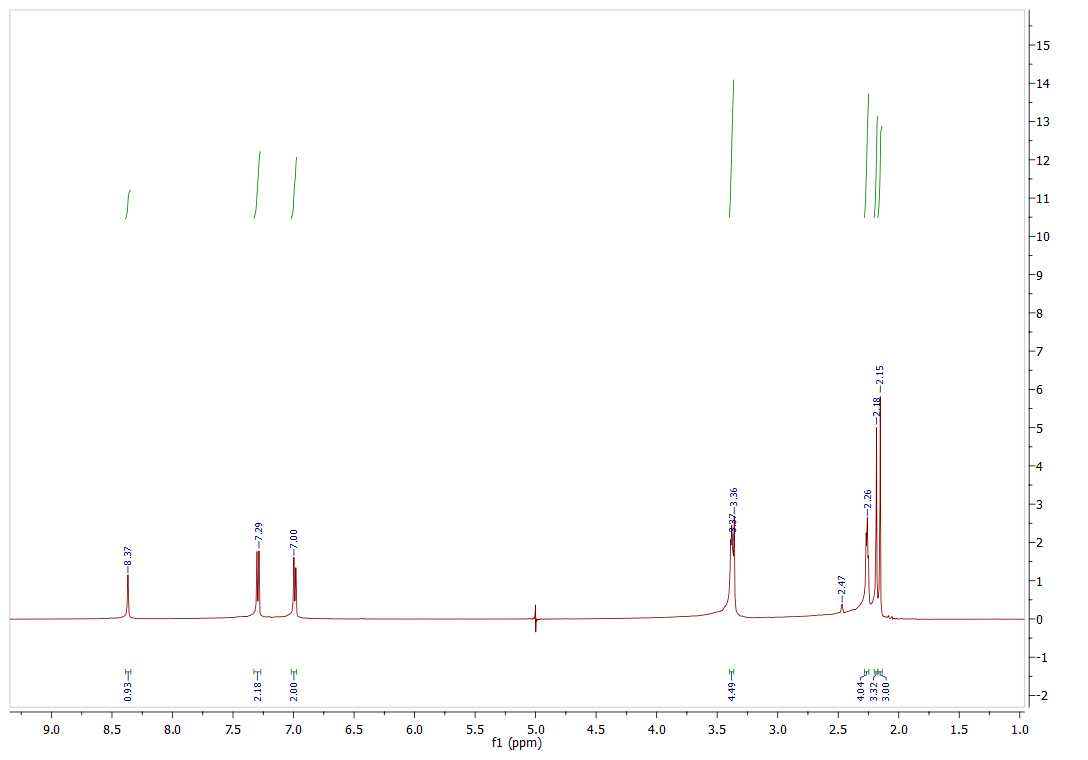


^1^H-NMR spectrum of **16**.


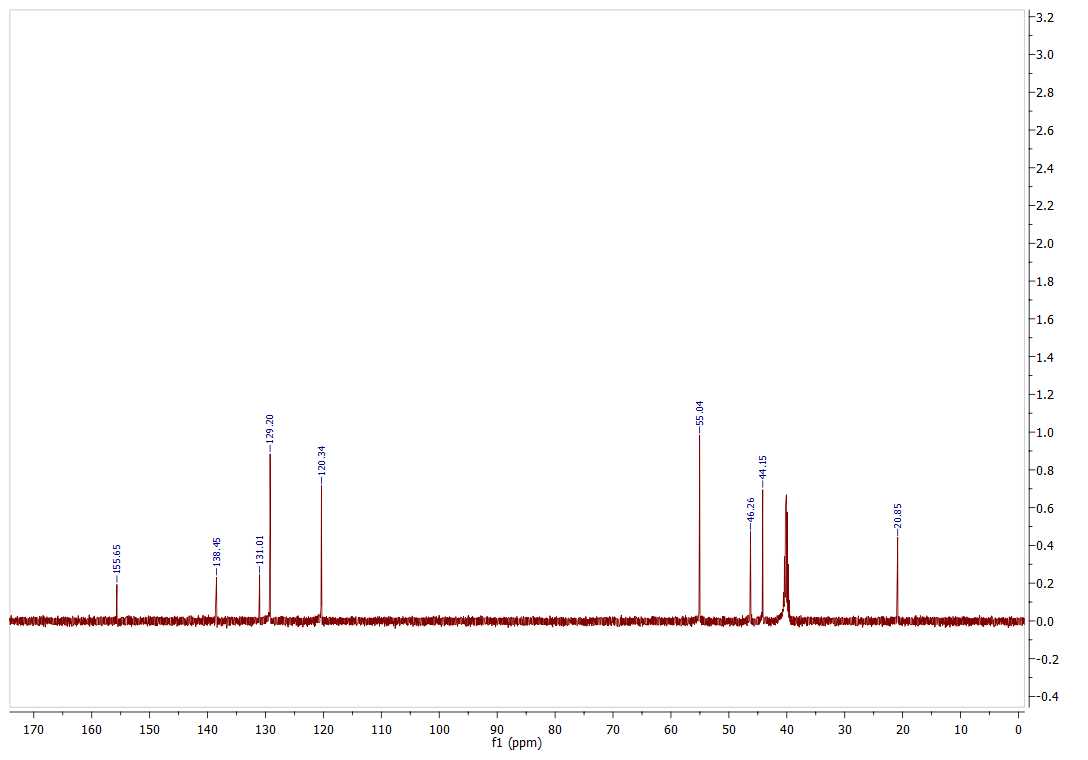


^13^C-NMR spectrum of **16**.


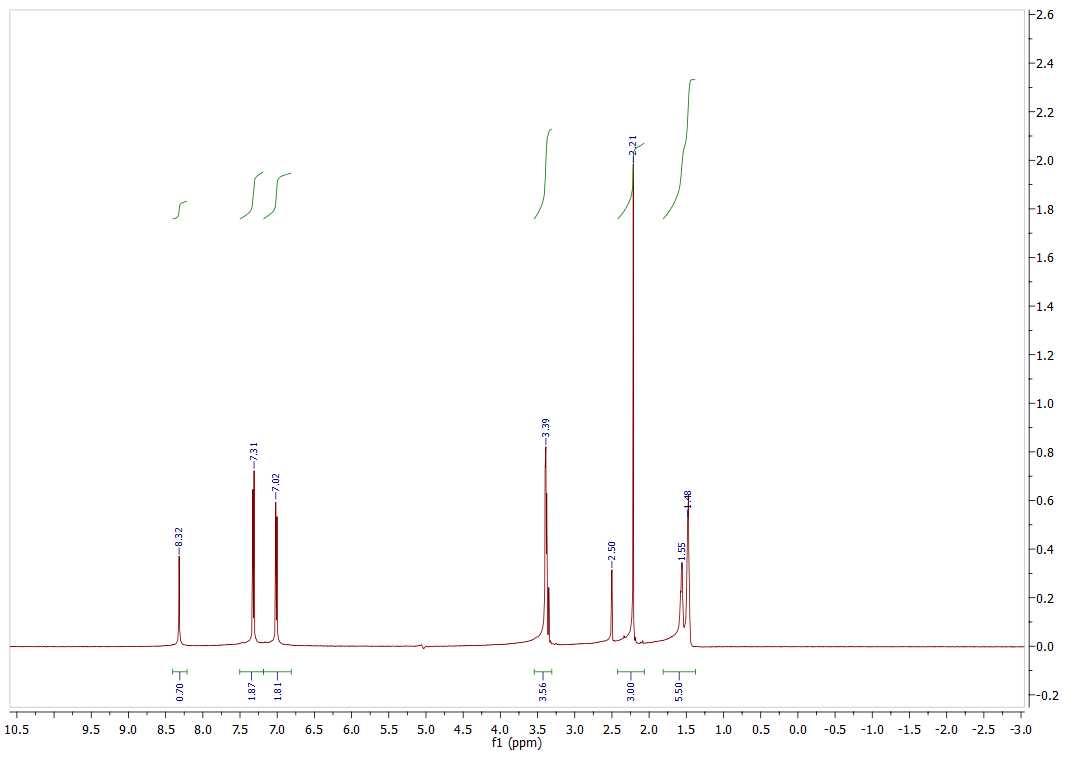


^1^H-NMR spectrum of **17**.


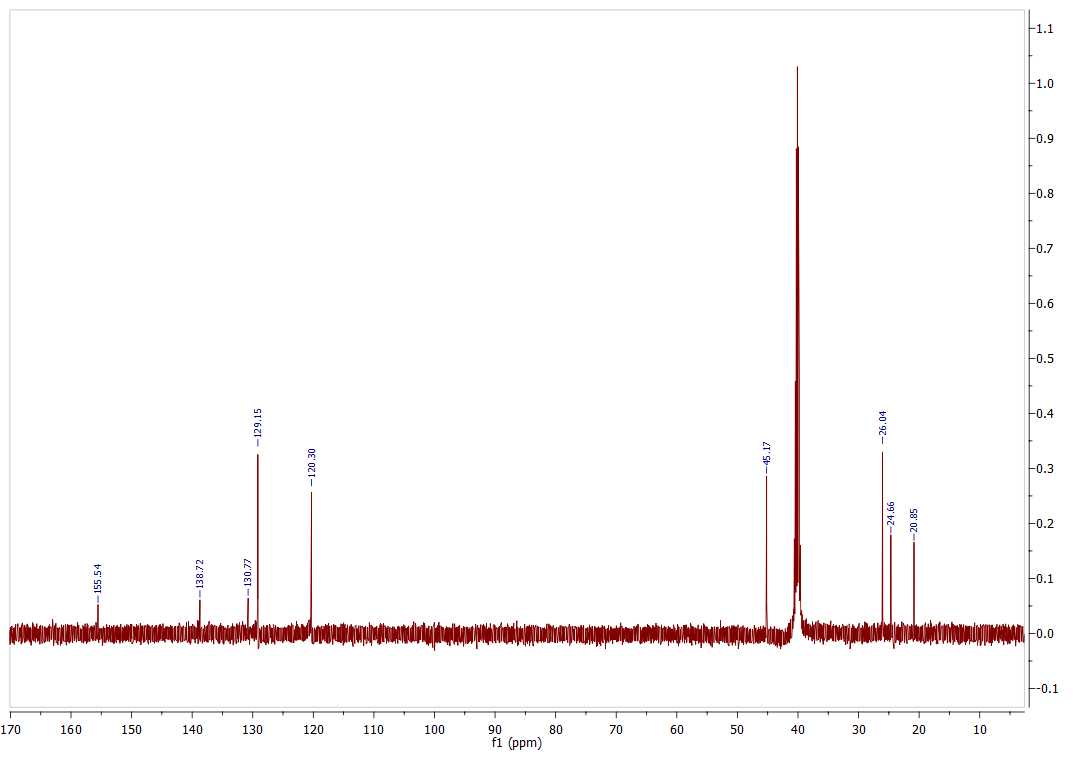


^13^C-NMR spectrum of **17**.


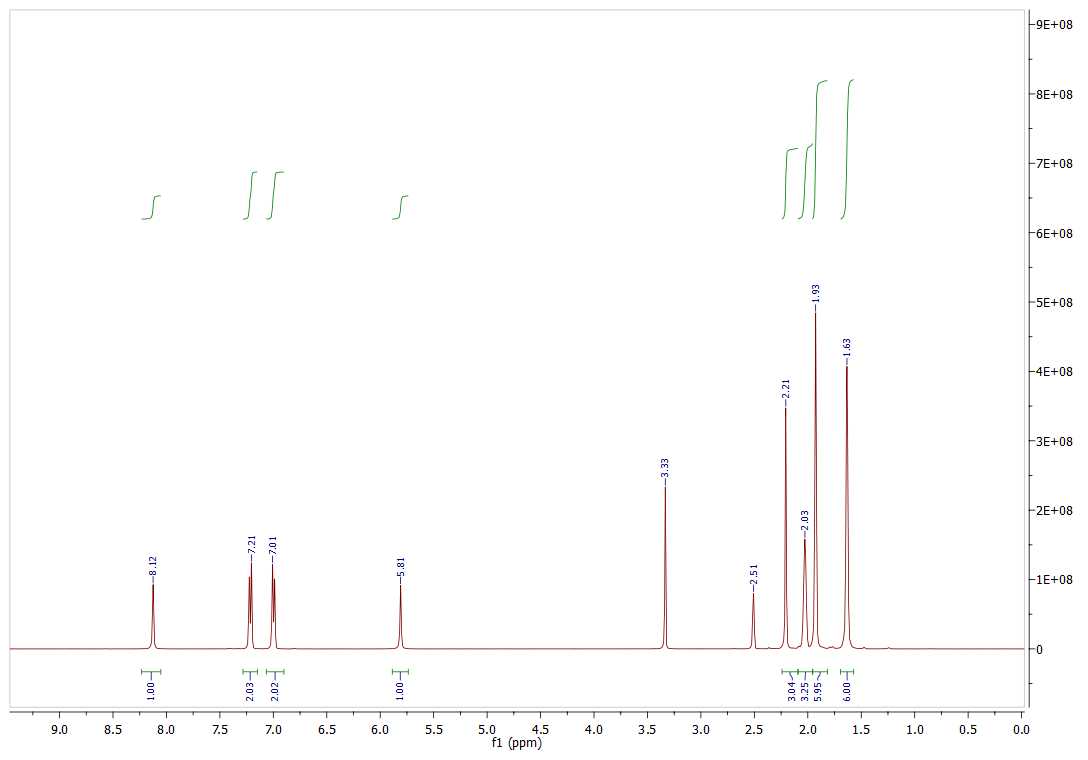


^1^H-NMR spectrum of **18**.


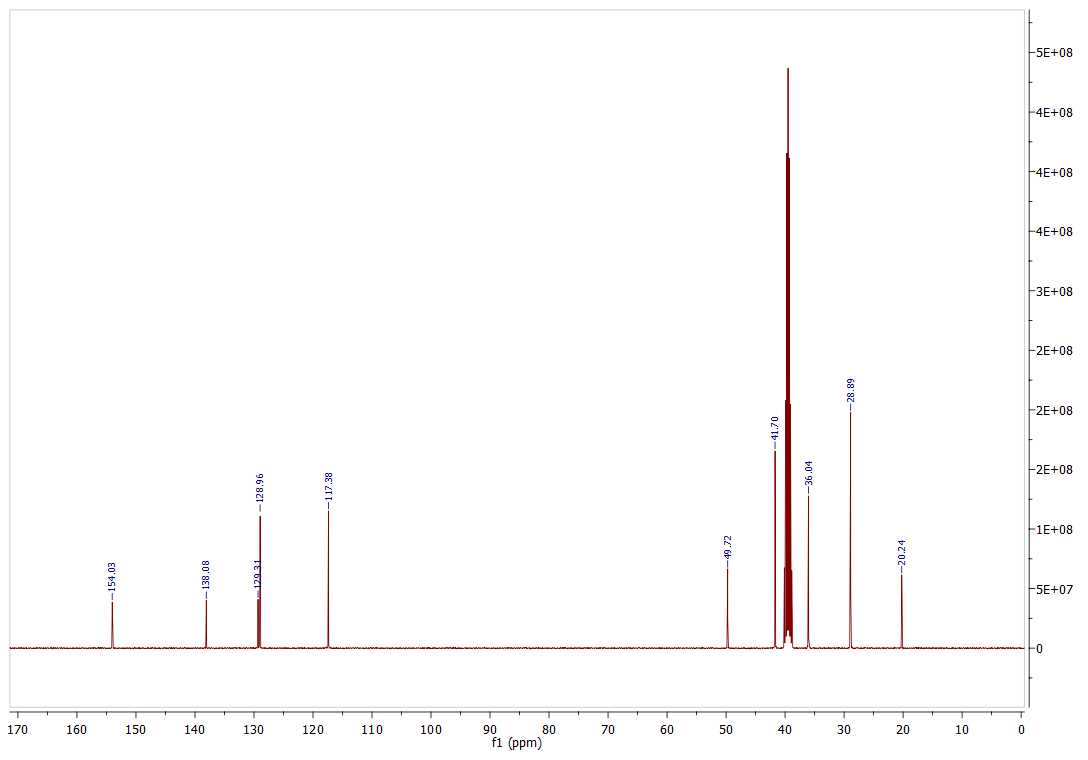


^13^C-NMR spectrum of **18**.


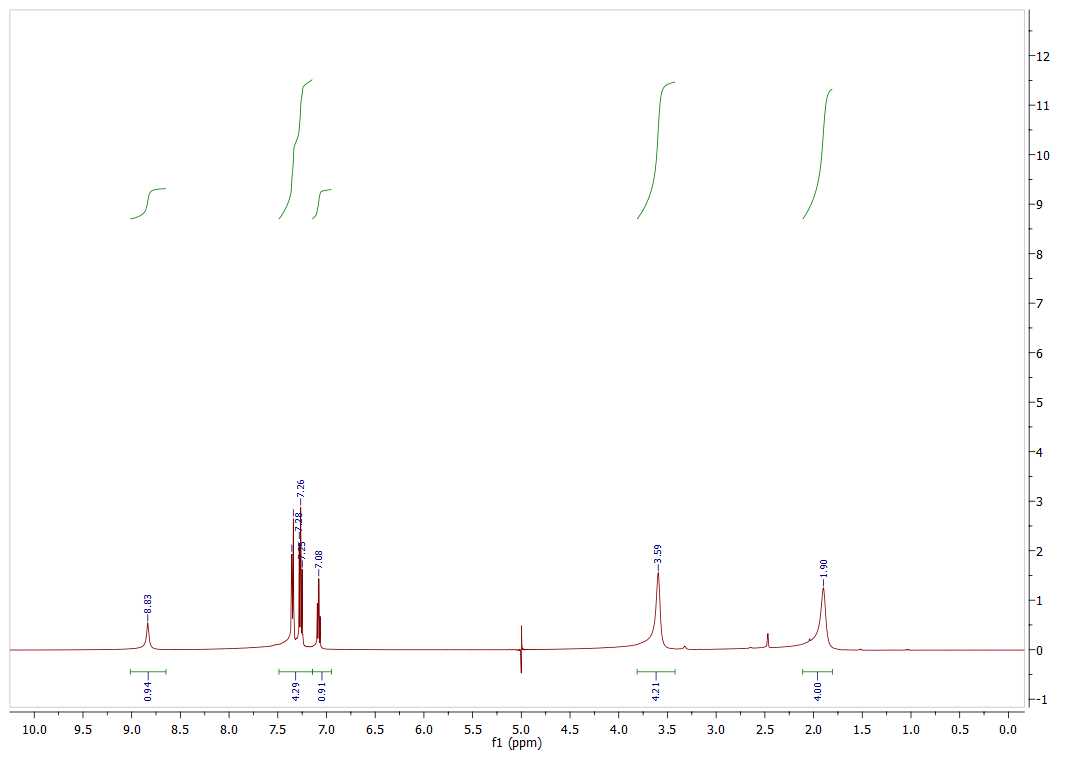


^1^H-NMR spectrum of **19**.


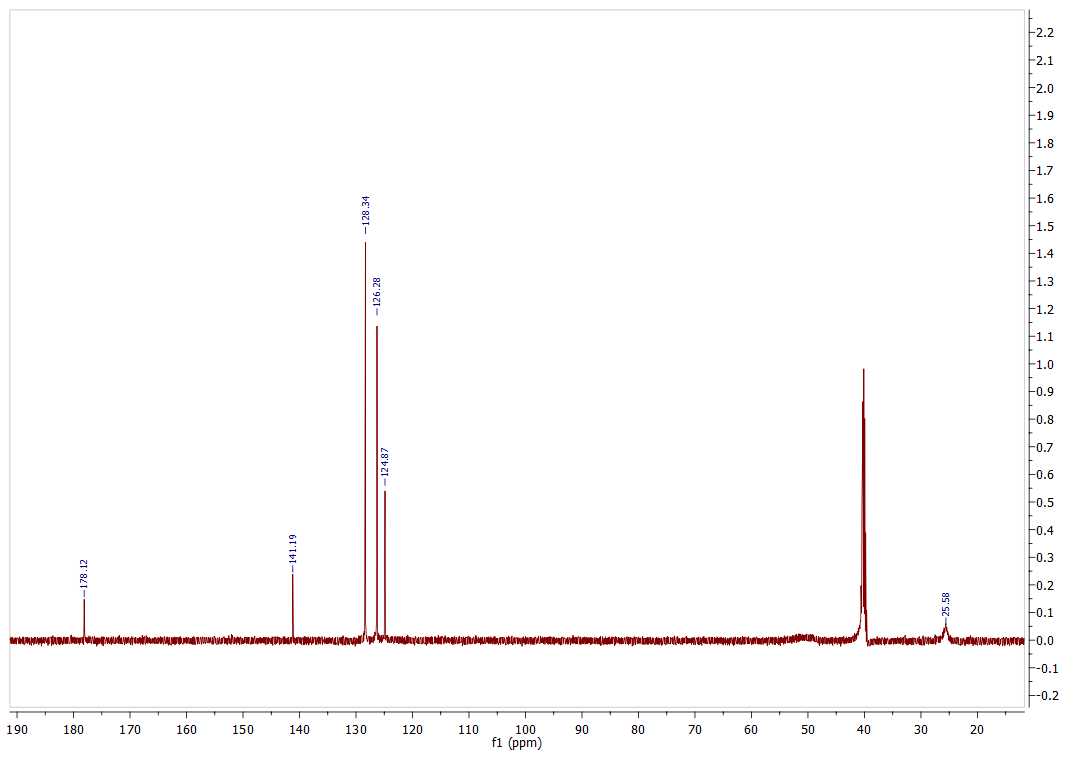


^13^C-NMR spectrum of **19**.


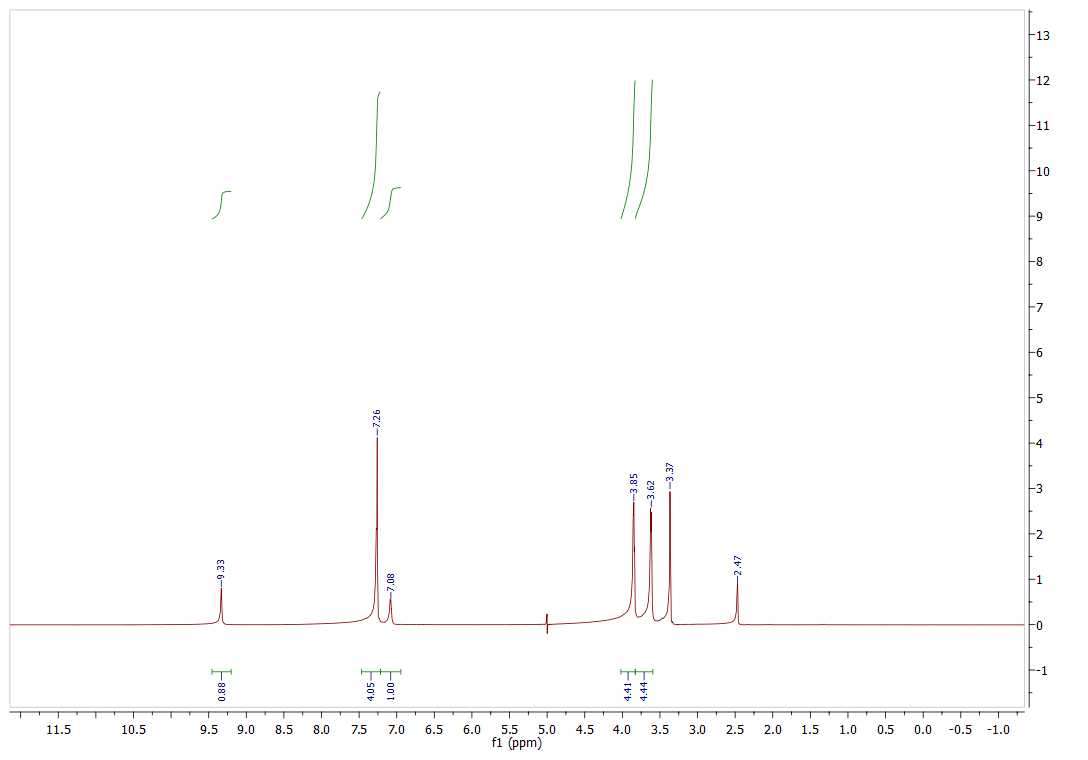


^1^H-NMR spectrum of **20**.


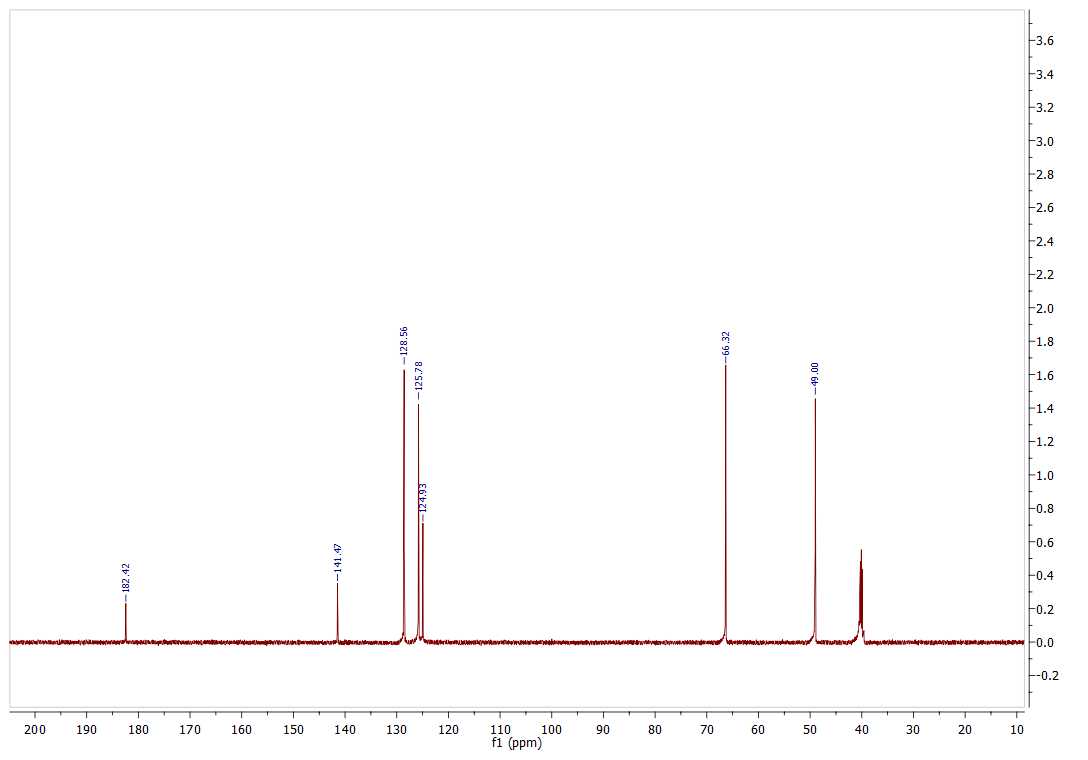


^13^C-NMR spectrum of **20**.


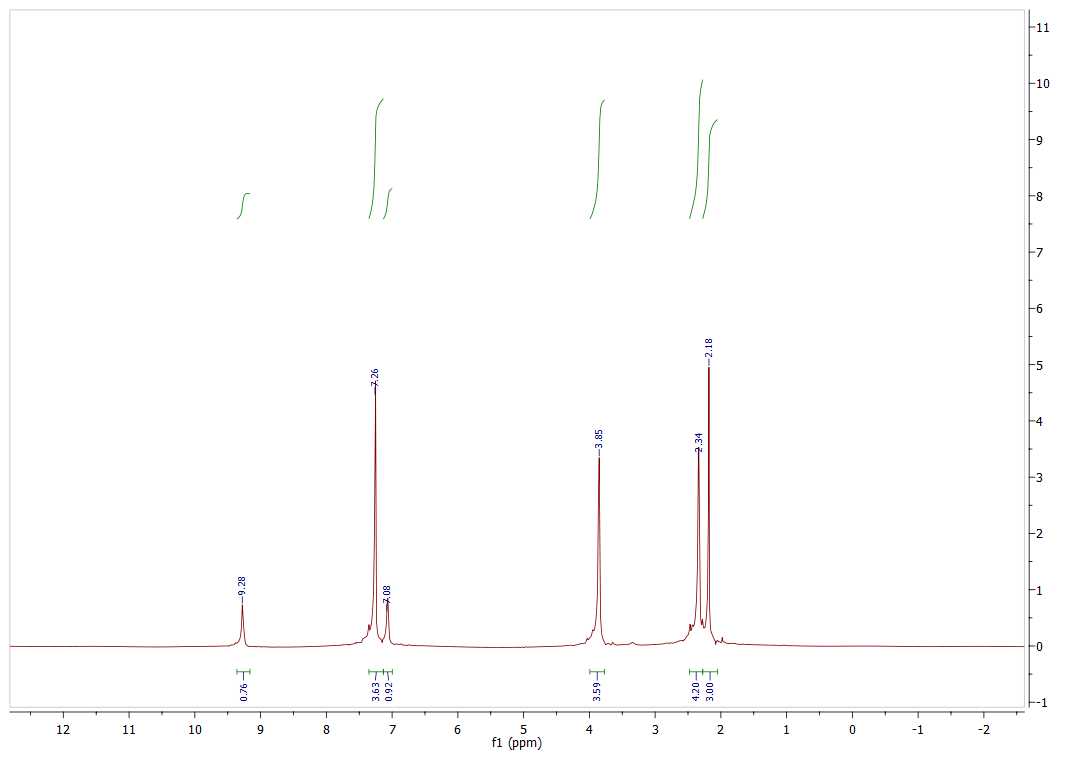


^1^H-NMR spectrum of **21**.


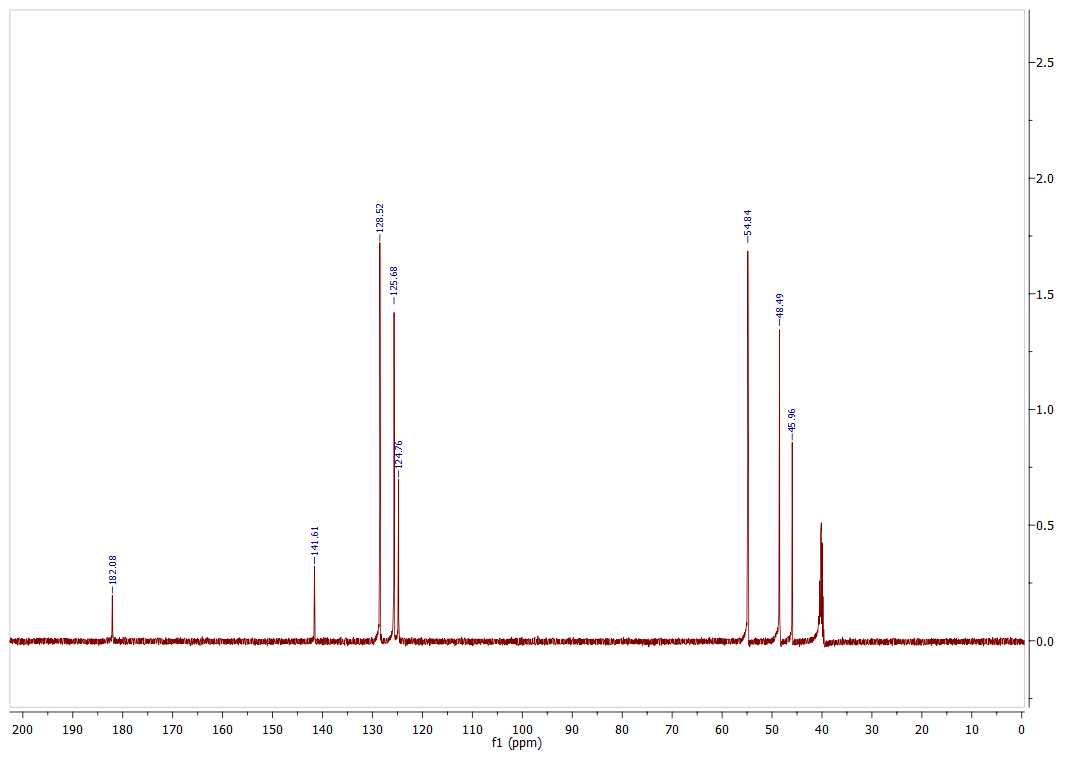


^13^C-NMR spectrum of **21**.


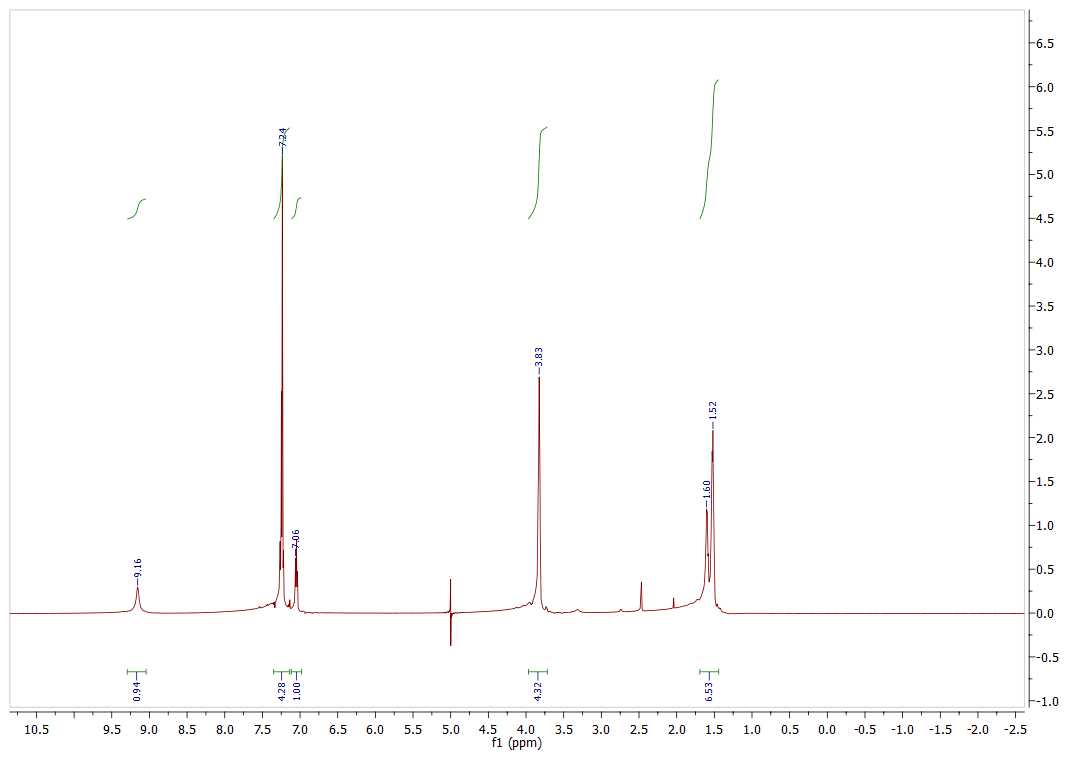


^1^H-NMR spectrum of **22**.


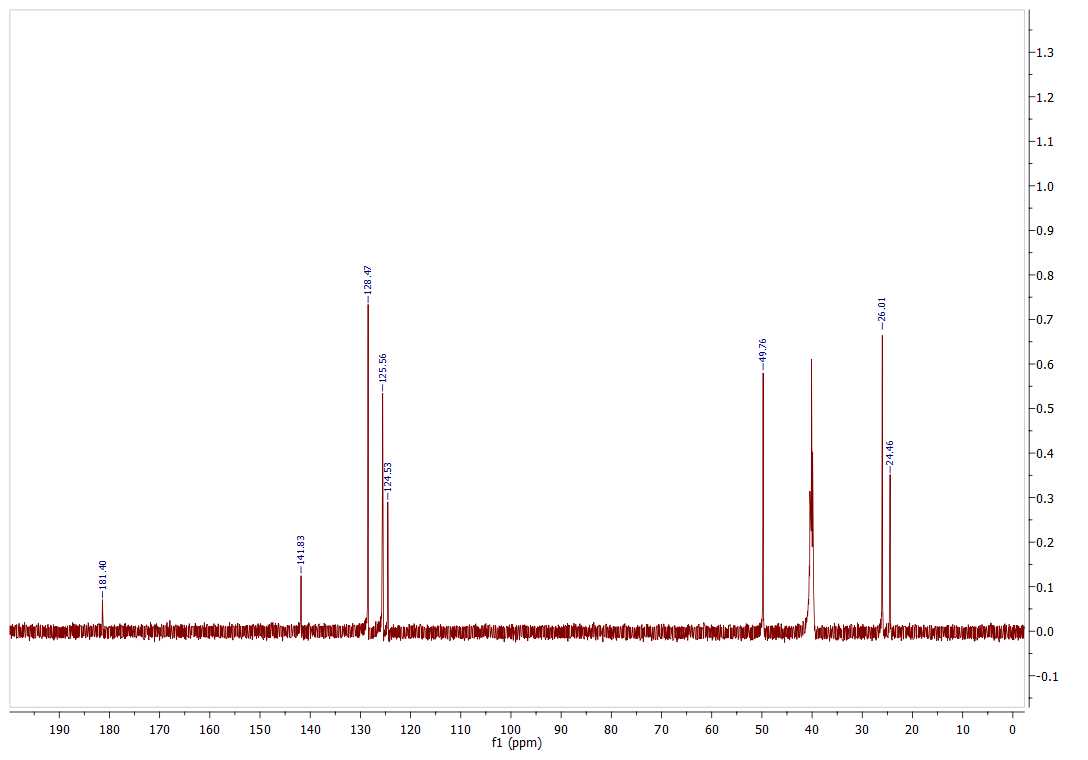


^13^C-NMR spectrum of **22**.


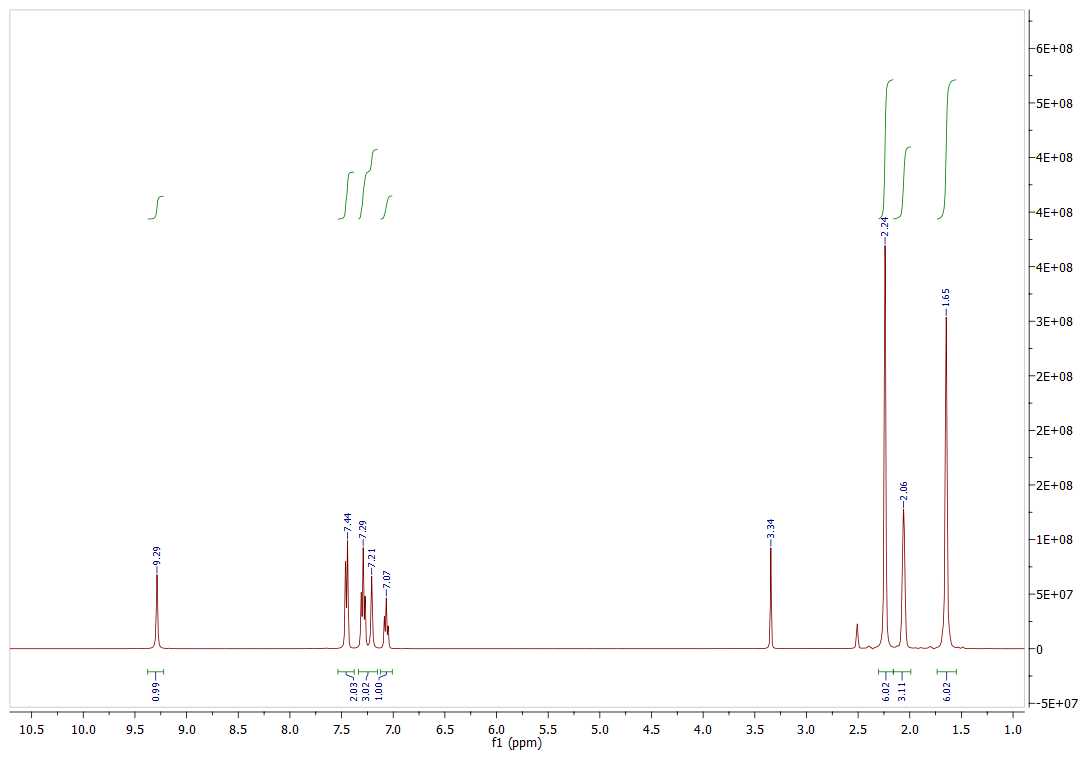


^1^H-NMR spectrum of **23**.


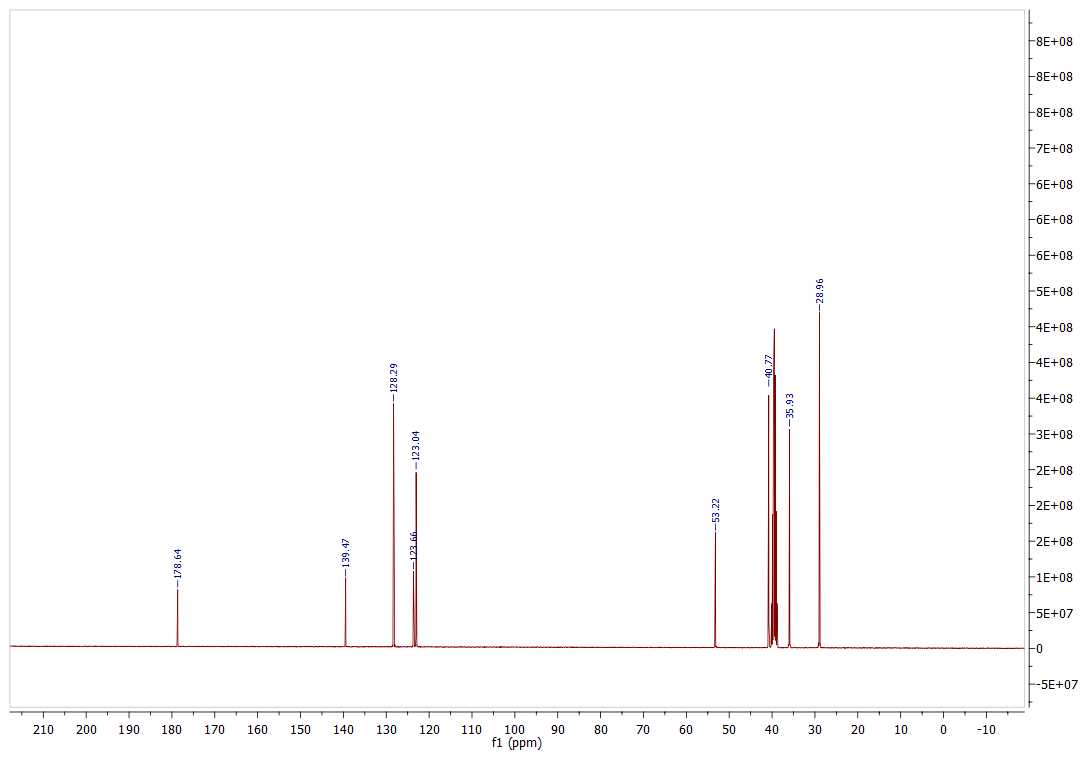


^13^C-NMR spectrum of **23**.

| ***Pseudomonas aeruginosa*** | 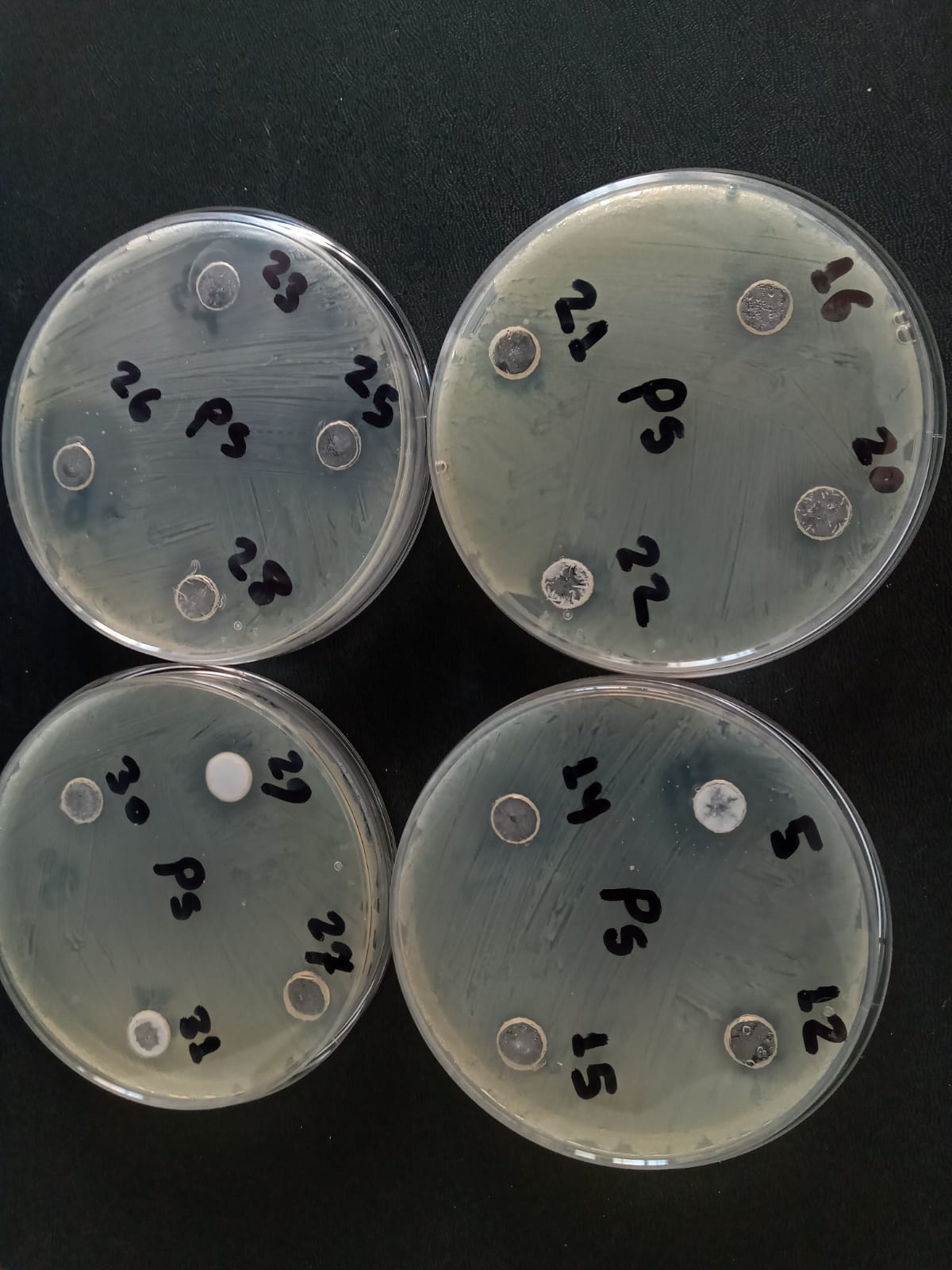 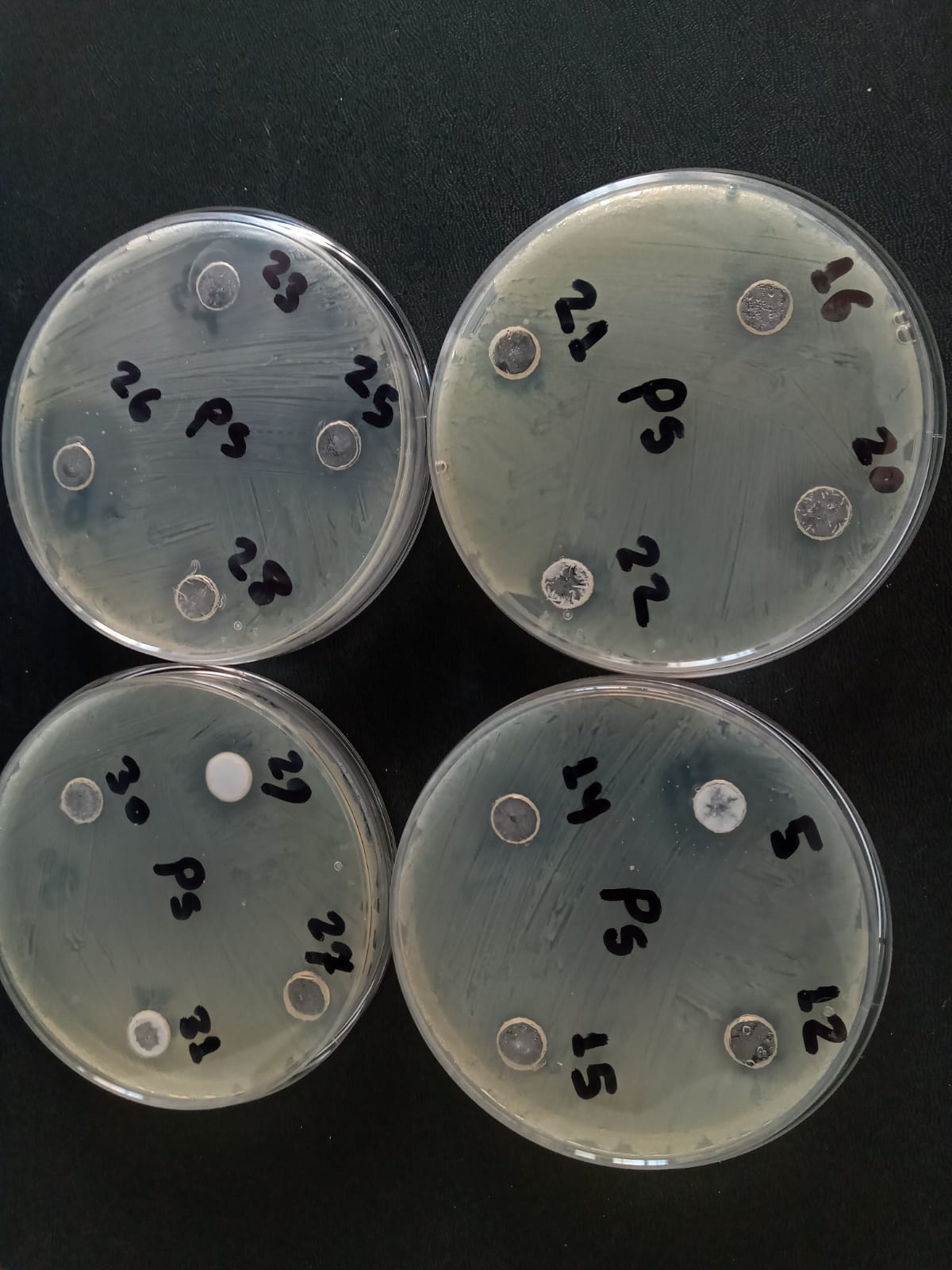  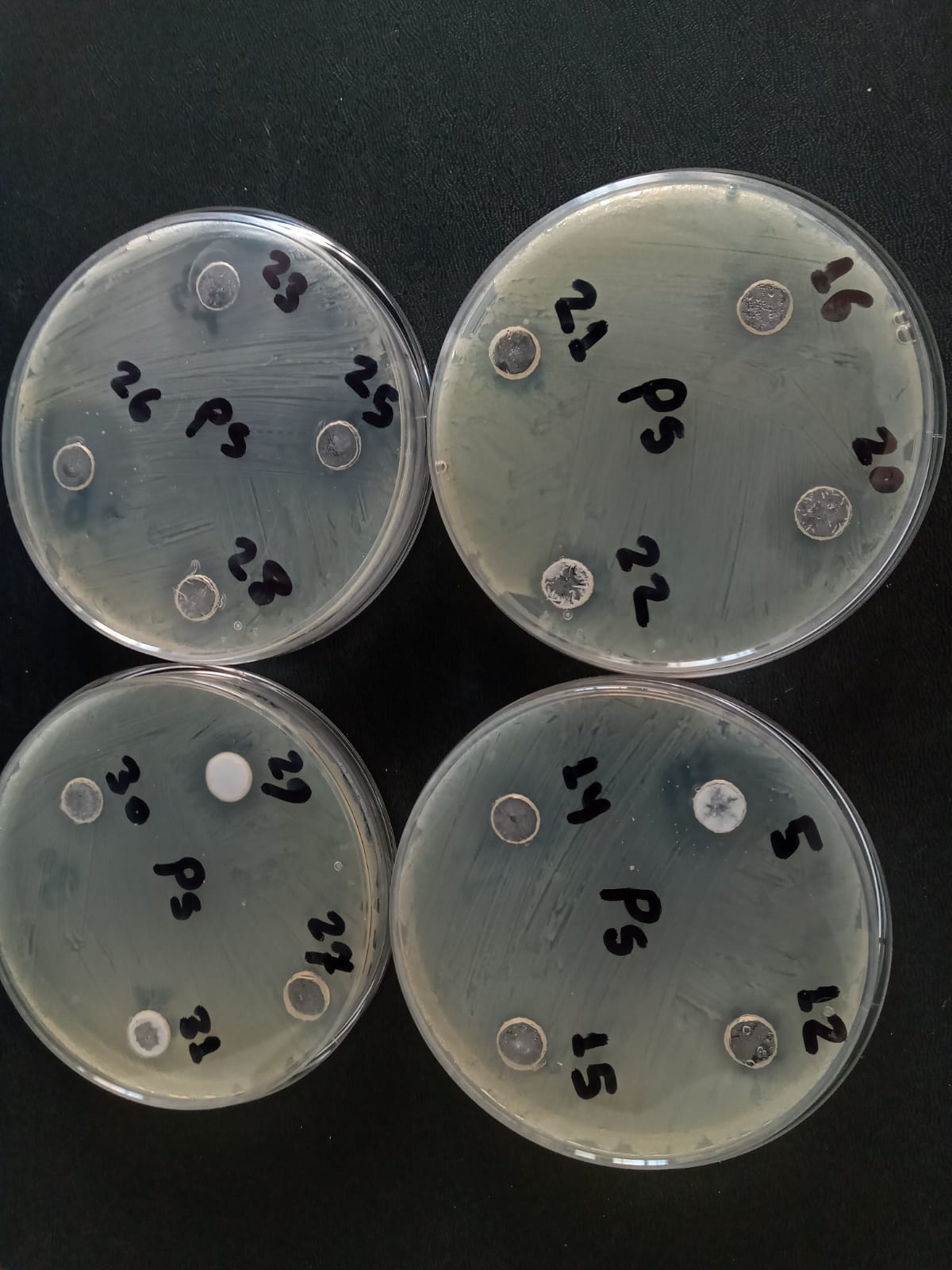 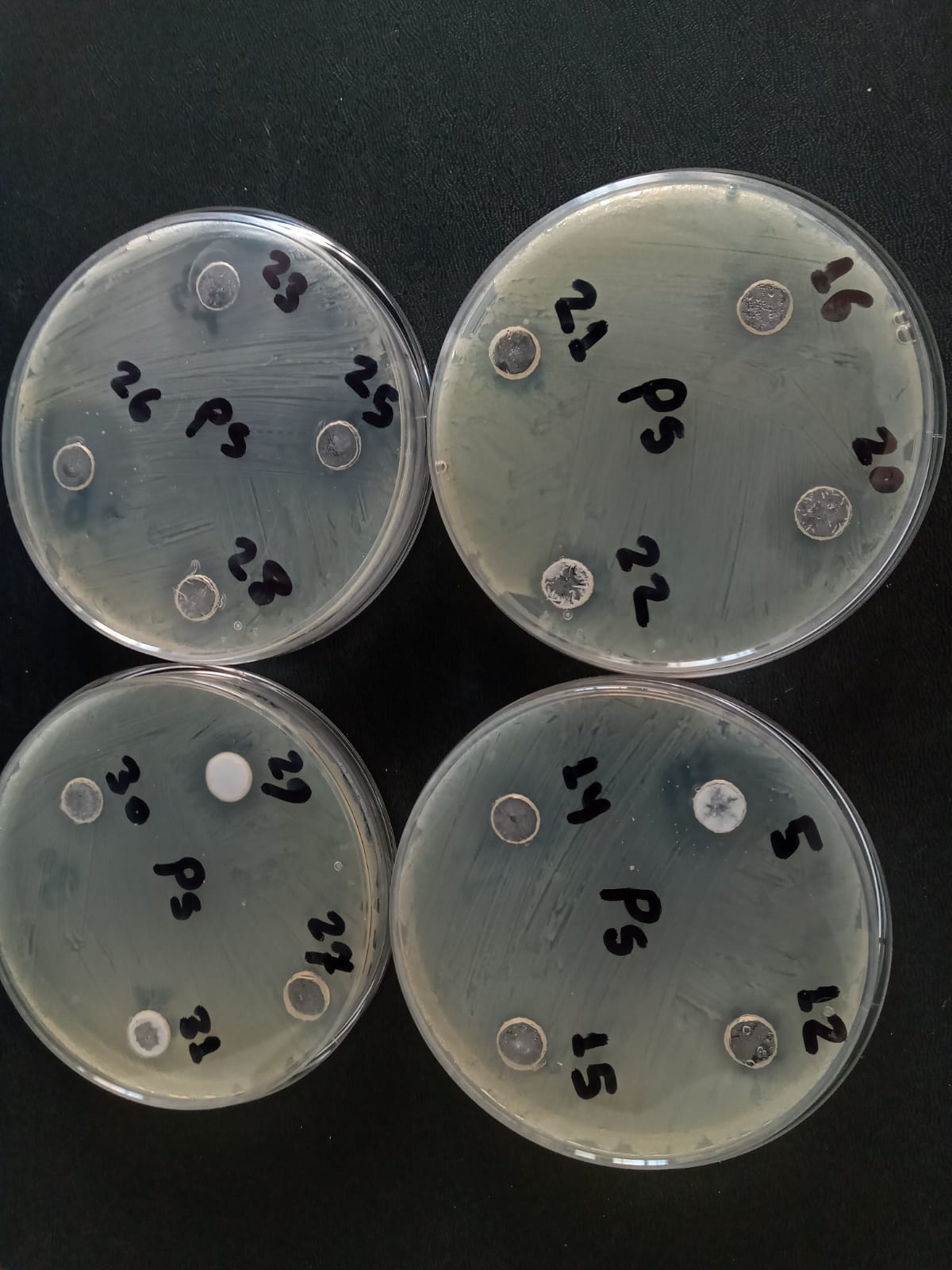 |
| --- | --- |
| ***Klebsiella pneumonia*** | 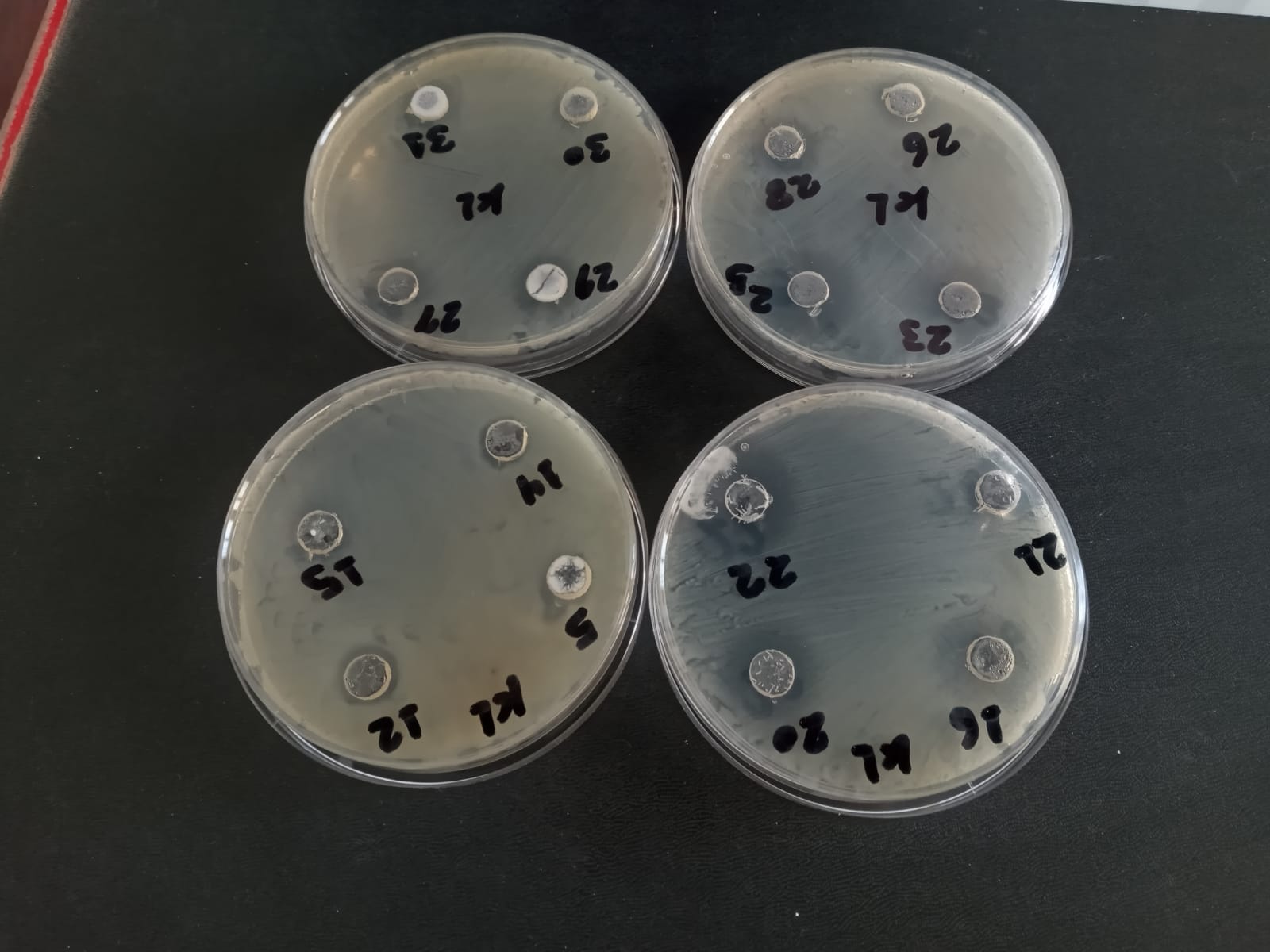 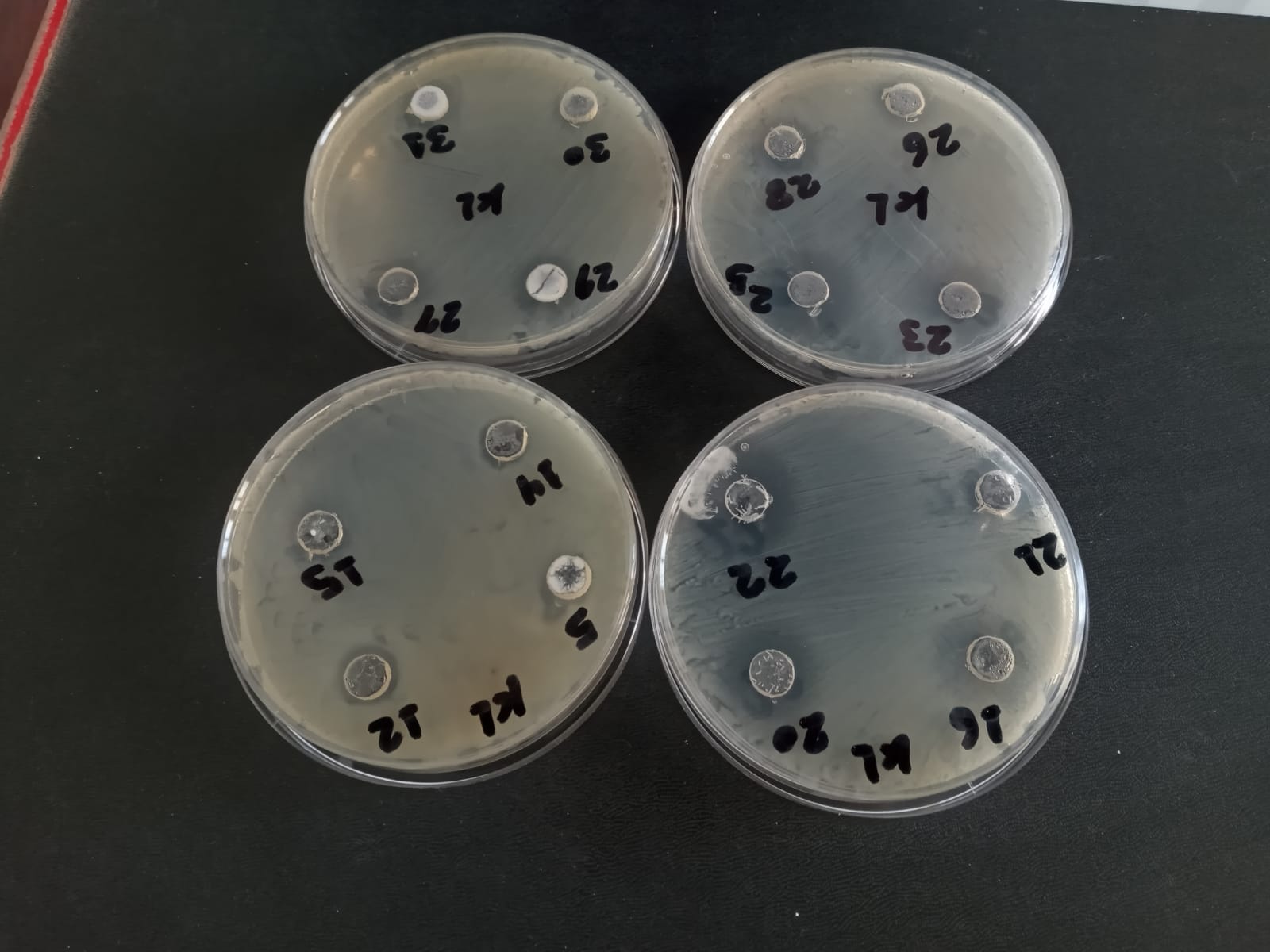  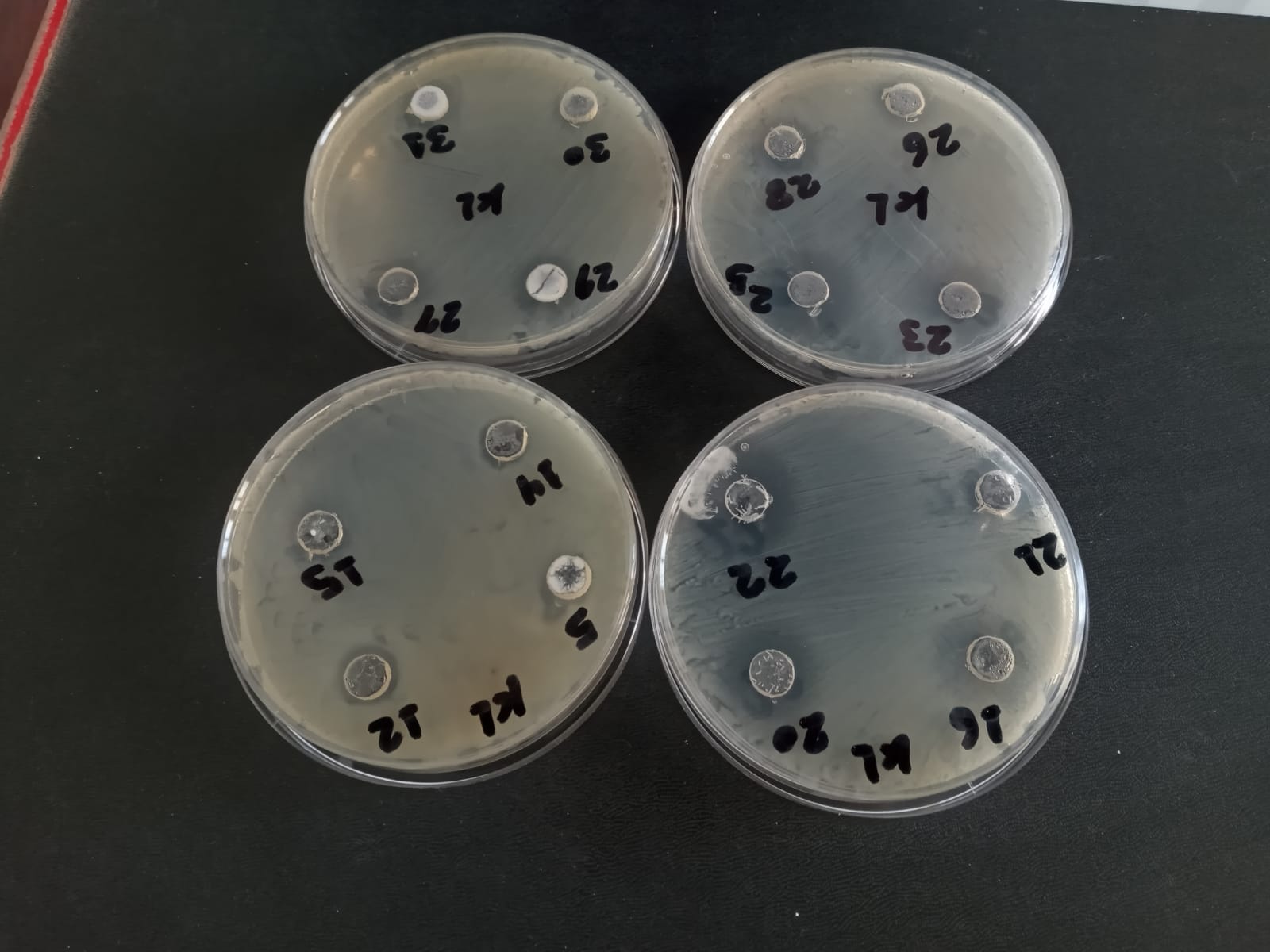 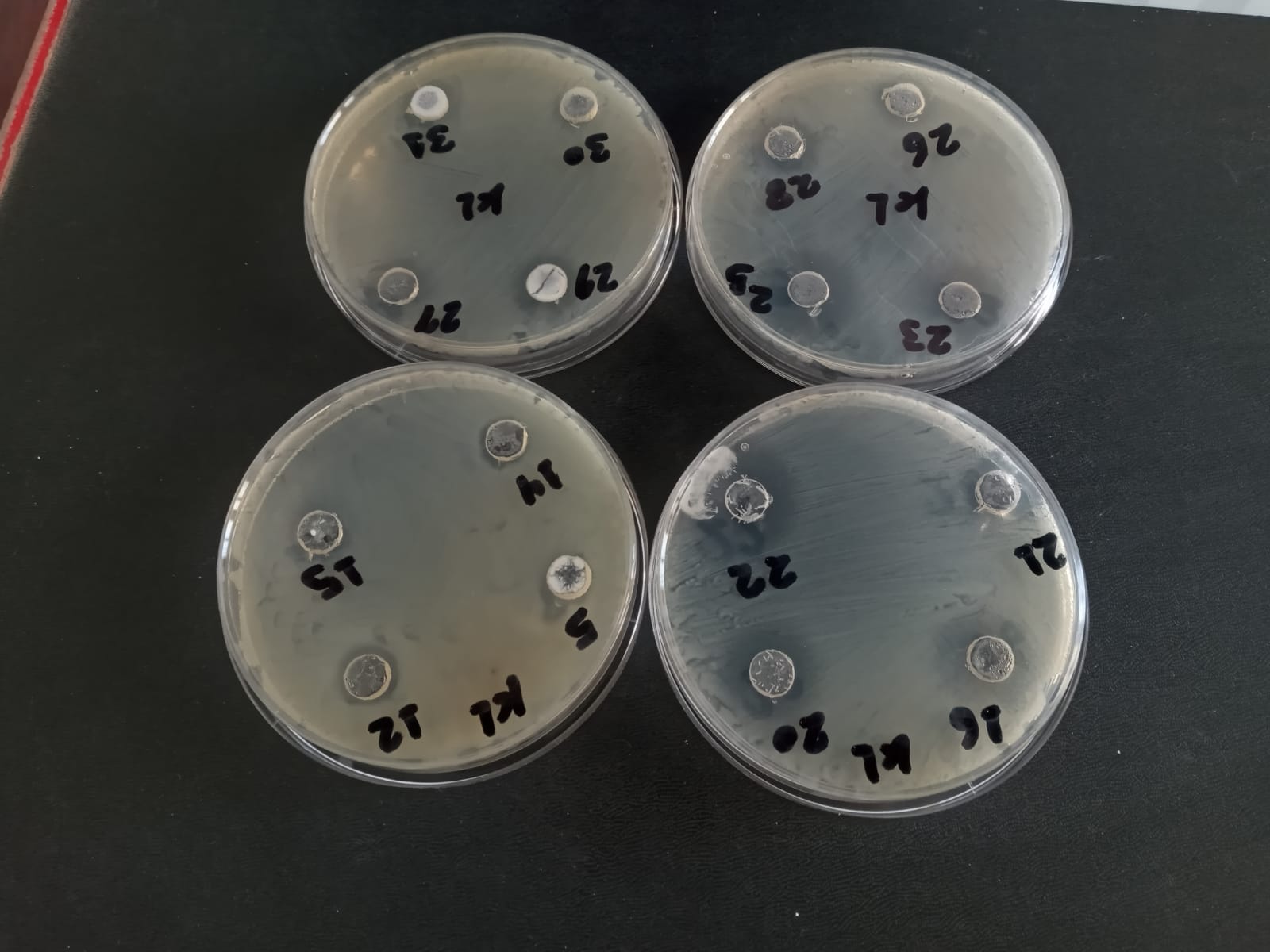 |
| ***Staphylococcus aureus*** | 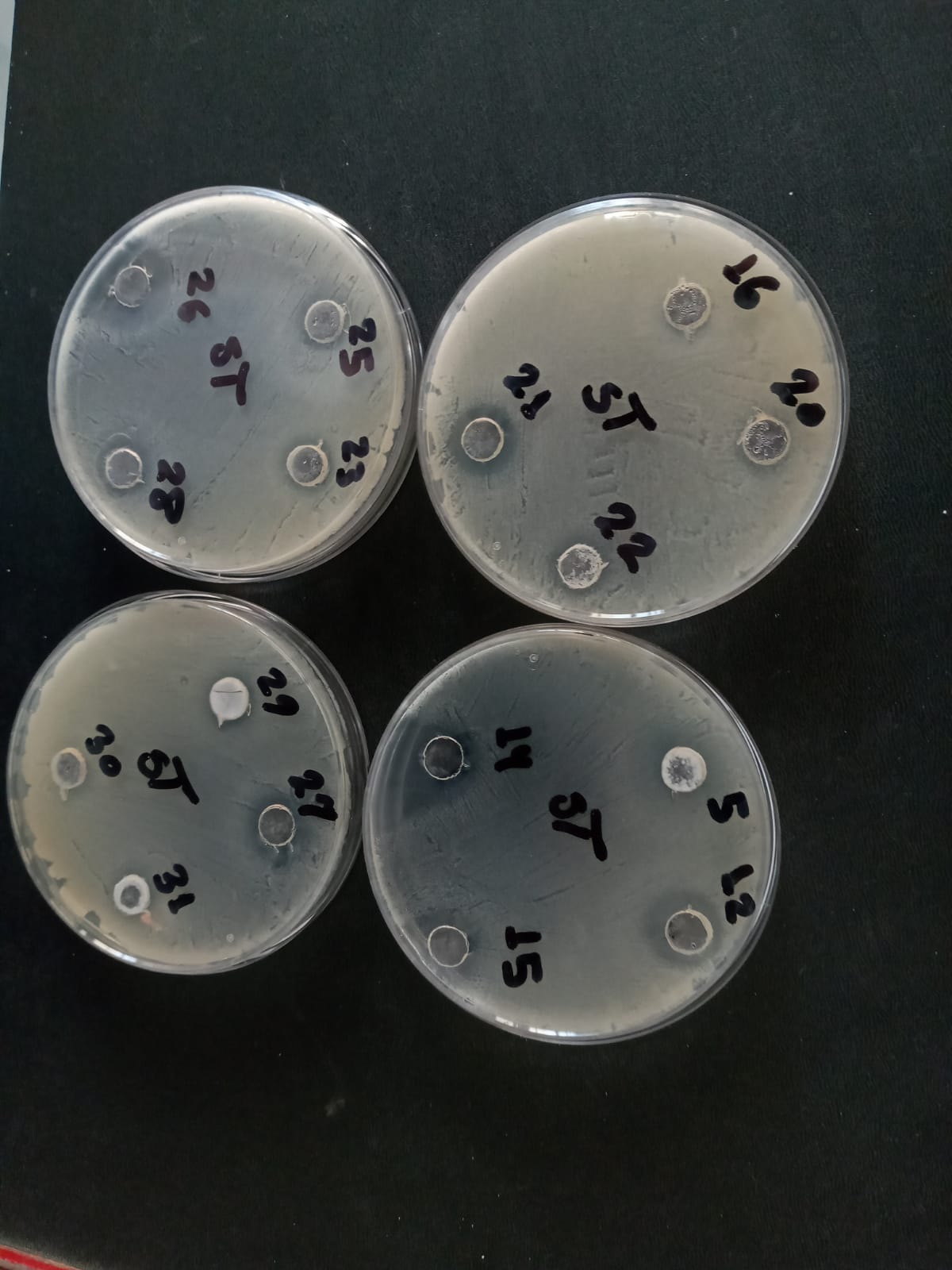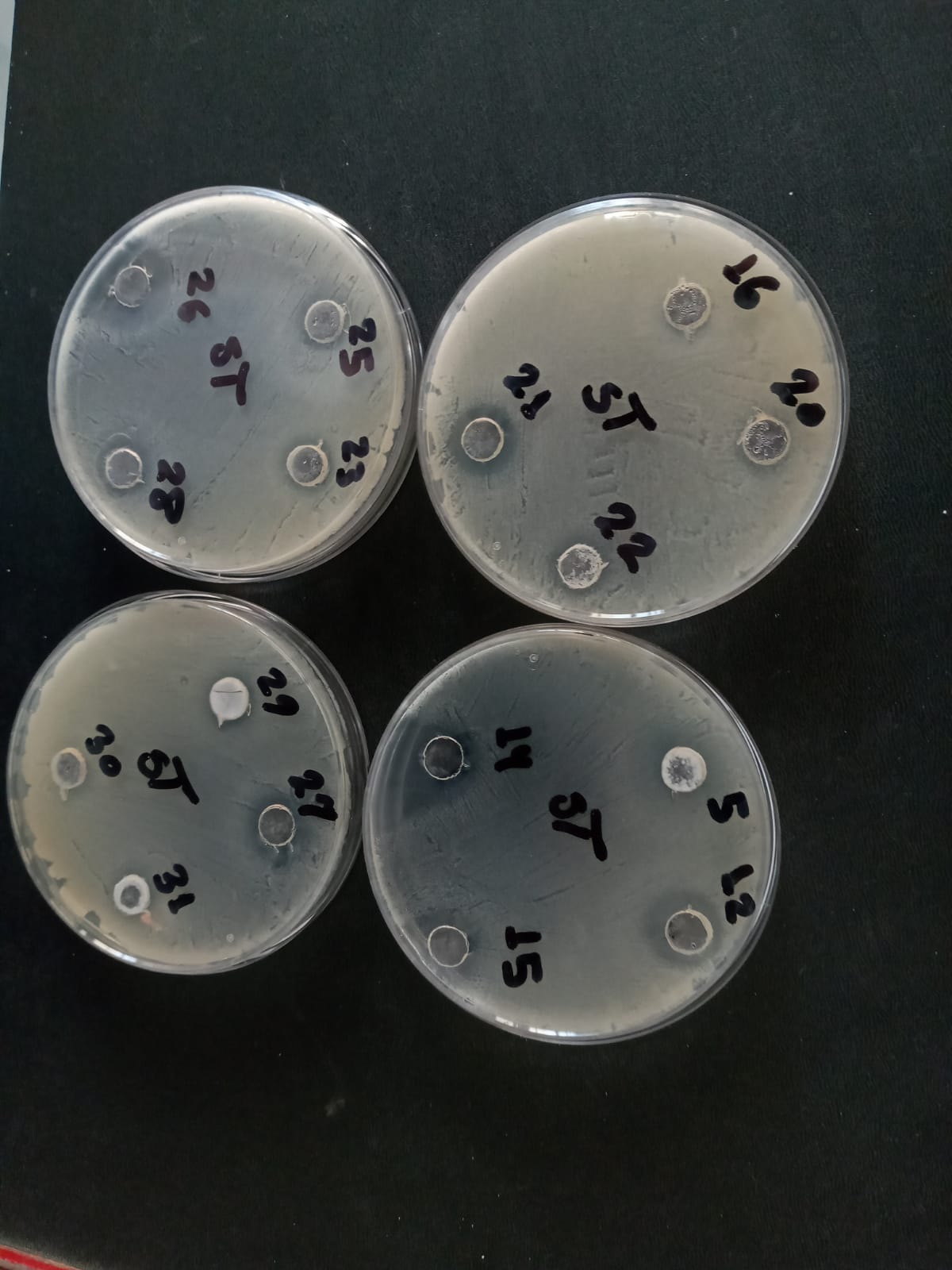  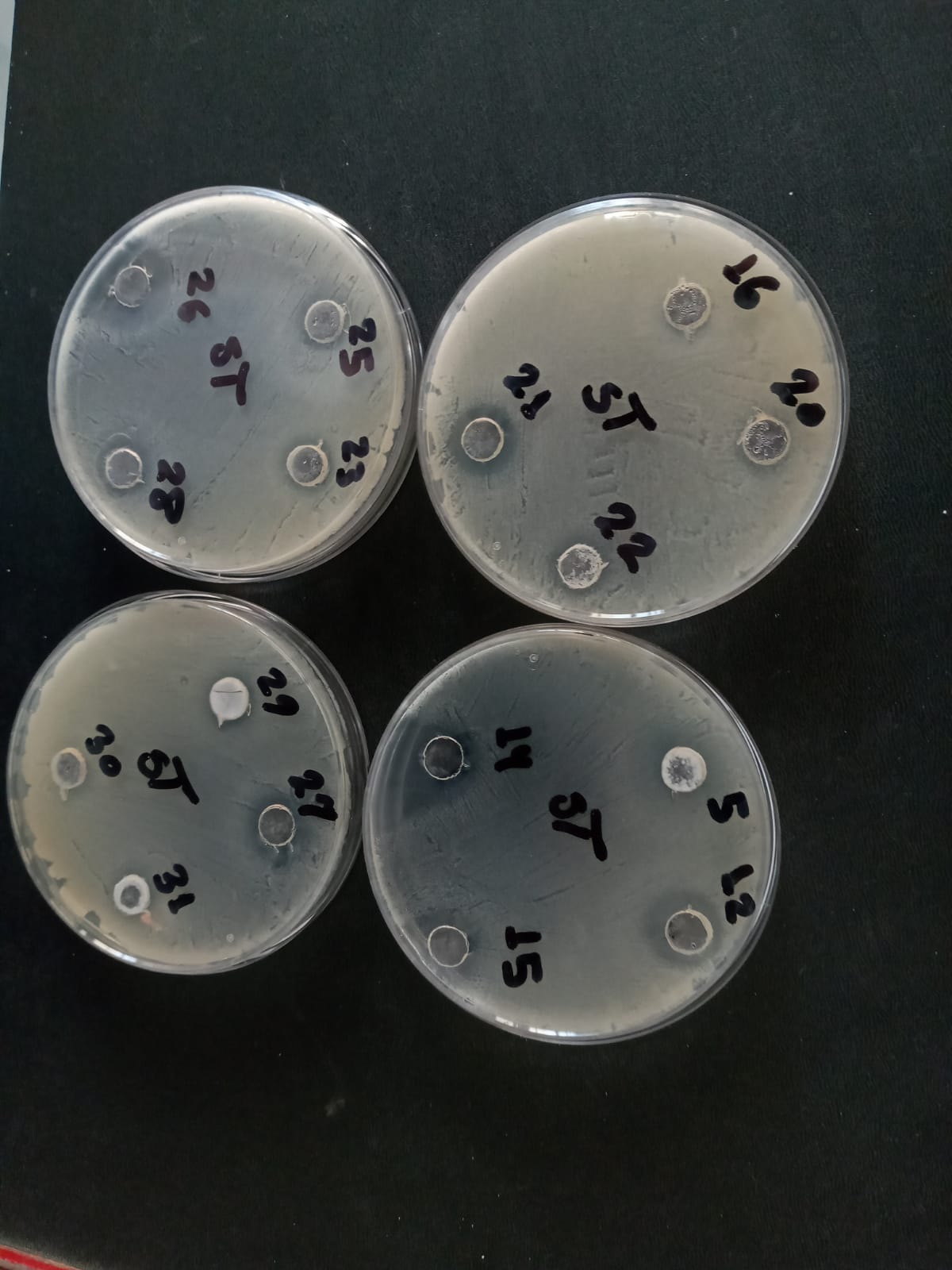 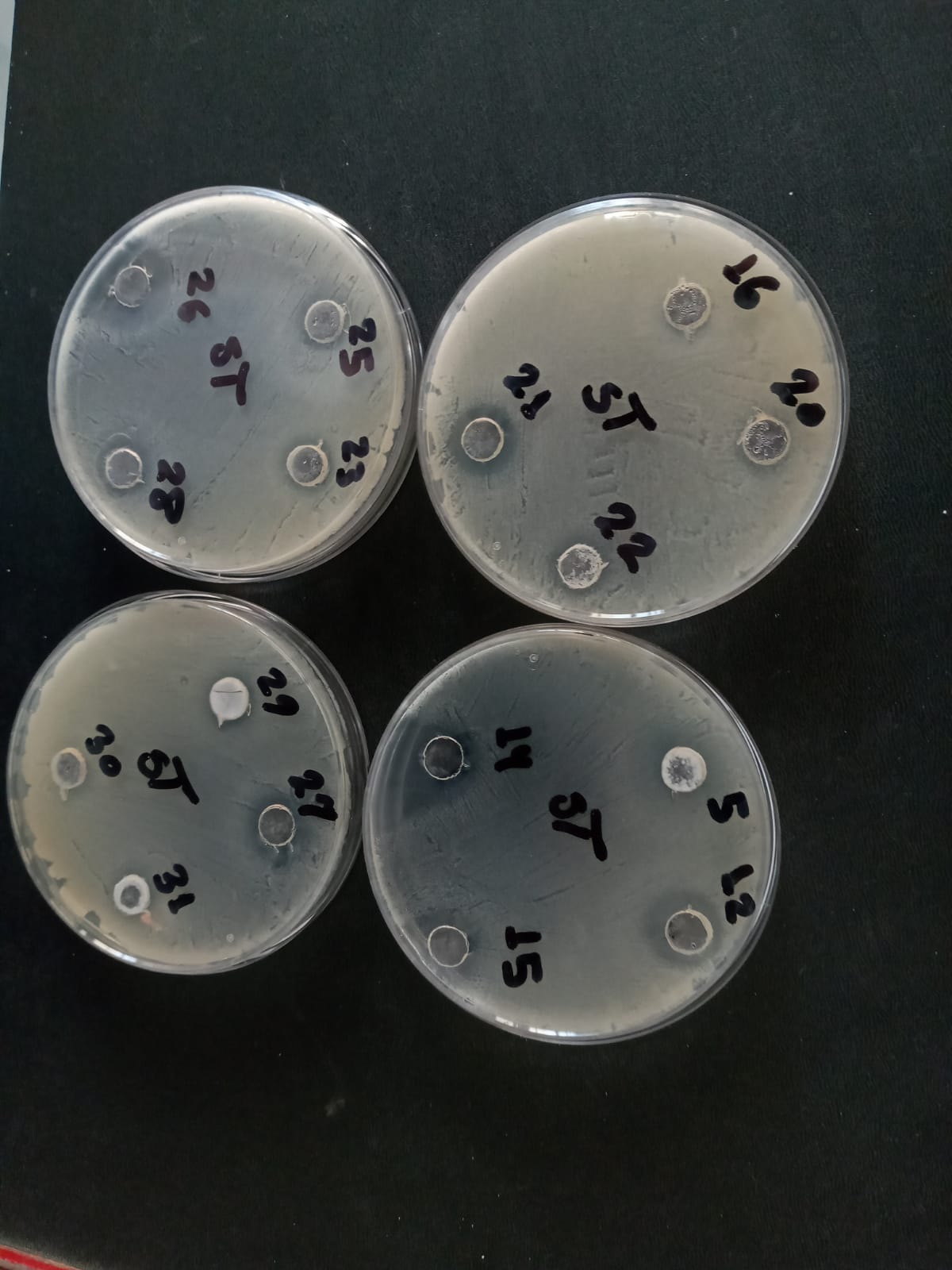 |
| ***Bacillus subtilis*** | 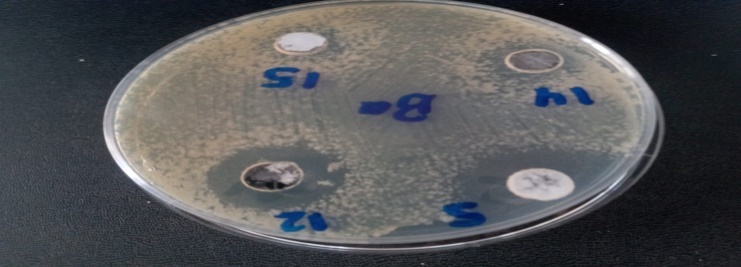 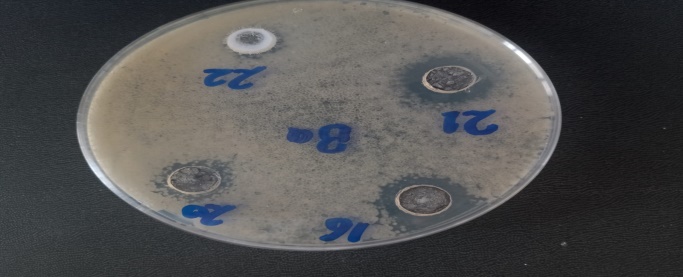  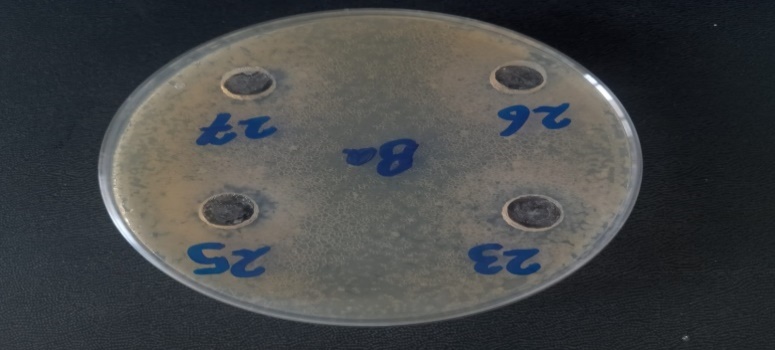 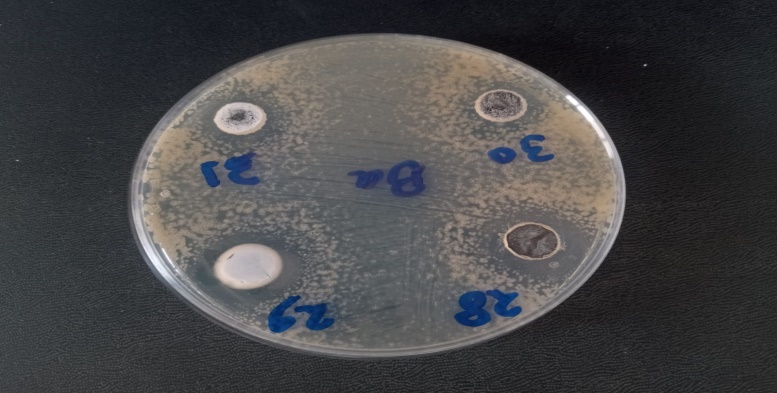 |
| ***Candida albicans*** | 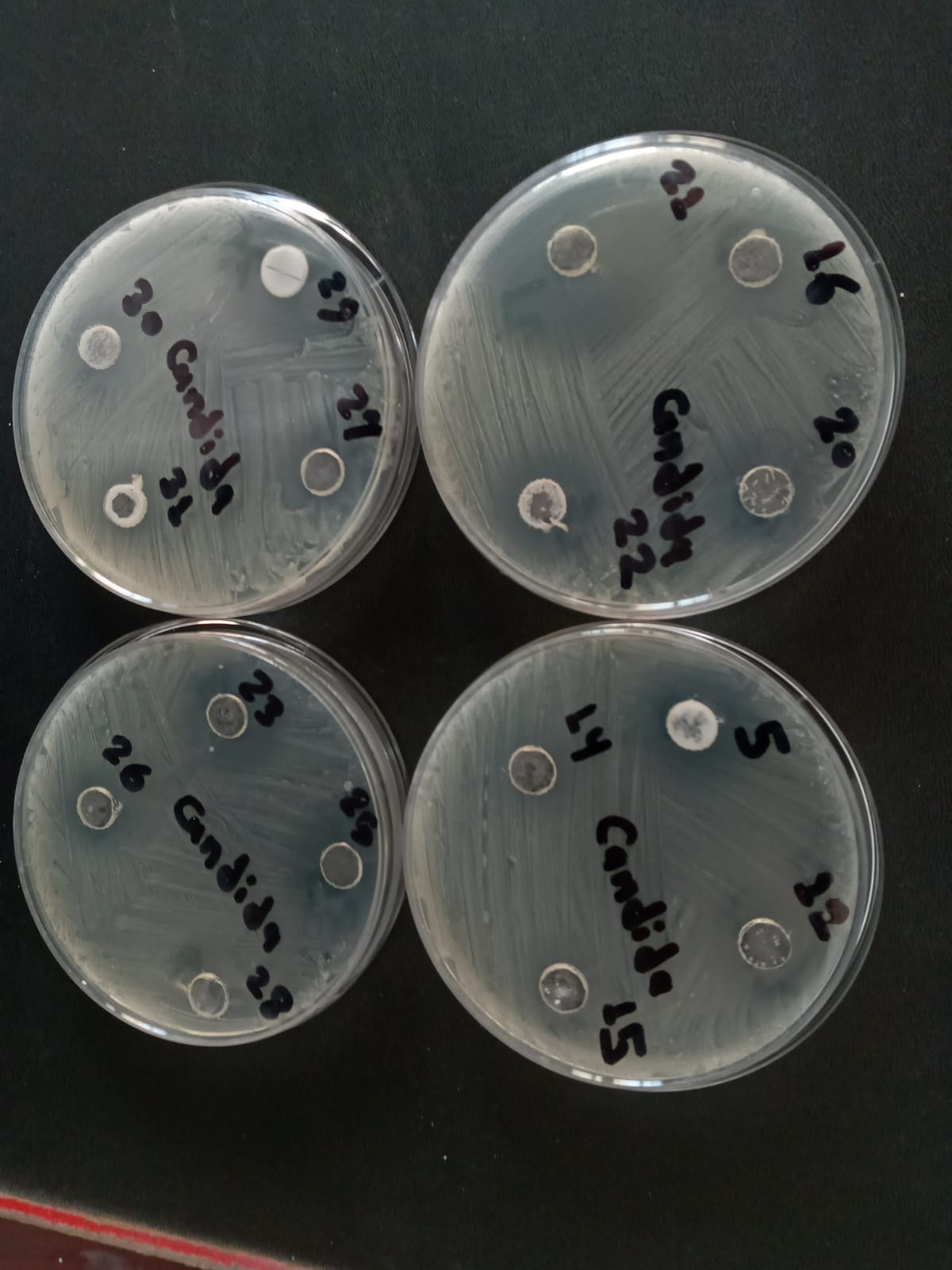 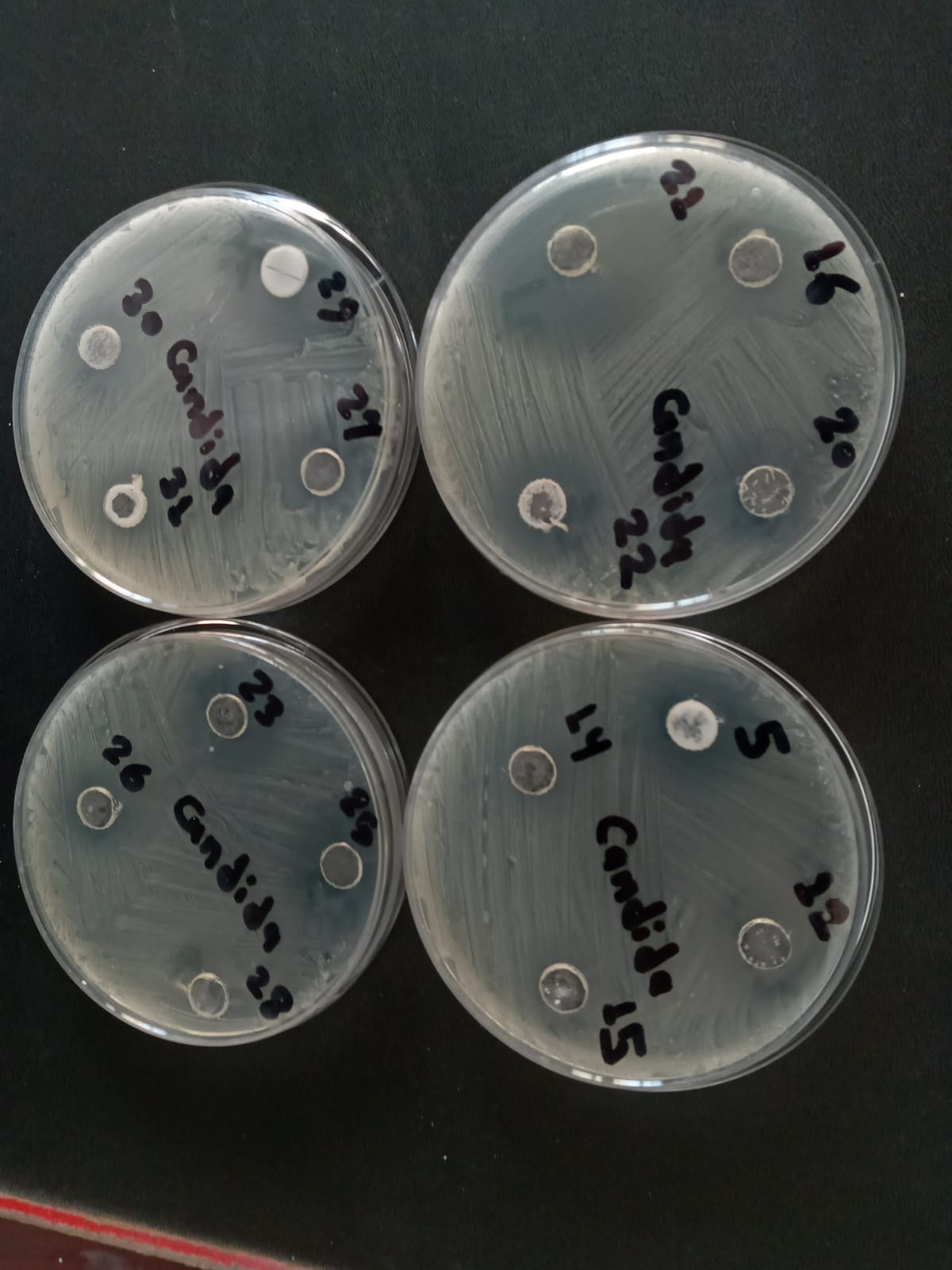  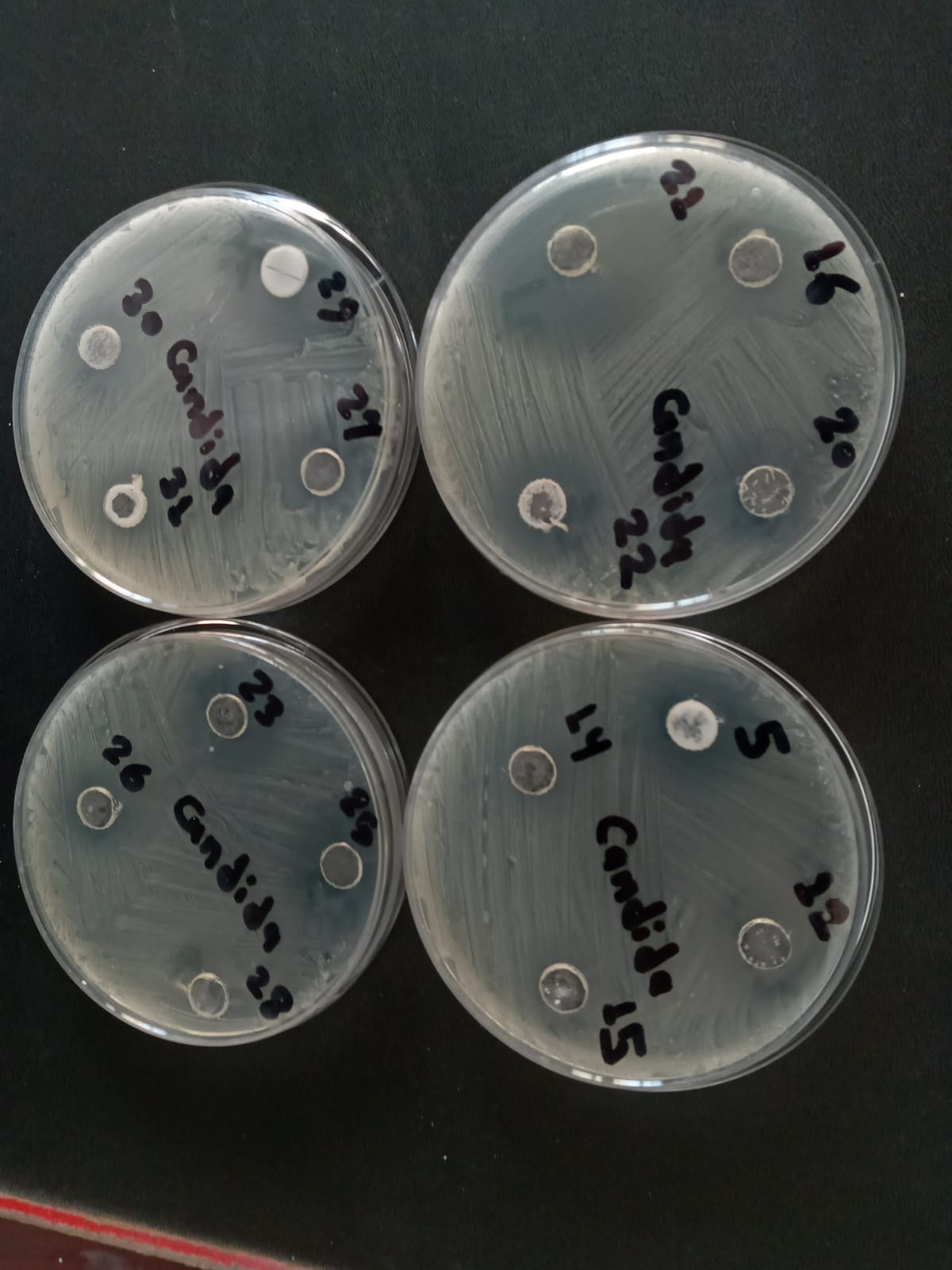 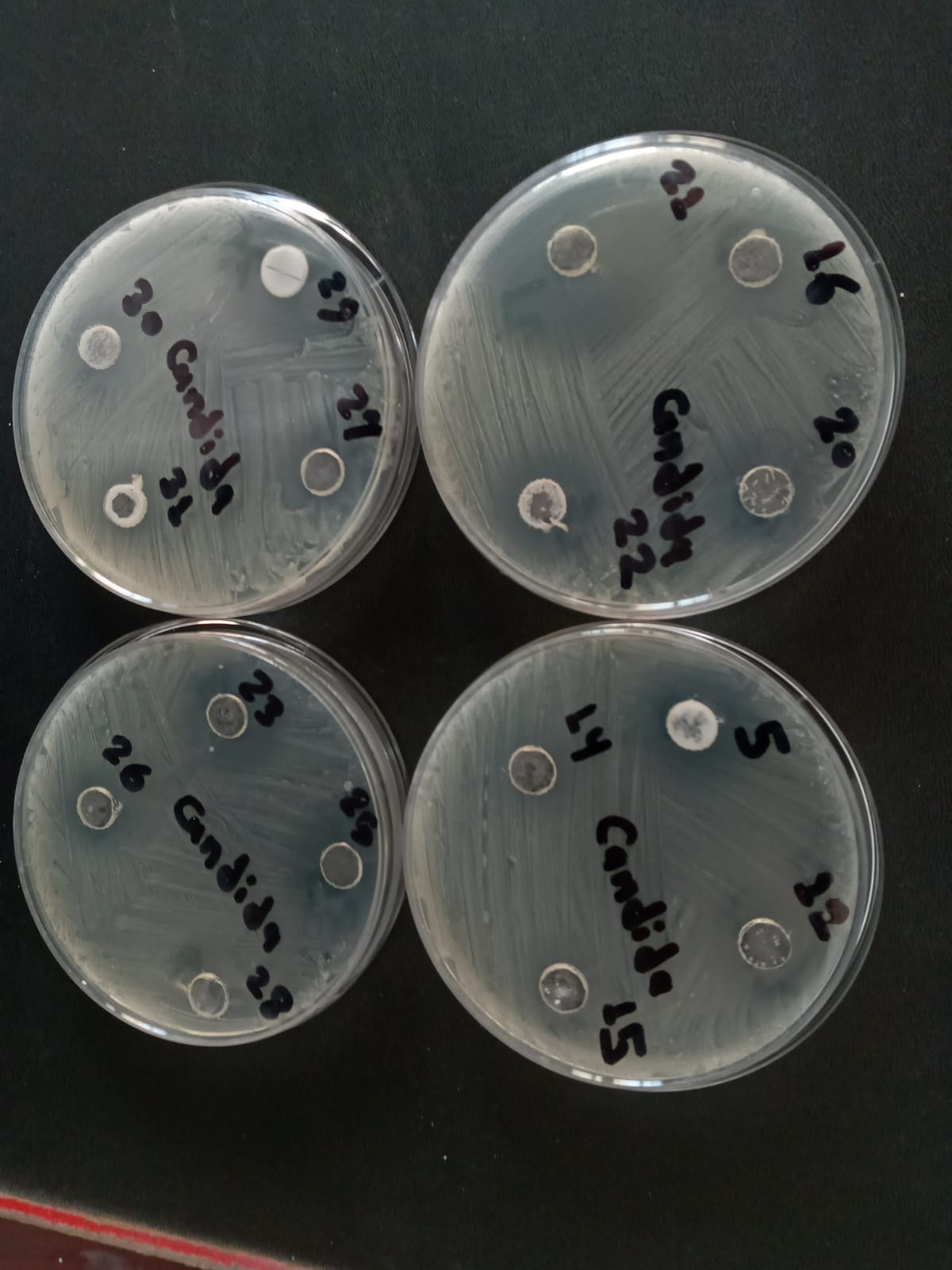 |
| ***Aspergillus fumagitus*** | 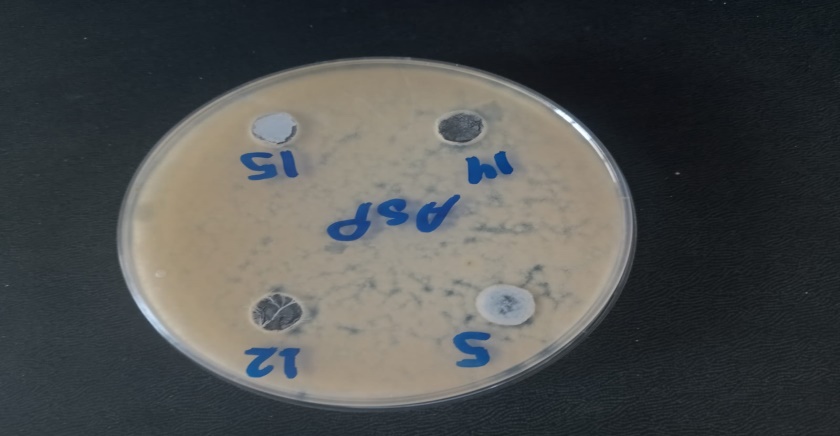 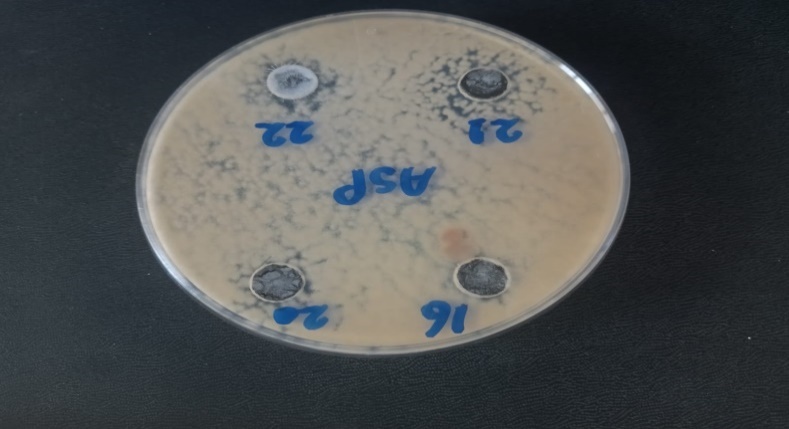  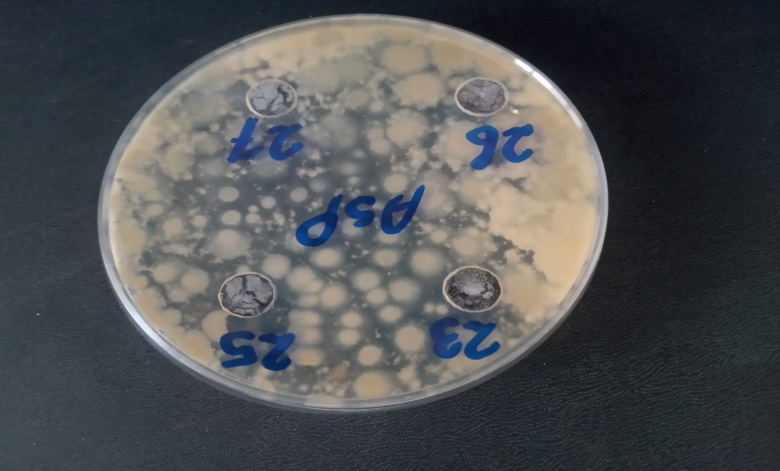 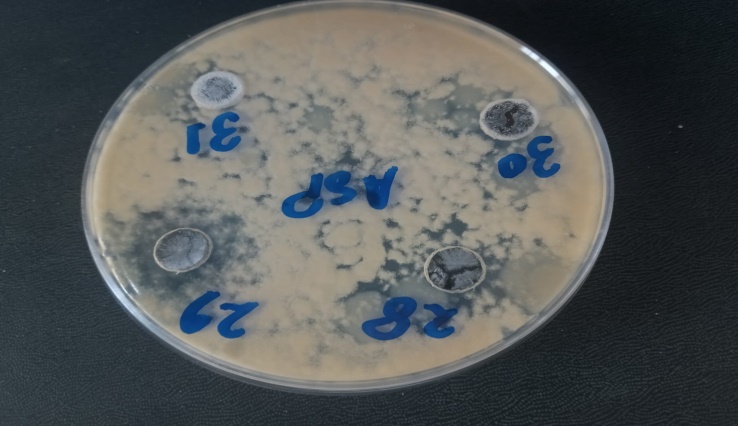 |

**Figure S1.** The antimicrobial activity evaluation of the designed unsymmetrical carbamide derivatives (**9-23**). (Compound **9 =** 12, Compound **10 =** 14, Compound **11 =** 15, Compound **12 =** 27, Compound **13 =** 05, Compound **14 =** 21, Compound **15 =** 23, Compound **16 =** 25, Compound **17 =** 28, Compound **18 =** 30, Compound **19 =** 22, Compound **20 =** 20, Compound **21=** 26 **=** 16, Compound **22 = 29**, Compound **23 =** 31).


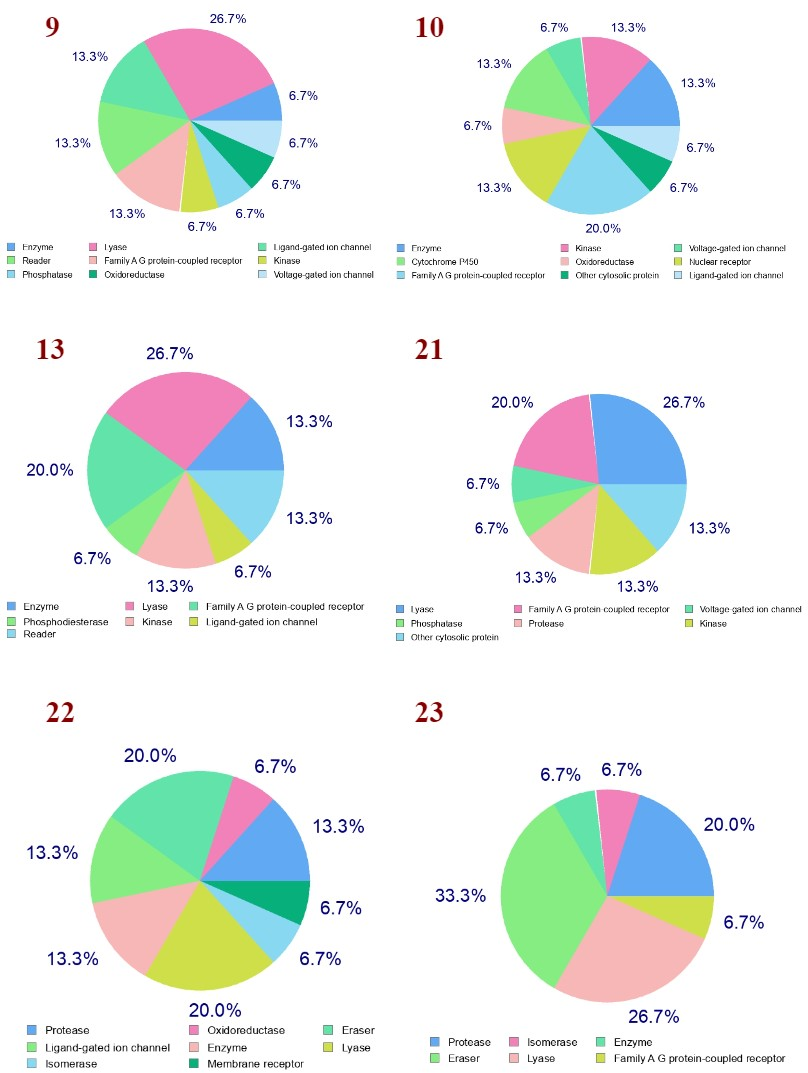


**Figure S2.** *In-silico* prediction of protein targets for Compounds (23, 22, 21, 10, and 13) using Swiss Target Predictions.


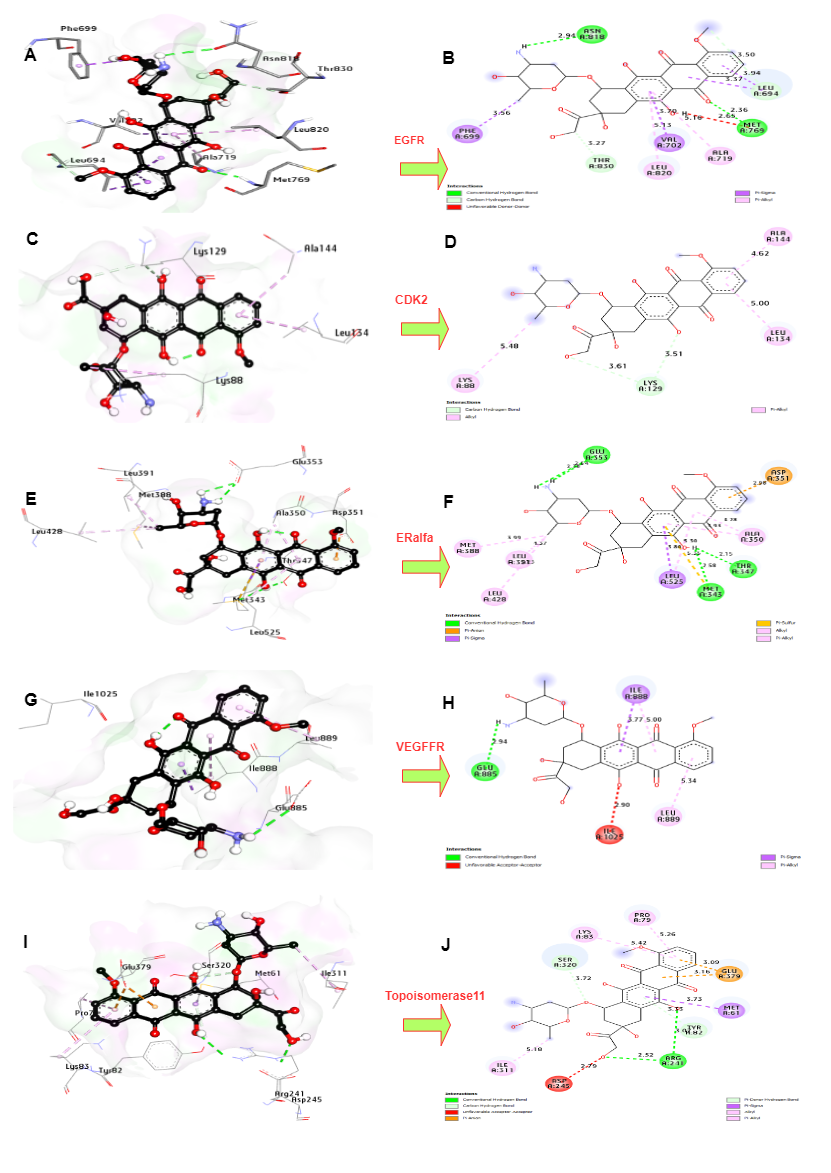


**Figure S3.** 2D-interactions of Doxorubicin at the binding pocket of anticancer protein receptors: (A and B) EGFR, (C and D) CDK2, (E and F) ERalfa, (G and H) VEGFFR, and (I and J) Topoisomerase II.

**Table S1.** *in-silico* prediction of protein targets for Compounds (23, 22, 21, 10, and 13) using PASS Target Predictions.

| **Compound 23** |  |  |
| --- | --- | --- |
| **Target name** | **Confidence** | **ChEMBL id** |
| Nuclear receptor subfamily 0 group B member 1 | 0.6552 | CHEMBL1795094 |
| Cannabinoid CB1 receptor | 0.5433 | CHEMBL218 |
| Cyclin-dependent kinase 13 | 0.6022 | CHEMBL1795192 |
| Cell division cycle 2-like protein kinase 6 | 0.5879 | CHEMBL6002 |
| Neuronal acetylcholine receptor protein alpha-7 subunit | 0.5527 | CHEMBL2492 |
| Nuclear receptor coactivator 1 | 0.4918 | CHEMBL1615387 |
| Epoxide hydratase | 0.482 | CHEMBL2409 |
| Glycine receptor subunit alpha 1 | 0.4703 | CHEMBL5845 |
| Vascular endothelial growth factor receptor 1 | 0.4145 | CHEMBL1868 |
| MAP kinase p38 gamma | 0.394 | CHEMBL4674 |
| Mitogen-activated protein kinase 15 | 0.3927 | CHEMBL5198 |
| **Compound 22** |  |  |
| **Target name** | **Confidence** | **ChEMBL id** |
| Nuclear receptor subfamily 0 group B member 1 | 0.6639 | CHEMBL1795094 |
| Serotonin 1e (5-HT1e) receptor | 0.4319 | CHEMBL2182 |
| Signal transducer and activator of transcription 1-alpha/beta | 0.4126 | CHEMBL6101 |
| Vascular endothelial growth factor receptor 1 | 0.4145 | CHEMBL1868 |
| Glycine receptor subunit alpha 1 | 0.4703 | CHEMBL5845 |
| Nuclear receptor coactivator 1 | 0.3482 | CHEMBL1615387 |
| MAP kinase p38 gamma | 0.394 | CHEMBL4674 |
| Mitogen-activated protein kinase 15 | 0.3927 | CHEMBL5198 |
| **Compound 21** |  |  |
| **Target name** | **Confidence** | **ChEMBL id** |
| Nuclear receptor subfamily 0 group B member 1 | 0.6465 | CHEMBL1795094 |
| Vascular endothelial growth factor receptor 1 | 0.503 | CHEMBL1868 |
| Serine/threonine-protein kinase PFTAIRE-2 | 0.7515 | CHEMBL5856 |
| Mitogen-activated protein kinase 13 | 0.7205 | CHEMBL1163124 |
| Cyclin-dependent kinase 2 | 0.7114 | CHEMBL3055 |
| Serine/threonine-protein kinase 2 | 0.6927 | CHEMBL4202 |
| Mitogen-activated protein kinase 1 | 0.6621 | CHEMBL3956 |
| Receptor-interacting serine/threonine-protein kinase 4 | 0.6471 | CHEMBL6083 |
| Mitogen-activated protein kinase 5 | 0.6418 | CHEMBL4852 |
| Cell division cycle 2-like protein kinase 6 | 0.5771 | CHEMBL6002 |
| **Compound 10** |  |  |
| **Target name** | **Confidence** | **ChEMBL id** |
| Mitogen-activated protein kinase 5 | 0.6418 | CHEMBL4852 |
| Cell division cycle 2-like protein kinase 6 | 0.5771 | CHEMBL6002 |
| Vascular endothelial growth factor receptor 1 | 0.4021 | CHEMBL1868 |
| Cyclin-dependent kinase-like 2 | 0.277 | CHEMBL5728 |
| Insulin-degrading enzyme | 0.2713 | CHEMBL1293287 |
| C-C chemokine receptor type 3 | 0.2635 | CHEMBL3473 |
| Aldo-keto-reductase family 1 member C3 | 0.1698 | CHEMBL4681 |
| Ribosomal protein S6 kinase alpha 5 | 0.1633 | CHEMBL4237 |
| **Compound 13** |  |  |
| **Target name** | **Confidence** | **ChEMBL id** |
| Vascular endothelial growth factor receptor 1 | 0.3824 | CHEMBL1868 |
| Cannabinoid CB1 receptor | 0.3423 | CHEMBL218 |
| Pregnane X receptor | 0.2998 | CHEMBL3401 |
| MCOLN3 protein | 0.2742 | CHEMBL1293243 |
| Sulfonylurea receptor 2, Kir6.2 | 0.2249 | CHEMBL2095198 |
| Nuclear receptor subfamily 0 group B member 1 | 0.2044 | CHEMBL1795094 |
| Glycine receptor subunit alpha 1 | 0.1789 | CHEMBL5845 |

**Table S2.** *In-silico* prediction of protein targets for Compounds (23, 22, 21, 10, and 13) using Swiss Target Predictions.

| **Target** | **Common name** | **UniProt ID** | **ChEMBL ID** | **Target Class** | **Probability*** |
| --- | --- | --- | --- | --- | --- |
| Epidermal growth factor receptor erbB1 | EGFR | P00533 | CHEMBL203 | Kinase | 0.112041901 |
| Receptor protein-tyrosine kinase erbB-2 | ERBB2 | P04626 | CHEMBL1824 | Kinase | 0.112041901 |
| Monoamine oxidase B | MAOB | P27338 | CHEMBL2039 | Oxidoreductase | 0.112041901 |
| PI3-kinase p110-alpha subunit | PIK3CA | P42336 | CHEMBL4005 | Enzyme | 0.112041901 |
| Estrogen receptor alpha | ESR1 | P03372 | CHEMBL206 | Nuclear receptor | 0.112041901 |
| Estrogen receptor beta | ESR2 | Q92731 | CHEMBL242 | Nuclear receptor | 0.112041901 |
| Cyclooxygenase-2 | PTGS2 | P35354 | CHEMBL230 | Oxidoreductase | 0.112041901 |
| Protein-tyrosine phosphatase 1B | PTPN1 | P18031 | CHEMBL335 | Phosphatase | 0.112041901 |
| Adenosine A1 receptor | ADORA1 | P30542 | CHEMBL226 | Family A G protein-coupled receptor | 0.112041901 |
| Dopamine transporter | SLC6A3 | Q01959 | CHEMBL238 | Electrochemical transporter | 0.112041901 |
| Adenosine A2a receptor | ADORA2A | P29274 | CHEMBL251 | Family A G protein-coupled receptor | 0.112041901 |
| Adenosine A3 receptor | ADORA3 | P0DMS8 | CHEMBL256 | Family A G protein-coupled receptor | 0.112041901 |
| Androgen Receptor | AR | P10275 | CHEMBL1871 | Nuclear receptor | 0.112041901 |
| Carbonic anhydrase II | CA2 | P00918 | CHEMBL205 | Lyase | 0.086442693 |
| Vascular endothelial growth factor receptor 1 | FLT1 | P17948 | CHEMBL1868 | Kinase | 0.060424588 |
| Cyclin-dependent kinase 1/cyclin B | CDK1 | Q8WWL7 | CHEMBL2094127 | Other cytosolic protein | 0.060424588 |
| Cyclin-dependent kinase 2/cyclin A | CDK2 | P24941 | CHEMBL2094128 | Other cytosolic protein | 0.060424588 |
| CDK6/cyclin D1 | CDK6 | P24385 | CHEMBL2111455 | Kinase | 0.060424588 |
| Cyclin-dependent kinase 6 | CDK6 | Q00534 | CHEMBL2508 | Kinase | 0.060424588 |
| Tyrosine-protein kinase LCK | LCK | P06239 | CHEMBL258 | Kinase | 0.060424588 |
| Cyclin-dependent kinase 2 | CDK2 | P24941 | CHEMBL301 | Kinase | 0.060424588 |
| Cyclin-dependent kinase 1 | CDK1 | P06493 | CHEMBL308 | Kinase | 0.060424588 |
| Cyclin-dependent kinase 4 | CDK4 | P11802 | CHEMBL331 | Kinase | 0.060424588 |

**Table S3.** *In-silico* prediction of protein targets for Compounds (23, 22, 21, 10, and 13) using Pharm Mapper Predictions.

| **Target Name** | **Class** | **UniProt** | **Number of Features** | **Fit Score** | **Normalized Fit Scor** | **Z-score** |
| --- | --- | --- | --- | --- | --- | --- |
| Cell division protein kinase 2 | None | P24941 | 3 | 2.788 | 0.9292 | 0.474672 |
| Epidermal growth factor receptor EGFR | None | P00533 | 3 | 2.765 | 0.9218 | 0.323427 |
| Mitogen-activated protein kinase 14 | None | Q16539 | 5 | 4.603 | 0.9207 | 2.63541 |
| Leukotriene A-4 hydrolase | Hydrolase | LKHA4 | 3 | 2.693 | 0.8978 | 0.477053 |
| Methionine aminopeptidase 2 | Hydrolase | AMPM2 | 5 | 4.418 | 0.8835 | 2.74084 |
| Vascular endothelial growth factor receptor 2 | None | P35968 | 5 | 4.416 | 0.8832 | 2.44322 |
| Cyclin-A2 | Protein Kinase | CCNA2 | 3 | 2.607 | 0.869 | 0.235819 |
| Heat shock protein HSP 90-alpha | Chaperone | HS90A | 4 | 3.343 | 0.8358 | 1.08613 |
| Amine oxidase [flavin-containing] B | Oxidoreductase | AOFB | 3 | 2.395 | 0.7983 | 0.114237 |
| Androgen receptor | Transcription | ANDR | 5 | 3.852 | 0.7703 | 1.70939 |
| Urokinase-type plasminogen activator | Hydrolase | UROK | 4 | 2.923 | 0.7306 | 0.541714 |
| Serine/threonine-protein kinase Chk1 | Transferase | CHK1 | 5 | 3.651 | 0.7302 | 1.07684 |
| B-Raf proto-oncogene serine/threonine-protein kinase | Kinase | BRAF1 | 7 | 5.1 | 0.7286 | 2.61428 |
| Kinesin-like protein KIF11 | Cell Cycle | KIF11 | 5 | 3.608 | 0.7215 | 1.33755 |
| Basic fibroblast growth factor receptor 1 | Protein Kinase | FGFR1 | 4 | 2.87 | 0.7176 | 0.267598 |
| Carbonic anhydrase 2 | Lyase | CAH2 | 5 | 3.538 | 0.7076 | 0.713347 |
| Phenyl ethanolamine N-methyltransferase | Transferase | PNMT | 5 | 3.53 | 0.706 | 1.16641 |
| z'score is a score generated from the molecule's fit score and a library score matrix calculated beforehand | | | | | | |

**Table S4.** Molecular interactions of ligands with amino acids of FDC1 protein of *A. fumigates* (PDB: ID 4ZA5)

| **Protein** | **Ligand** | **3D Structure** | **Hydrophilic Interactions** | | **Hydrophobic Contacts** | | **No. of**  **H-Bonds** | **No. of**  **Total Bonds** | **affinity**  **kcal mol-1** |
| --- | --- | --- | --- | --- | --- | --- | --- | --- | --- |
|  |  |  | **Residue (H- Bond)** | **Length** | **Residue (Bond type)** | **Length** |  |  |  |
| **FDC1 protein of *A. fumigates*** | **23** |  | Ile171, (H- Bond) | 2.27 | Ala172, (Pi-alkyl)  Arg173, (Pi-alkyl)  Leu185, (Pi-alkyl)  Leu439, (Pi-alkyl)  Met326, (Pi-Sulfur)  Ile327, (Pi-alkyl) | 3.77  5.13  4.86  5.44  5.48  5.41 | **1** | **12** | **-11.90** |
|  | **10** |  | Ser224, (H- Bond)  Gln190, (H- Bond)  Gln190, (H- Bond) | 2.49  2.52  5.74 | Ala331, (Pi-alkyl)  Ile171, (Pi-alkyl)  Ile327, (Pi-alkyl)  Met225, (Pi-T-shaped)  Pro226, (Pi-cation)  Ser224, (carbon-Bond) | 3.88  4.12  4.49  5.34  4.65  3.05 | **3** | **11** | **-11.30** |
|  | **21** |  | Ile171, (H- Bond) | 1.93 | Ile327, (Pi-sigma)  Ile171, (Pi-sigma)  Ser223, (carbon-Bond)  Ser224, (carbon-Bond) | 3.55  3.83  5.86  3.17 | **1** | **6** | **-10.10** |
|  | **Miconazole** | 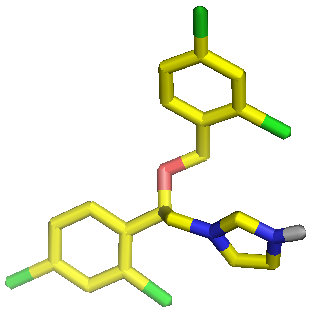 | Met225, (H- Bond) | 5.30 | Ala331, (Pi-alkyl)  Ala392, (halogen bond)  Ile171, (Pi-alkyl)  Ile327, (Pi-alkyl)  Ile327, (Pi-sigma)  Met225, (amide-T-shaped)  Pro226, (Pi-cation)  Ser223, (carbon-Bond) | 5.08  3.03  3.70  4.33  4.60  5.30  4.65  5.30 | **1** | **12** | **-11.00** |

**Table S5.** Molecular interactions of ligands with amino acids of the Sterol 14-demethylase of *C. albicans* (PDB: ID 5TZ1)

| No | **Protein** | **Ligand** | **3D Structure** | **Hydrophilic Interactions** | | **Hydrophobic Contacts** | | **No. of**  **H-Bonds** | **No. of**  **Total Bonds** | **affinity**  **kcal mol-1** |
| --- | --- | --- | --- | --- | --- | --- | --- | --- | --- | --- |
|  |  |  |  | **Residue (H- Bond)** | **Length** | **Residue (Bond type)** | **Length** |  |  |  |
| 1 | **Sterol 14-demethylase** **of *C. albicans*** | **12** |  | Ile471, (H- Bond) | 1.94 | Ile131, (Pi-alkyl)  Leu139, (Pi-alkyl)  Lys143, (Pi-alkyl)  Ile300, (Pi-alkyl)  Cys470,(C-Hydrogen bond)  Ile379, (Pi-alkyl) | 3.92  4.76  4.33  5.01  2.74  5.04 | **1** | **9** | **-8.50** |
| 2 |  | **13** |  | Lys143, (H- Bond)  Tyr132, (H- Bond) | 2.17  2.76 | Ala146, (Pi-alkyl)  Ile379, (Pi-alkyl)  Leu139, (Pi-alkyl)  Ile131, (Pi-alkyl)  Ile471, (Pi-alkyl)  Ile304, (Pi-alkyl)  Leu376, (Pi-alkyl)  Leu300, (Pi-alkyl) | 4.01  4.12  5.13  5.35  5.23  4.16  4.53  4.16 | **2** | **14** | **-9.10** |
| 3 |  | **23** |  | - | 1.84 | Cys470, (Pi-sulfur)  Ile379, (Pi-alkyl)  Thr311, (Pi-sigma)  Ala476, (Pi-alkyl)  Pro375, (Pi-alkyl)  Leu376, (Pi-alkyl)  Phe463, (C-Hydrogen) | 5.45  5.04  5.04  4.77  4.89  5.36  3.61 | **0** | **9** | **-8.30** |
| 4 |  | **Miconazole** | 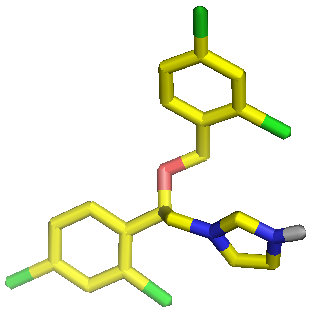 | Ile471, (H- Bond) | 2.09 | Lys143, (Pi-alkyl)  Lys143, (Pi-alkyl)  Phe475, (Pi- alkyl)  Ile304, (Pi-alkyl)  Leu139, (Pi-alkyl)  Cys470, (Pi-alkyl)  Ile131, (Pi-alkyl)  Ile304, (Pi-alkyl)  Leu300, (Pi-alkyl) | 4.74  4.44  4.16  4.90  4.74  4.80  3.72  4.90  5.14 | **0** | **14** | **-8.00** |

**Table S6.** Molecular interactions of ligands with amino acids of Dihydropteroate synthase of *S. aureus* (PDB: ID 1AD4)

| **Protein** | **Ligand** | **3D Structure** | **Hydrophilic Interactions** | | **Hydrophobic Contacts** | | **No. of**  **H-Bonds** | **No. of**  **Total Bonds** | **affinity**  **kcal mol-1** |
| --- | --- | --- | --- | --- | --- | --- | --- | --- | --- |
|  |  |  | **Residue (H- Bond)** | **Length** | **Residue (Bond type)** | **Length** |  |  |  |
| **Dihydropteroate synthase of *S. aureus*** | **10** |  | Arg52, (H- Bond)  Gln105, (H- Bond) | 2.70  2.67 | Arg202, (Pi- alkyl)  Arg202, (Pi- alkyl)  His241, (Pi-sigma)  His241, (Pi-Pi stacked  Pro216, (Pi-alkyl)  Phe172, (Pi-alkyl)  Lys203, (Carbon H- Bond) | 4.41  5.25  3.97  4.96  4.42  4.96  3.63 | **2** | **10** | **--6.20** |
|  | **9** |  | Arg52, (H- Bond)  Ser50, (H- Bond) | 2.49  2.77 | Phe172, (Pi-alkyl)  Phe172, (Pi-Pi stacked)  Lys203, (Pi-alkyl)  Met128, (Pi-alkyl)  Arg239, (Pi-cation) | 5.10  4.93  4.96  5.03  4.02 | **2** | **7** | **-6.20** |
|  | **21** |  | Arg52, (H- Bond) | 2.51  2.98 | Arg239, (Pi-cation)  Lys203, (Pi-alkyl)  Ser201, (Carbon H- Bond) | 3.64  4.94  3.46 | **1** | **4** | **-5.60** |
|  | **Ciprofloxacin** | 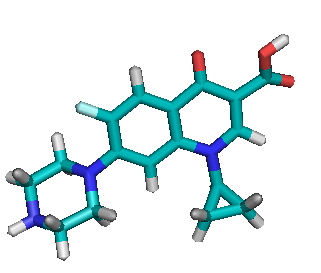 | Val49, (H- Bond)  Arg239, (H- Bond) | 2.67  2.27 | Arg219, (unfavorable)  Ser201, (halogen)  Lys203, (Carbon H- Bond)  His241, (Pi-Pi stacked)  Asn11, (Carbon H- Bond) | 2.07  3.46  3.37  5.88  3.31 | **2** | **7** | **-6.00** |

**Table S7.** Molecular interactions of ligands with amino acids of Crystal Structure of Gyrase B of *B. subtilis* (PDB: ID 4URM)

| **Protein** | **Ligand** | **3D Structure** | **Hydrophilic Interactions** | | **Hydrophobic Contacts** | | **No. of**  **H-Bonds** | **No. of**  **Total Bonds** | **affinity**  **kcal mol-1** |
| --- | --- | --- | --- | --- | --- | --- | --- | --- | --- |
|  |  |  | **Residue (H- Bond)** | **Length** | **Residue (Bond type)** | **Length** |  |  |  |
| **Gyrase B** **of *B. subtilis*** | **10** |  | Asn54, **(H- Bond)** | 2.71 | Ile51, (Pi-alkyl)  Ile175, (Pi-alkyl)  Asn54, (Amid-pi-shaped)  Ile86, (Pi-sigma) | 4.00  4.13  4.75  3.52 | 1 | 5 | **-6.90** |
|  | **23** |  | Asn54, (H- Bond) | 2.18 | Gly109,( Pi- cation)  Pro87, (Pi-alkyl)  Glu50, (Amid-pi-shaped)  Ile102, (Pi- alkyl)  Ile86, (Pi- alkyl)  Ile86, (Pi- alkyl | 3.90  4.86  4.12  4.48  4.49  4.53 | 1 | 7 | **-6.50** |
|  | **19** |  | - | - | Asp81, (Pi-anion)  Asp81, (Carbon-H)  Glu58, (Pi- anion) | 4.64  3.44  3.94 | 0 | 3 | **-6.20** |
|  | **Ciprofloxacin** | 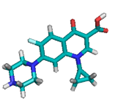 | Ser128, (H- Bond)  Ile51, (H- Bond) | **2.53**  **2.52** | Asp81, (Carbon-H)  Asn54, (Amid-pi-shaped)  Asn54, (Amid-pi-shaped)  Ile86, (Pi-Sigma)  Ile86, (Pi-Sigma)  Ile102, (Pi-alkyl)  Ile86, (Pi-alkyl) | 2.92  4.37  5.42  3.45  3.87  5.04  4.91 | 2 | 9 | **-7.20** |

**Table S8.** Molecular interactions of ligands with amino acids of Glucosamine-6-phosphate synthase in *k.pneumenia* (PDB: ID 2VF5).

| **Protein** | **Ligand** | **3D Structure** | **Hydrophilic Interactions** | | **Hydrophobic Contacts** | | **No. of**  **H-Bonds** | **No. of**  **Total Bonds** | **affinity**  **kcal mol-1** |
| --- | --- | --- | --- | --- | --- | --- | --- | --- | --- |
|  |  |  | **Residue (H- Bond)** | **Length** | **Residue (Bond type)** | **Length** |  |  |  |
| **Glucosamine-6-phosphate in pneumonia** | **23** |  | - | - | Thr237, (Carbon-H)  Trp105, (Pi- stacked)  Trp105, (Pi-stacked)  Leu167, (Pi-alkyl)  Trp105, (carbon -H)  Asn132, (carbon -H) | 3.65  5.01  5.73  5.16  2.89  2.48 | 0 | 6 | **-6.90** |
|  | **21** |  | Thr237, (H- Bond) | 2.18 | Glu276, (carbon -H)  Glu276, (carbon -H)  Trp105, (Pi- stacked) | 3.41  3.78  4.07 | 1 | 4 | **-6.20** |
|  | **22** |  | Thr237, (H- Bond)  Ser70, (H- Bond) | 2.76  1.98 | Asn132, (carbon -H)  Leu167, (Pi-alkyl)  Trp105, (Pi-alkyl) | 3.02  5.50  5.36 | 2 | 5 | **-6.20** |
|  | **Ciprofloxacin** | 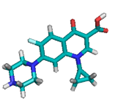 | Ser130, (H- Bond)  Asn132, (H- Bond)  Asn170, (H- Bond)  Ser70, (H- Bond)  Lys73, (H- Bond) | 2.66  2.20  2.50  1.92  6.15 | Thr237, (Carbon-H)  Trp105, (Pi- stacked)  Trp105, (Pi-stacked) | 3.10  5.86  5.17 | 5 | 8 | **-6.60** |

**Table S9.** Molecular interactions of ligands with amino acids of LasR an activator of exotoxin A expression in P. aeruginosa (PDB: ID 2UV0).

| **No** | **Protein** | **Ligand** | **3D Structure** | **Hydrophilic Interactions** | | **Hydrophobic Contacts** | | **No. of**  **H-Bonds** | **No. of**  **Total Bonds** | **affinity**  **kcal mol-1** |
| --- | --- | --- | --- | --- | --- | --- | --- | --- | --- | --- |
|  |  |  |  | **Residue (H- Bond)** | **Length** | **Residue (Bond type)** | **Length** |  |  |  |
| 1 | **LasR an activator of exotoxin of *P. aeruginosa*** | **13** | 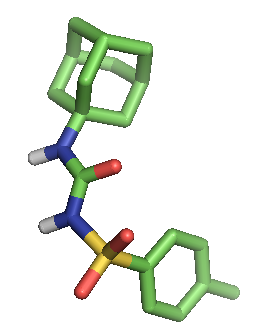 | Thr75, (H- Bond)  Thr115, (H- Bond) | 2.29  2.97 | Ala105, (Pi-alkyl)  Ala127, (Pi-alkyl)  Val76, (Pi-alkyl)  Leu110, (Pi-alkyl)  Leu40, (Pi-alkyl)  Tyr47, (Pi-alkyl)  Ala50, (Pi-alkyl)  Ala70, (Pi-alkyl)  Phe101, (Pi-Pi-T-shaped)  Trp88, (Pi-Pi-stacked)  Tyr56, (Pi-sulfur)  Asp73, (Pi-Anion) | 3.02  4.01  5.31  4.72  5.25  4.60  3.86  5.17  5.39  6.26  5.33  2.97 | 2 | 22 | **-12.50** |
| 2 |  | **23** |  | - | - | Ala70, (Pi-alkyl)  Val76, (alkyl)  Ala50, (Pi-alkyl)  Tyr47, (alkyl)  Tyr56, (Pi-Pi-T-shaped)  Ala127, (Pi-alkyl)  Asp73, (Pi-Anion) | 4.56  3.99  4.03  5.45  5.19  5.38  3.30 | 0 | 10 | **-11.30** |
| 3 |  | **21** | 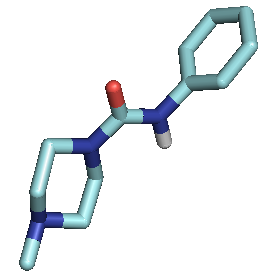 | Thr75, (H- Bond)  Thr115, (H- Bond)  Ser129, (H- Bond) | 1.85  2.66  1.98 | Val76, (alkyl)  Ala127, (Pi-alkyl)  Tyr64, (alkyl)  Trp88, (Pi-Pi-T-shaped)  Phe101, (Pi-Pi-Stacked)  Leu110, (Pi-alkyl)  Leu36, (Pi-alkyl)  Ala105, (Pi-alkyl | 5.38  4.56  5.20  5.91  5.37  4.97  4.26  4.83 | 3 | 11 | **-9.70** |
| 6 |  | **Ciprofloxacin** | 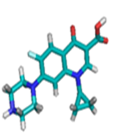 | Thr75, (H- Bond)  Thr115, (H- Bond)  Ser129, (H- Bond) | 2.23  2.79  2.72 | Leu36, (Pi-alkyl))  Ala127, (Pi-alkyl)  Ala50, (Pi-alkyl)  Ile52, (alkyl)  Ala70, (Pi-alkyl)  Tyr64, (Pi-alkyl)  Gly38, (amide-T-shaped)  Cys79, (Pi-Sulfur)  Val76, (Pi-sigma) | 5.24  4.37  4.69  4.81  4.68  4.90  5.05  5.82  3.41 | 3 | 15 | **-7.90** |

**Table S10.** Molecular interactions of ligands with amino acids of anticancer proteins.

| NO | **Protein** | **Ligand** | **3D Structure** | **Hydrophilic Interactions** | | **Hydrophobic Contacts** | | **No. of**  **H-Bonds** | **No. of**  **Total Bonds** | **affinity**  **kcal mol-1** |
| --- | --- | --- | --- | --- | --- | --- | --- | --- | --- | --- |
|  |  |  |  | **Residue (H- Bond)** | **Length** | **Residue (Bond type)** | **Length** |  |  |  |
| 1 | **CDK2** | **13** | 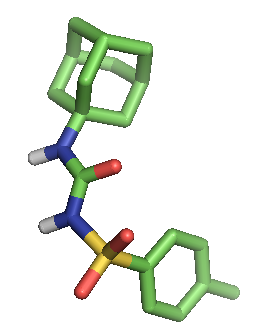 | Gln131, (H- Bond) | 3.01 | Ala31, (Pi-alkyl)  Val18, (Pi-alkyl)  Leu134, (Pi-alkyl)  Phe80, (Pi-alkyl)  Ala31, (Pi-alkyl)  Lys33, (Pi-alkyl)  Val18, (Pi-alkyl)  Leu134, (Pi-alkyl)  Gly13, (Carbon H- Bond) | 3.51  5.04  5.21  4.20  3.51  4.48  5.04  5.21  3.44 | **1** | **10** | **--8.60** |
| 2 |  | **21** | 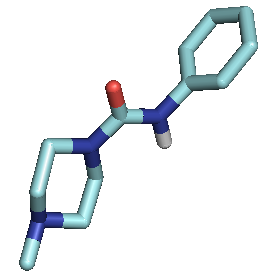 | - | - | Ile10, (Pi-sigma)  Ala31, (Pi-alkyl)  Val18, (Pi-alkyl)  Leu134, (Pi-alkyl)  Glu12, (Carbon H- Bond) | 3.72  5.10  5.26  4.57  3.59 | **0** | **5** | **-8.60** |
| 3 |  | **22** | 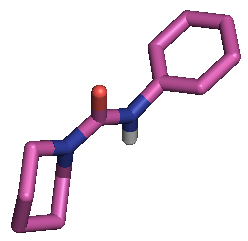 | - | - | Ile10, (Pi-sigma)  Ala31, (Pi-alkyl)  Val18, (Pi-alkyl)  Leu134, (Pi-alkyl) | 3.72  5.13  5.23  4.57 | **0** | **4** | **-8.00** |
| 4 |  | **Doxorubicin** | 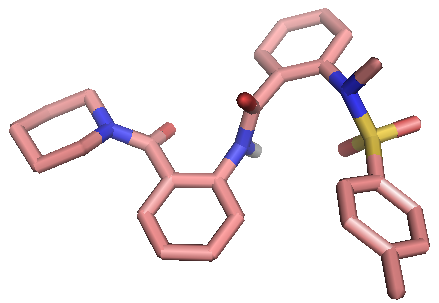 | - | - | Lys88, (Pi-alkyl)  A;a144, (Pi-alkyl)  Leu134, (Pi-alkyl)  Lys129, (Carbon H- Bond)  Lys129, (Carbon H- Bond) | 5.48  4.62  5.00  3.51  3.61 | **0** | **5** | **-7.90** |
| 5 | **EGFR** |  | 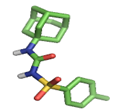 | Asp831, (H- Bond)  Asp831, (H- Bond) | 2.67  2.27 | Phe699, (Pi-Pi stacked)  Phe699, (Pi-alkyl)  Val702, (Pi-alkyl)  Leu820, (Pi-alkyl)  Ala719, (Pi-alkyl)  Lys721, ((Pi-alkyl)  Asp831, (Pi-Cation) | 3.97  4.57  3.88  5.35  3.87  4.76  3.37 | **2** | **11** | **-8.70** |
|  |  | **13** |  |  |  |  |  |  |  |  |
| 6 |  | **21** | 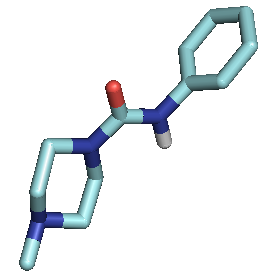 | Thr766, (H- Bond) | 2.57 | Met769, (Carbon H- Bond)  Leu694, (Pi-alkyl)  Leu820, (Pi-alkyl)  Ala719, (Pi-alkyl)  Lys721, ((Pi-cation)  Met742, (Pi-sulfur) | 3.48  5.46  4.55  4.61  5.00  4.90 | **1** | **8** | **-8.10** |
| 7 |  | **22** | 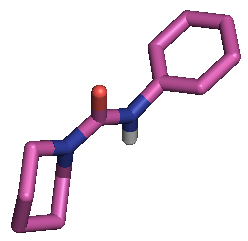 | Thr766, (H- Bond) | 2.40 | Leu694, (Pi-alkyl)  Leu820, (Pi-alkyl)  Ala719, (Pi-alkyl)  Val702, (Pi-alkyl)  Lys721, ((Pi-cation) | 5.22  4.81  4.45  5.44  4.84 | **1** | **6** | **-8.30** |
| 8 |  | **Doxorubicin** | 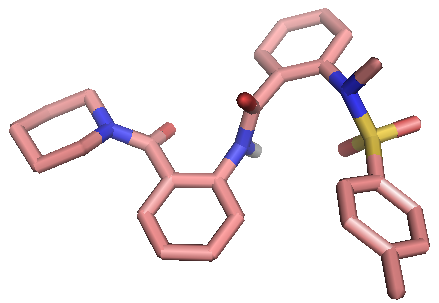 | Asn818, (H- Bond)  Met769, (H- Bond) | **2.94**  **2.36** | Thr830, (Carbon-H)  Leu694, (Carbon-H)  Val702, (Pi-Sigma)  Phe699, (Pi-Sigma)  Ala719, (Pi-alkyl)  Leu820, (Pi-alkyl)  Leu694, (Pi-Sigma)  Leu694, (Pi-Sigma) | 3.27  3.94  5.13  3.56  5.16  5.13  3.94  3.37 | 2 | 12 | **-850** |
| 9 | **ERalfa** | **13** | 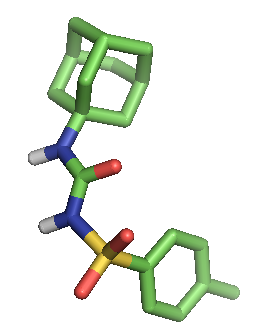 | Thr347, (H- Bond)  Met343, (H- Bond) | 2.20  2.55 | Trp383, (Pi-alkyl)  Leu746, (Pi-alkyl)  Leu525, (Pi-alkyl)  Phe404, (Pi-alkyl)  Leu349, ((Pi-alkyl)  Ala350, (Pi- alkyl) | 5.27  3.91  4.47  4.88  4.82  4.95 | **2** | **9** | **-8.80** |
| 10 |  | **21** | 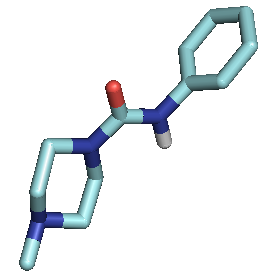 | - | - | Met421, (Pi-alkyl)  Ile424, (Pi-alkyl)  Leu391, (Pi-alkyl)  Leu525, (Pi-alkyl)  Leu346, (Pi-alkyl)  Leu349, ((Pi-alkyl)  Ala350, (Pi- alkyl)  Gly521, (Carbon H- Bond)  Gly420, (Carbon H- Bond) | 5.22  5.50  5.16  5.29  4.45  5.27  5.40  3.54  3.57 | **0** | **10** | **-7.50** |
| 11 |  | **22** | 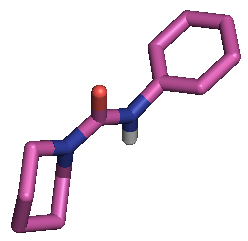 | - | - | Met421, (Pi-alkyl)  Ile424, (Pi-alkyl)  Leu391, (Pi-alkyl)  Leu525, (Pi-alkyl)  Leu346, (Pi-alkyl)  Leu349, ((Pi-alkyl)  Ala350, (Pi- alkyl) | 5.17  5.41  5.24  5.09  4.37  5.40  5.35 | **0** | **7** | **-9.20** |
| 12 |  | **Doxorubicin** | 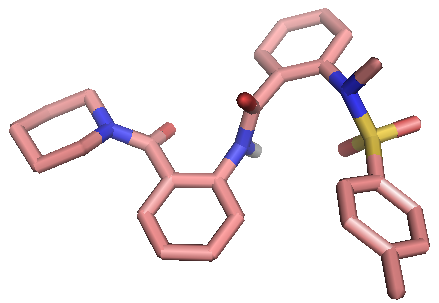 | Thr347, (H- Bond)  Met343, (H- Bond)  Glu353, (H- Bond)  Glu353, (H- Bond) | 2.15  2.58  2.64  2.38 | Ala350, (Pi- alkyl)  Leu525, (Pi-sigma)  Leu525, (Pi- alkyl)  Met343, ((Pi-sulfur)  Asp351, (Pi-Anion)  Met388, (Pi- alkyl)  Leu387, ((Pi-alkyl)  Leu428, ((Pi-alkyl) | 4.28  3.80  5.30  2.58  2.98  3.99  3.77  5.13 | 4 | 13 | **-8.10** |
| 13 | **Topoisomerase11** | **13** | 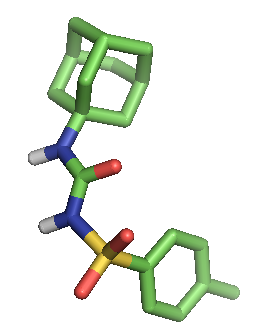 | Ser149, (H- Bond)  Lys168, (H- Bond) | 2.88  5.81 | Ser148, (Carbon H- Bond)  Lys168, (Pi-Cation)  Ile125, (Pi-alkyl) | 3.55  4.97  4.49 | **2** | **5** | **-8.90** |
| 14 |  | **21** | 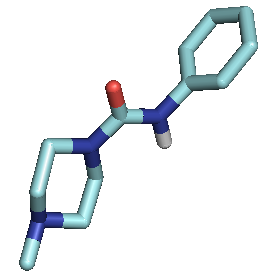 | - | - | Asn91, (Carbon H- Bond)  Ala167, (Pi-alkyl)  Ile125, (Pi-alkyl) | 3.48  5.47  5.48 | **0** | **4** | **--7.20** |
| 15 |  | **22** | 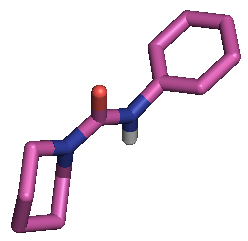 | - | - | Ala167, (Pi-alkyl)  Ile141, (Pi-alkyl)  Asn91, (Carbon H- Bond)  Asn91, (Amid-Pi Stacked) | 5.06  5.32  3.39  4.70 | **0** | **4** | **-7.90** |
| 16 |  | **Doxorubicin** | 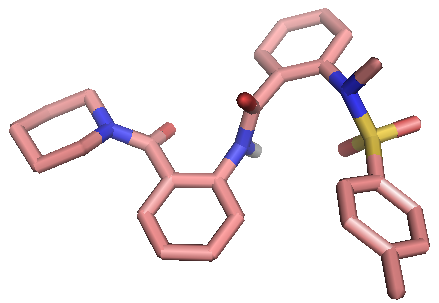 | Arg241, (H- Bond)  Arg241, (H- Bond) | 3.05  2.52 | Glu379, (Pi-Cation)  Glu379, (Pi-Cation)  Met61, (Pi-sigma)  Tyr82, (Carbon H- Bond)  Ser320, (Carbon H- Bond)  Pro79, (Pi-alkyl)  Lys83, (Pi-alkyl)  Ile311, (Pi-alkyl)  Asp245, (Unfavorable) | 3.09  3.16  3.73  3.35  3.72  5.26  5.42  5.18  2.79 | 2 | 11 | **-7.00** |
| 17 | **VEGFFR** | **13** | 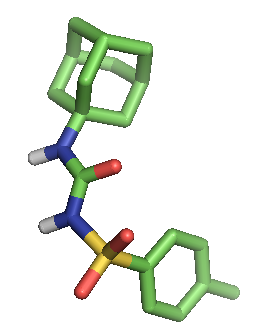 | His1026, (H-Bond)  Asp1046, (H-Bond) | 2.93  2.98 | His816, (Pi-alkyl)  Ile888, (Pi-alkyl)  Leu889, (Pi-alkyl)  Val899, (Pi-alkyl)  Leu889, (Pi-sigma)  His1026, (Carbon H- Bond) | 5.22  5.16  4.62  4.62  3.98  3.32 | **2** | **9** | **-8.90** |
| 18 |  | **21** | 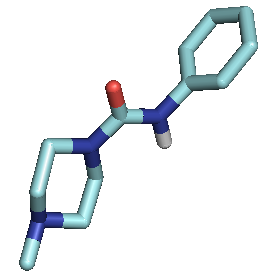 | - | - | Val899, (Pi-alkyl)  Lys868, (Pi-alkyl)  Cys1045, (Pi-alkyl)  Val916, (Pi-alkyl)  Leu840, (Pi-sigma)  Leu1035, (Pi-alkyl)  Ala866, (Pi-alkyl)  Val848, (Pi-alkyl)  Cys919, (Pi-alkyl)  Phe918, (Pi-Pi Stacked) | 4.83  4.93  4.77  4.59  4.76  4.79  4.03  5.42  5.48  5.85 | **0** | **10** | **-8.50** |
| 19 |  | **22** | 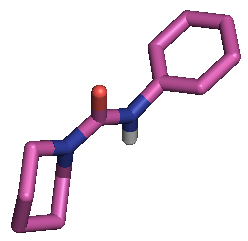 | - | - | Lys868, (Pi-alkyl)  Cys1045, (Pi-sulfur)  Val916, (Pi-alkyl)  Leu840, (Pi- alkyl)  Leu1035, (Pi-alkyl)  Ala866, (Pi-alkyl)  Val848, (Pi-alkyl)  Cys919, (Pi-alkyl)  Phe918, (Pi- alkyl) | 4.77  5.16  4.92  4.37  5.11  4.75  4.69  4.92  5.17 | **0** | **9** | **-8.80** |
| 20 |  | **Doxorubicin** | 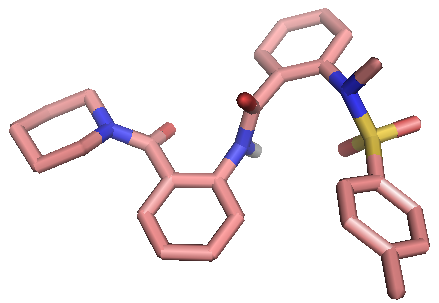 | Glu885, (H-Bond) | 2.94 | Ile888, (Pi-alkyl)  Ile888, (Pi-sigma)  Ile1025, (Unfavorable)  Leu889, (Pi-alkyl) | 5.00  3.77  2.90  5.34 | 1 | 5 | **-7.90** |

**Table S11.** Prediction of pharmacokinetics and physicochemical properties of compounds.

| **Id** | **ID** | **9** | **10** | **11** | **12** | **13** | **14** | **15** | **16** | **17** | **18** | **19** | **20** | **21** | **22** | **23** |
| --- | --- | --- | --- | --- | --- | --- | --- | --- | --- | --- | --- | --- | --- | --- | --- | --- |
| **Physicochemical Properties** | **MW** | 268.33 | 284.33 | 297.37 | 282.36 | 348.46 | 204.27 | 220.27 | 233.31 | 218.29 | 284.4 | 190.24 | 206.24 | 219.28 | 204.27 | 270.37 |
|  | **atoms** | 18 | 19 | 20 | 19 | 24 | 15 | 16 | 17 | 16 | 21 | 14 | 15 | 16 | 15 | 20 |
|  | **Heavy atoms** | 6 | 6 | 6 | 6 | 6 | 6 | 6 | 6 | 6 | 6 | 6 | 6 | 6 | 6 | 6 |
|  | **Csp3** | 0.42 | 0.42 | 0.46 | 0.46 | 0.61 | 0.42 | 0.42 | 0.46 | 0.46 | 0.61 | 0.36 | 0.36 | 0.42 | 0.42 | 0.59 |
|  | **R. bonds** | 4 | 4 | 4 | 4 | 5 | 3 | 3 | 3 | 3 | 4 | 3 | 3 | 3 | 3 | 4 |
|  | **H-BA** | 3 | 4 | 4 | 3 | 3 | 1 | 2 | 2 | 1 | 1 | 1 | 2 | 2 | 1 | 1 |
|  | **H-BD** | 1 | 1 | 1 | 1 | 2 | 1 | 1 | 1 | 1 | 2 | 1 | 1 | 1 | 1 | 2 |
|  | **MR** | 71.82 | 72.9 | 83.44 | 76.62 | 92.46 | 65.23 | 66.31 | 76.85 | 70.03 | 85.87 | 60.26 | 61.35 | 71.88 | 65.07 | 80.9 |
|  | **TPSA** | 74.86 | 84.09 | 78.1 | 74.86 | 83.65 | 32.34 | 41.57 | 35.58 | 32.34 | 41.13 | 32.34 | 41.57 | 35.58 | 32.34 | 41.13 |
| **Lipophilicity and Solubility** | **iLOGP** | 2.07 | 1.99 | 2.07 | 2.21 | 2.87 | 2.47 | 2.34 | 2.61 | 2.64 | 3.13 | 2.23 | 1.96 | 2.36 | 2.38 | 2.87 |
|  | **XLOGP3** | 1.53 | 0.67 | 0.86 | 1.89 | 4.17 | 1.96 | 1.1 | 1.29 | 2.32 | 3.97 | 1.6 | 0.74 | 0.92 | 1.95 | 3.6 |
|  | **WLOGP** | 2.19 | 1.43 | 0.96 | 2.58 | 4.03 | 2.05 | 1.29 | 0.82 | 2.44 | 3.89 | 1.74 | 0.98 | 0.51 | 2.13 | 3.59 |
|  | **MLOGP** | 1.22 | 0.41 | 0.68 | 1.49 | 2.74 | 2.26 | 1.41 | 1.68 | 2.53 | 3.78 | 1.98 | 1.13 | 1.41 | 2.26 | 3.54 |
|  | **Silicos-IT Log P** | 0.66 | 0.3 | -0.1 | 0.91 | 1.78 | 1.85 | 1.48 | 1.07 | 2.1 | 2.96 | 1.38 | 1.01 | 0.59 | 1.62 | 2.46 |
|  | **C. Log P** | 1.53 | 0.96 | 0.9 | 1.82 | 3.12 | 2.12 | 1.52 | 1.49 | 2.41 | 3.55 | 1.79 | 1.16 | 1.16 | 2.07 | 3.21 |
|  | **ESOL Log S** | -2.45 | -1.99 | -2.18 | -2.75 | -4.48 | -2.44 | -1.98 | -2.16 | -2.73 | -4.05 | -2.15 | -1.68 | -1.86 | -2.43 | -3.74 |
|  | **Ali Log S** | -2.71 | -2.01 | -2.08 | -3.08 | -5.64 | -2.26 | -1.57 | -1.64 | -2.64 | -4.53 | -1.89 | -1.19 | -1.25 | -2.25 | -4.15 |
|  | **Silicon-IT class** | Soluble | Soluble | Soluble | Soluble | Moderately | Soluble | soluble | soluble | Soluble | Moderately | soluble | soluble | soluble | Soluble | Moderately |
| **Pharmacokinetics** | **GI** | High | High | High | High | High | High | High | High | High | High | High | High | High | High | High |
|  | **BBB** | Yes | No | No | Yes | No | Yes | Yes | Yes | Yes | Yes | Yes | Yes | Yes | Yes | Yes |
|  | **Pgp substrate** | No | No | No | No | Yes | No | No | No | No | Yes | No | No | No | No | Yes |
|  | **CYP1A2** | No | No | No | No | No | No | No | No | No | No | No | No | No | No | No |
|  | **CYP2C19** | No | No | No | Yes | Yes | No | No | No | Yes | Yes | No | No | No | No | Yes |
|  | **CYP2C9** | No | No | No | No | Yes | No | No | No | No | Yes | No | No | No | No | No |
|  | **CYP2D6** | No | No | No | No | No | No | No | No | No | Yes | No | No | No | No | Yes |
|  | **CYP3A4** | No | No | No | No | Yes | No | No | No | No | No | No | No | No | No | No |
|  | **skin permeation** | -6.85 | -7.56 | -7.5 | -6.68 | -5.46 | -6.15 | -6.86 | -6.81 | -5.98 | -5.22 | -6.32 | -7.03 | -6.98 | -6.16 | -5.39 |
| **Druglikeness** | **Lipinski** | 0 | 0 | 0 | 0 | 0 | 0 | 0 | 0 | 0 | 0 | 0 | 0 | 0 | 0 | 0 |
|  | **Ghose** | 0 | 0 | 0 | 0 | 0 | 0 | 0 | 0 | 0 | 0 | 0 | 0 | 0 | 0 | 0 |
|  | **Veber** | 0 | 0 | 0 | 0 | 0 | 0 | 0 | 0 | 0 | 0 | 0 | 0 | 0 | 0 | 0 |
|  | **Egan** | 0 | 0 | 0 | 0 | 0 | 0 | 0 | 0 | 0 | 0 | 0 | 0 | 0 | 0 | 0 |
|  | **Muegge** | 0 | 0 | 0 | 0 | 0 | 0 | 0 | 0 | 0 | 0 | 1 | 0 | 0 | 0 | 0 |
|  | **Bioavailability** | 0.55 | 0.55 | 0.55 | 0.55 | 0.55 | 0.55 | 0.55 | 0.55 | 0.55 | 0.55 | 0.55 | 0.55 | 0.55 | 0.55 | 0.55 |
|  | **Leadlikeness** | 0 | 0 | 0 | 0 | 1 | 1 | 1 | 1 | 1 | 1 | 1 | 1 | 1 | 1 | 1 |
